# Supplementary material for: Genetic correlations of psychiatric traits with body composition and glycemic traits are sex- and age-dependent
Source: Nat Commun. 2019 Dec 18;10:5765. doi: 10.1038/s41467-019-13544-0 (PMC6920448; doi:10.1038/s41467-019-13544-0)
Supplement: Supplementary file 2 — Supplementary Information [file 41467_2019_13544_MOESM2_ESM.pdf]

Hübel et al. - Genetic correlations of psychiatric traits with body composition and glycemic traits are sex- and age-dependent

Supplementary Information:

Tables

Supplementary Table 1 | Phenotypic characteristics of individuals included in the UK Biobank body composition genome-wide association studies (GWASs).

Supplementary Table 2 | Exclusion criteria by International Statistical Classification of Diseases (ICD-10), British National Formulary (BNF), and UK Biobank variable

Supplementary Table 3 | Heritability estimates by linkage disequilibrium score regression, BOLT-LMM, and Haseman-Elston regression

Supplementary Table 4 | Results of the test if genetic correlations between female and male GWAS of body composition, glycemic, psychiatric, and behavioural traits are significantly different from 1.

Figures

Supplementary Fig. 1 | Putative causal associations of exposures psychiatric disorders and behavioural traits with outcomes body composition traits.

Supplementary Fig. 2a | Sex-specific genetic correlations of body composition traits and physical activity with psychiatric disorders and behavioural traits for females only.

Supplementary Fig. 2b | Sex-specific genetic correlations of body composition traits and physical activity with psychiatric disorders and behavioural traits for males only.

Supplementary Fig. 3a | Sex-specific genetic correlations of glycemic traits of psychiatric disorders and behavioural traits for females only.

Supplementary Fig. 3b | Sex-specific genetic correlations of glycemic traits of psychiatric disorders and behavioural traits for males only.

Supplementary Note 1

ADHD Working Group of the Psychiatric Genomics Consortium

Meta-Analyses of Glucose and Insulin-related traits consortium (MAGIC)

Autism Spectrum Disorder Working Group of the Psychiatrics Genomics Consortium

Bipolar Disorders Working Group of the Psychiatrics Genomics Consortium

Eating Disorders Working Group of the Psychiatric Genomics Consortium

Major Depressive Disorder Working Group of the Psychiatric Genomics Consortium

Obsessive Compulsive Disorder and Tourette Syndrome Working Group of the Psychiatric Genomics Consortium

Post-Traumatic Stress Disorder Working Group of the Psychiatric Genomics Consortium

Schizophrenia Working Group of the Psychiatric Genomics Consortium

Sex Differences Cross Disorder Working Group of the Psychiatric Genomics Consortium

Substance Use Disorder Working Group of the Psychiatric Genomics Consortium

German Borderline Genomics Consortium

The International Headache Genetics Consortium

Supplementary Table 1. Phenotypic characteristics of individuals included in the UK Biobank body composition genome-wide association studies (GWASs).

	Meta-analyzed	Female	Male
Number (%)	155,961	70,700 (45%)	85,261 (55%)
Age, years	54.9 ± 8.1	54.8 ± 8.0	55.0 ± 8.2
Height, cm	170.4 ± 9.3	163.0 ± 6.2	176.4 ± 6.7
Weight, kg	78.1 ± 15.1	69.6 ± 12.6	85.1 ± 13.2
Body mass index, kg/m2	27.0 ± 4.2	26.2 ± 4.6	27.4 ± 3.8
Waist circumference, cm	89.4 ± 12.6	82.3 ± 11.3	95.3 ± 10.3
Hip circumference, cm	102.5 ± 8.1	102.0 ± 9.3	103.0 ± 6.9
Waist-to-hip ratio	0.9 ± 0.1	0.8 ± 0.1	0.9 ± 0.1
Body fat, %	29.3 ± 8.2	35.3 ± 6.7	24.4 ± 5.5
Fat mass, kg	23.0 ± 8.5	25.3 ± 9.1	21.2 ± 7.5
Fat-free mass, kg	55.1 ±11.6	44.4 ± 4.6	63.9 ± 7.4
Socioeconomic status, Townsend Deprivation Index	-1.6 ± 2.9	-1.7 ± 2.8	-1.7 ± 2.9

Data are n (%), or mean (SD).

Alcohol consumption	Freq	% Total	% Total Cum.
Daily or almost daily	34,830	22.3	22.3
Three or four times a week	41,952	26.9	49.2
Once or twice a week	43,055	27.6	76.8
One to three times a month	16,314	10.5	87.3
Special occasions only	12,952	8.3	95.6
Never	6,858	4.4	100.0
Total	155,961	100	100

Tobacco smoking	Freq	% Total	% Total Cum.
No	142,141	91.1	91.1
Only occasionally	9,465	6.1	97.2
Yes, on most or all days	4,355	2.8	100.0
Total	155,961	100	100

**Supplementary Table 2.** Exclusion criteria by International Statistical Classification of Diseases (ICD-10), British National Formulary (BNF), and UK Biobank variable	
**International Statistical Classification of Diseases (ICD-10) categories**	
**I Certain infectious and parasitic diseases**	
A15-A19	Tuberculosis
B20-B24	Human immunodeficiency virus [HIV] disease
**II Neoplasms**	
C00-C75	Malignant neoplasms, stated or presumed to be primary, of specified sites, except of lymphoid, haematopoietic and related tissue
C00-C14	Malignant neoplasms of lip, oral cavity and pharynx
C15-C26	Malignant neoplasms of digestive organs
C30-C39	Malignant neoplasms of respiratory and intrathoracic organs
C40-C41	Malignant neoplasms of bone and articular cartilage
C43-C44	Melanoma and other malignant neoplasms of skin
C45-C49	Malignant neoplasms of mesothelial and soft tissue
C50-C50	Malignant neoplasm of breast
C51-C58	Malignant neoplasms of female genital organs
C60-C63	Malignant neoplasms of male genital organs
C64-C68	Malignant neoplasms of urinary tract
C69-C72	Malignant neoplasms of eye, brain and other parts of central nervous system
C73-C75	Malignant neoplasms of thyroid and other endocrine glands
**V Mental and behavioural disorders**	
F00-F09	Organic, including symptomatic, mental disorders
F10-F19	Mental and behavioural disorders due to psychoactive substance use
F20-F29	Schizophrenia, schizotypal and delusional disorders
F30-F39	Mood [affective] disorders
F40-F48	Neurotic, stress-related and somatoform disorders
F50-F59	Behavioural syndromes associated with physiological disturbances and physical factors
F60-F69	Disorders of adult personality and behaviour
F70-F79	Mental retardation
F80-F89	Disorders of psychological development
F90-F98	Behavioural and emotional disorders with onset usually occurring in childhood and adolescence
F99-F99	Unspecified mental disorder
**IV Endocrine, nutritional and metabolic diseases**	
E00-E07	Disorders of thyroid gland
E10-E14	Diabetes mellitus
E15-E16	Other disorders of glucose regulation and pancreatic internal secretion
E20-E35	Disorders of other endocrine glands
E70-E90	Metabolic disorders
**XI Diseases of the digestive system**	
K50-K52	Noninfective enteritis and colitis
K58	Irritable bowel syndrome

K70-K77	Diseases of liver
**XIII Diseases of the musculoskeletal system and connective tissue**	
M30-M36	Systemic connective tissue disorders
M60-63	Disorders of muscles
**Medication - British National Formulary**	
A08A	ANTIOBESITY PREPARATIONS, EXCL. DIET PRODUCTS
A10A	INSULINS AND ANALOGUES
A10B	BLOOD GLUCOSE LOWERING DRUGS, EXCL. INSULINS
A10X	OTHER DRUGS USED IN DIABETES
A14A	ANABOLIC STEROIDS
A14B	OTHER ANABOLIC AGENTS
A16A	OTHER ALIMENTARY TRACT AND METABOLISM PRODUCTS
C02L	ANTIHYPERTENSIVES AND DIURETICS IN COMBINATION
C03A	LOW-CEILING DIURETICS, THIAZIDES
C03B	LOW-CEILING DIURETICS, EXCL. THIAZIDES
C03C	HIGH-CEILING DIURETICS
C03D	POTASSIUM-SPARING AGENTS
C03E	DIURETICS AND POTASSIUM-SPARING AGENTS IN COMBINATION
C03X	OTHER DIURETICS
C07B	BETA BLOCKING AGENTS AND THIAZIDES
C07C	BETA BLOCKING AGENTS AND OTHER DIURETICS
C07D	BETA BLOCKING AGENTS, THIAZIDES AND OTHER DIURETICS
C09A	ACE INHIBITORS, PLAIN
C09B	ACE INHIBITORS, COMBINATIONS
C09C	ANGIOTENSIN II ANTAGONISTS, PLAIN
C09D	ANGIOTENSIN II ANTAGONISTS, COMBINATIONS
C09X	OTHER AGENTS ACTING ON THE RENIN-ANGIOTENSIN SYSTEM
C10A	LIPID MODIFYING AGENTS, PLAIN
C10B	LIPID MODIFYING AGENTS, COMBINATIONS
G03A	HORMONAL CONTRACEPTIVES FOR SYSTEMIC USE
G03B	ANDROGENS
G03C	ESTROGENS
G03D	PROGESTOGENS
G03E	ANDROGENS AND FEMALE SEX HORMONES IN COMBINATION
G03F	PROGESTOGENS AND ESTROGENS IN COMBINATION
G03G	GONADOTROPINS AND OTHER OVULATION STIMULANTS
G03H	ANTIANDROGENS
G03X	OTHER SEX HORMONES AND MODULATORS OF THE GENITAL SYSTEM
G04CB	Testosterone-5-alpha reductase inhibitors
H01A	ANTERIOR PITUITARY LOBE HORMONES AND ANALOGUES
H01B	POSTERIOR PITUITARY LOBE HORMONES
H01C	HYPOTHALAMIC HORMONES
H02A	CORTICOSTEROIDS FOR SYSTEMIC USE, PLAIN
H02B	CORTICOSTEROIDS FOR SYSTEMIC USE, COMBINATIONS
H02C	ANTIADRENAL PREPARATIONS
H03A	THYROID PREPARATIONS
H03B	ANTITHYROID PREPARATIONS
H04A	GLYCOGENOLYTIC HORMONES
H05A	PARATHYROID HORMONES AND ANALOGUES
H05B	ANTI-PARATHYROID AGENTS

J04AC	Hydrazides
J04AD	Thiocarbamide derivatives
J04AK	Other drugs for treatment of tuberculosis
J04AM	Combinations of drugs for treatment of tuberculosis
J04B	DRUGS FOR TREATMENT OF LEPRA
J05AE	Protease inhibitors
J05AF	Nucleoside and nucleotide reverse transcriptase inhibitors
J05AG	Non-nucleoside reverse transcriptase inhibitors
J05AR	Antivirals for treatment of HIV infections, combinations
L01A	ALKYLATING AGENTS
L01B	ANTIMETABOLITES
L01C	PLANT ALKALOIDS AND OTHER NATURAL PRODUCTS
L01D	CYTOTOXIC ANTIBIOTICS AND RELATED SUBSTANCES
L01X	OTHER ANTINEOPLASTIC AGENTS
L02A	HORMONES AND RELATED AGENTS
L02B	HORMONE ANTAGONISTS AND RELATED AGENTS
L03A	IMMUNOSTIMULANTS
L04A	IMMUNOSUPPRESSANTS
M01B	ANTIINFLAMMATORY/ANTIRHEUMATIC AGENTS IN COMBINATION
M01C	SPECIFIC ANTIRHEUMATIC AGENTS
M04A	ANTIGOUT PREPARATIONS
M05B	DRUGS AFFECTING BONE STRUCTURE AND MINERALIZATION
M09AA	Quinine and derivatives
N02BG10	Cannabinoids
N04B	DOPAMINERGIC AGENTS
N05A	ANTIPSYCHOTICS
N06A	ANTIDEPRESSANTS
N06BA	Centrally acting sympathomimetics
N06C	PSYCHOLEPTICS AND PSYCHOANALEPTICS IN COMBINATION
**UK Biobank**	
UK Biobank variable	Pregnancy
UK Biobank variable	Hysterectomie

Supplementary Table 3. Heritability as estimated by BOLT-LMM, v2.3.2, on genotyped single nucleotide polymorphisms (SNPs) on body composition traits in the UK Biobank. Variance explained by BOLT-LMM's linear predictor—using the default mixture-of-Gaussians prior on SNP effect sizes, which accounts for larger-effect SNPs—and variance theoretically explained by an optimal linear predictor, i.e., SNP-heritability (h2g). Additionally, heritability estimates and genetic correlations from linkage disequilibrium score regression (LDSC; Bulik-Sullivan et al., 2015) and Haseman-Elston regression analysis (HEreg; Yang et al., 2017).

**Phenotype**	**Sex**	**Optimal linear predictor (h2e)**	**SE**	**BOLT-LMM Optimal linear predictor (h2g)**	**SE**	**n SNPs**	**n individuals**	**LDSC h2**	**SE**	**Difference: BOLT-LDSC**	**HEreg**	**SE**
Body fat %	both	71.1%	0.4%	28.9%	0.4%	560011	155951	20.2%	0.7%	8.7%	21.9%	0.6%
	female	68.9%	0.8%	31.1%	0.8%	560011	70693	20.3%	1.0%	10.8%	22.8%	1.0%
	male	66.2%	0.7%	33.8%	0.7%	560011	85258	22.7%	0.9%	11.1%	25.2%	0.9%
	rg							0.89	0.03		0.913	0.027
Body mass index	both	70.4%	0.4%	29.6%	0.4%	560011	155951	21.2%	0.8%	8.4%	23.8%	0.7%
	female	69.7%	0.8%	30.3%	0.8%	560011	70693	19.9%	1.1%	10.4%	22.7%	1.2%
	male	64.4%	0.7%	35.6%	0.7%	560011	85258	23.5%	1.1%	12.0%	27.8%	1.2%
								0.954	####		0.946	0.027
Fat mass	both	70.9%	0.4%	29.1%	0.4%	560011	155951	20.4%	0.7%	8.7%	23.8%	0.8%
	female	68.8%	0.8%	31.2%	0.8%	560011	70693	20.5%	1.0%	10.7%	24.5%	1.4%
	male	66.7%	0.7%	33.3%	0.7%	560011	85258	21.8%	1.0%	11.5%	25.8%	1.1%
	rg							0.95	0.03		0.947	0.027
Fat-free mass	both	57.3%	0.4%	42.7%	0.4%	560011	155951	27.3%	1.1%	15.4%	43.0%	1.2%
	female	56.9%	0.8%	43.1%	0.8%	560011	70693	29.4%	1.5%	13.7%	41.3%	2.0%
	male	49.5%	0.7%	50.5%	0.7%	560011	85258	32.3%	1.7%	18.2%	48.5%	1.9%
	rg							0.95	0.02		0.962	0.016

Abbreviations: h2e = environment/noise, h2/h2g = common genetic variant heritability, LDSC = linkage disequilibrium score regression, n = number, SE = standard error, SNP = single nucleotide polymorphism

Supplementary Table 4. Results of the test if genetic correlations between female and male GWAS of body composition, glycemic, psychiatric, and behavioural traits are significantly different from 1. The correlations were calculated using linkage disequilibrium score regression (Bulik-Sullivan et. al, 2015). Standard errors were calculated by a block jackknife approach described in Hübel et al. 2018. Bonferroni-corrected p value threshold: alpha = 0.05/28 = 0.0018.
Abbreviations: BF% = body fat percentage, blocks = number of blocks used for the estimation of the jackknife standard error, BMI = body mass index, FFM = fat-free mass, FM = fat mass, HOMA-IR = Homeostatic Model Assessment for Insulin Resistance, nominally = significant at alpha = 0.02, rg = genetic correlation, se = standard error, sign = statistically significant after multiple testing correction, UKB = UK Biobank, var = variance, vs = versus

**Phenotypes**	**code**	**rg**	**se**	**blocks**	**var**	**se**	**estimate**	**z score**	**p (rg!=1)**	**sign**	**nominally**
**Female vs male**										**0.0018**	
Attention deficit hyperactivity disorder (ADHD)	ADHD05	NA		NA	NA	NA	NA	NA	NA		
Alcohol dependence	ALCD03	0.76	1.57	200	2.458	1.57	-2.18	-2.03	0.04		nominally
Anxiety	ANXI03	0.98	0.11	200	0.012	0.11	0.98	-0.22	0.82		
Autism spectrum disorder (ASD)	AUTI06	0.29	0.09	200	0.009	0.09	0.28	-7.80	6.38E-15	significant	nominally
Bipolar disorder	BIPO02	0.89	0.05	200	0.002	0.05	0.90	-2.21	0.03		nominally
Major depressive disorder (MDD)	DEPR03	0.96	0.21	200	0.104	0.32	0.85	-0.46	0.64		
Education years	EDUC01	0.92	0.02	200	0.000	0.02	0.91	-3.94	7.99E-05	significant	nominally
Insomnia	INSO01	0.78	0.12	200	0.014	0.12	0.77	-1.93	0.05		
Migraine	MIGR01	0.82	0.17	200	0.031	0.17	0.78	-1.28	0.20		
Neuroticism	NEUR03	0.99	0.06	200	0.003	0.06	0.98	-0.41	0.68		
Obsessive compulsive disorder (OCD)	OCDI01	0.98	0.51	200	0.260	0.51	0.75	-0.50	0.62		
Post-traumatic stress disorder (PTSD)	PTSD03	0.00	0.58	200	0.331	0.58	0.07	-1.62	0.11		
Schizophrenia	SCHI02	0.94	0.03	200	0.001	0.03	0.94	-2.26	0.02		nominally
**Body composition**	-	-	-	-	-	-	-	-	-	-	
**Female vs male**											
Body mass index (UKB)	BODY07	0.95	0.03	200	0.001	0.03	0.95	-1.72	0.09		
Body mass index adolescence/young adulthood	BODY12	NA		NA	NA	NA	NA	NA	NA		
Fat-free mass healthy	LEAN05	0.95	0.02	200	0.001	0.02	0.95	-2.09	0.04		nominally
Body fat % healthy	BFPC03	0.89	0.03	200	0.001	0.03	0.90	-3.50	4.67E-04	significant	nominally
Fat mass healthy	FATM03	0.95	0.03	200	0.001	0.03	0.95	-1.87	0.06		

**Age groups**											
**BMI**											
Childhood vs. adolescence	BODY09/12	1.01	0.07	200	0.005	0.07	1.01	0.10	0.92		
Childhood vs. UKB healthy	BODY09/07	0.66	0.04	200	0.001	0.04	0.66	-9.09	1.03E-19	significant	nominally
Adolescence vs UKB healthy	BODY12/07	0.80	0.05	200	0.002	0.05	0.80	-4.27	1.94E-05	significant	nominally
**Fat-free mass**											
Childhood UKB healthy	LEAN01/ LEAB05B	0.30	0.04	200	0.002	0.04	0.30	-16.53	2.33E-61	significant	nominally
**Body compartments**											
BF% vs. BMI healthy	BFPC03/ BODY07	0.82	0.01	200	0.000	0.01	0.82	-19.61	1.22E-85	significant	nominally
BF% vs FFM healthy	BFPC03/ LEAN05	0.26	0.02	200	0.001	0.02	0.29	-29.17	4.28E-187	significant	nominally
BMI vs. FFM healthy	BODY07/ LEAN05	0.52	0.02	200	0.000	0.02	0.51	-23.48	7.01E-122	significant	nominally
**Glycemic traits**											
**Female vs. male**											
HOMA-IR: Insulin resistance	GLYC16	0.76	0.16	200	0.024	0.16	0.74	-1.66	0.10		
Fasting insulin	GLYC18	0.72	0.12	200	0.015	0.12	0.70	-2.45	0.01		nominally
Fasting glucose	GLYC19	0.82	0.07	200	0.004	0.07	0.83	-2.57	0.01		nominally
**Energy expenditure**											
**Female vs. male**											
Physical activity	PHYS01	1.00	0.09	200	0.008	0.09	0.99	-0.0639	0.95		

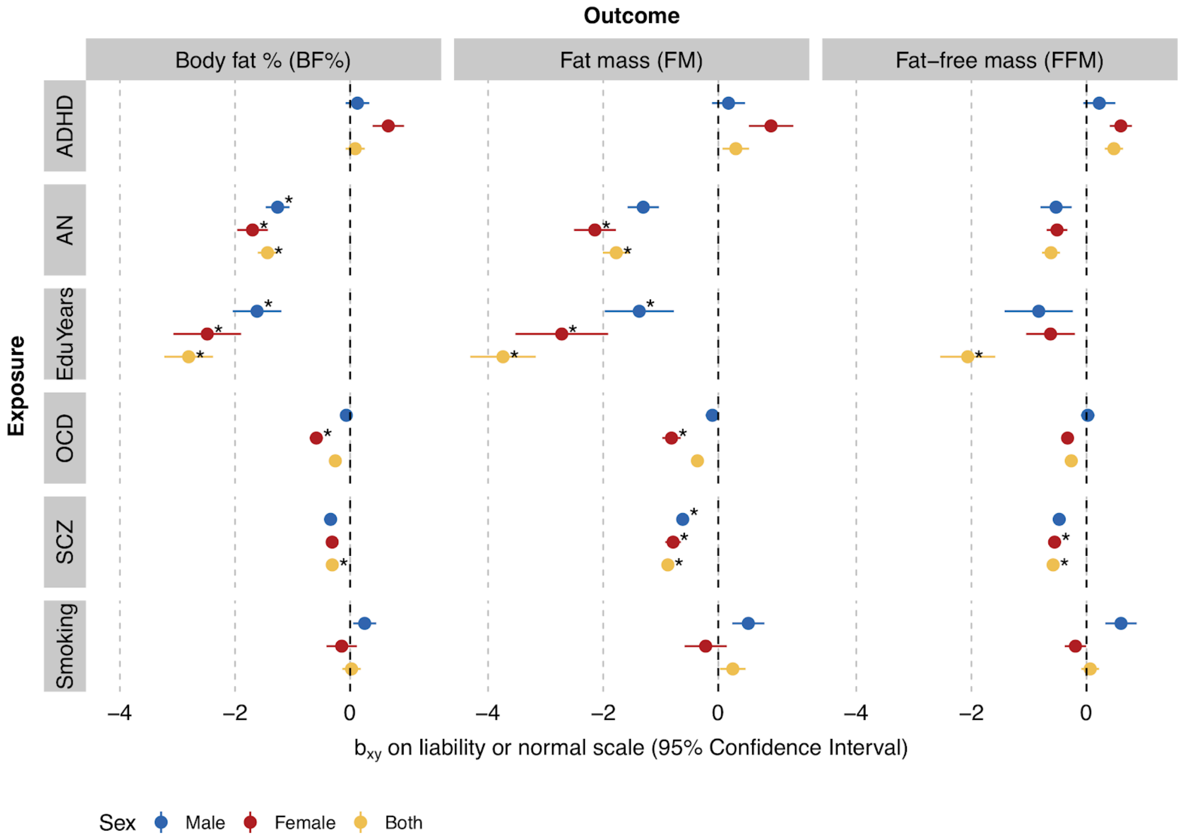

Supplementary Fig. 1 | Putative causal associations of exposures (rows) psychiatric disorders (n = up to 77,096) and behavioural traits (n = up to 217,568) with outcomes (columns) body composition traits (n = up to 155,961). Results are shown from generalised summary data-based Mendelian randomization (GSMR) analyses. Dots represent the effect sizes (as measured by betas, b_{xy}) of risk factors on disorders or traits. Colors represent the sex of the body composition trait genome-wide association study (GWAS): red for female effects, blue for male effects, and yellow for sex-combined effects. Error bars represent 95% confidence intervals (95% CIs). BF% = body fat percentage, FFM = fat-free mass, FM = fat mass, ADHD = attention-deficit/hyperactivity disorder, AN = anorexia nervosa, EduYears = education years, OCD = obsessive-compulsive disorder, SCZ = schizophrenia

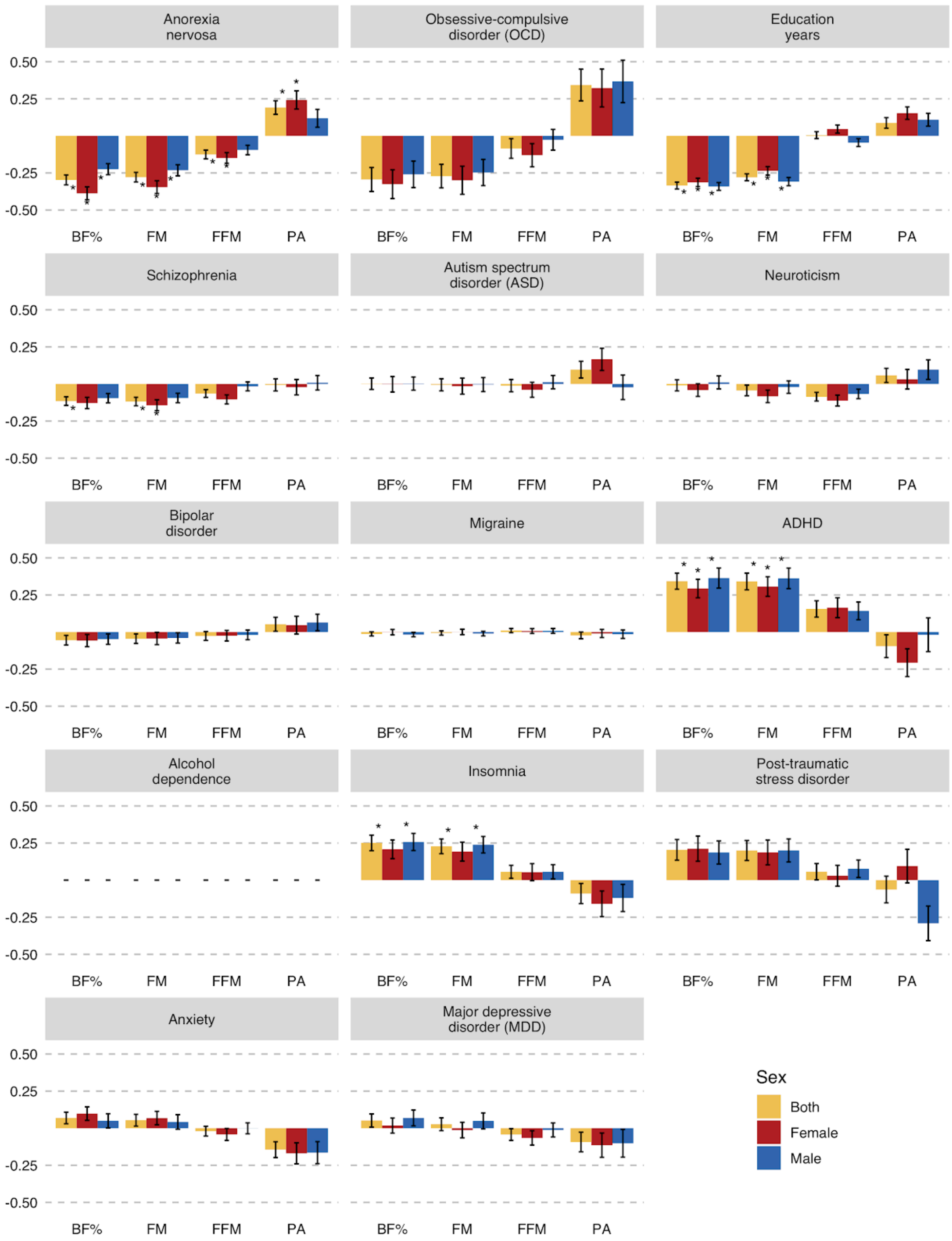

Supplementary Fig. 2a | Sex-specific genetic correlations of body composition traits (n = up to 155,961) and physical activity (n = up to 66,224) with psychiatric disorders (n = up to 35,585) and behavioural traits (n = up to 122,428) for <u>females only</u>. The autosomal genetic correlations were calculated by bivariate linkage disequilibrium score regression (LDSC). Colored bars represent genetic correlations, error bars depict standard errors (SE) and asterisks indicate statistically significant genetic correlations with p values less than α =0.0003. This threshold was calculated via the identification of the number of independent tests using matrix decomposition of the genetic correlation matrix and subsequent Bonferroni correction of α = 0.05 for 190 independent tests. ADHD = attention-deficit/hyperactivity disorder, BF% = body fat percentage, FFM = fat-free mass, FM = fat mass, PA = physical activity

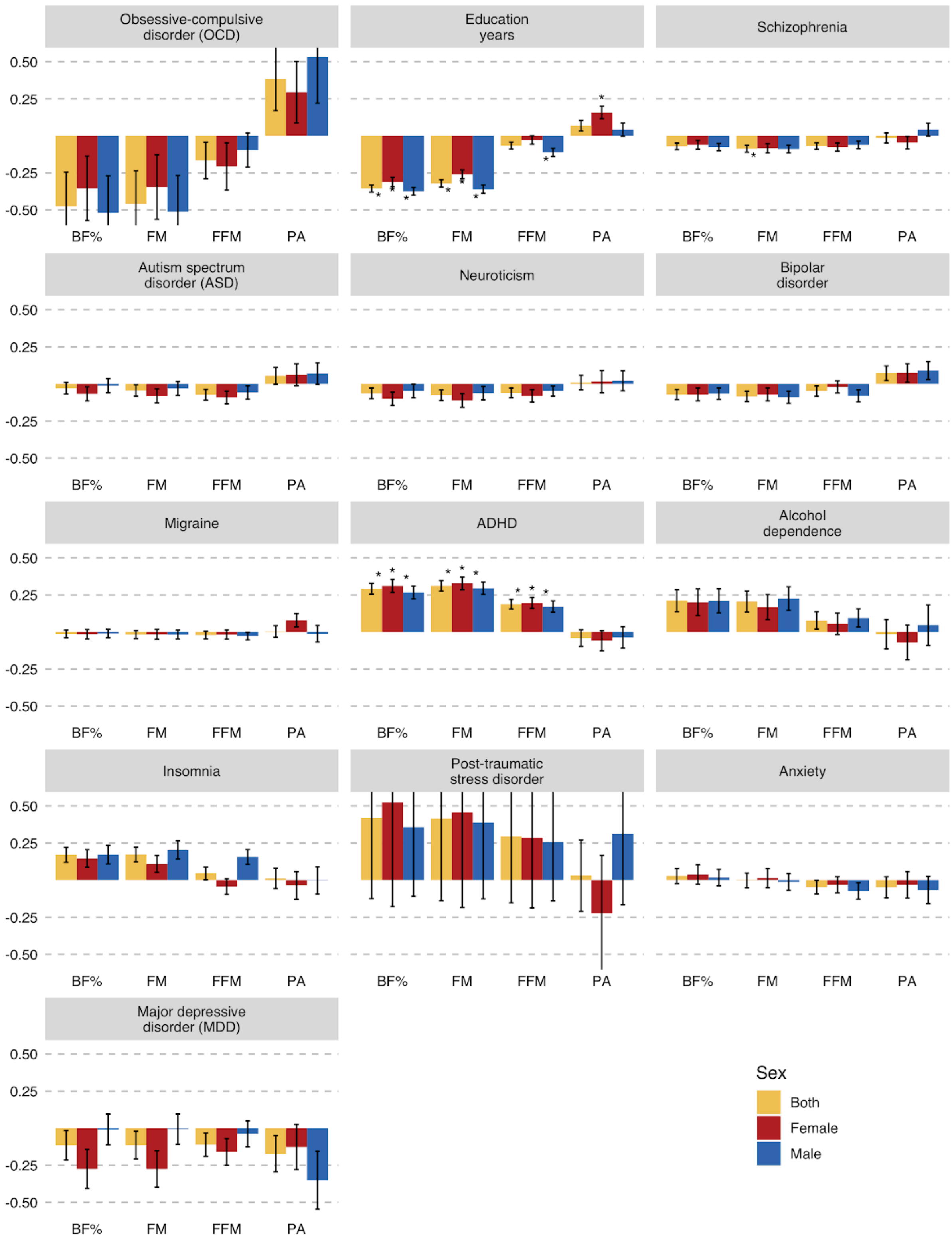

Supplementary Fig. 2b | Sex-specific genetic correlations of body composition traits (n = up to 155,961) and physical activity (n = up to 66,224) with psychiatric disorders (n = up to 45,699) and behavioural traits (n = up to 95,140) for males only. The autosomal genetic correlations were calculated by bivariate linkage disequilibrium score regression (LDSC). Colored bars represent genetic correlations, error bars depict standard errors (SE) and asterisks indicate statistically significant genetic correlations with p values less than α =0.0003. This threshold was calculated via the identification of the number of independent tests using matrix decomposition of the genetic correlation matrix and subsequent Bonferroni correction of α = 0.05 for 190 independent tests. ADHD = attention-deficit/hyperactivity disorder, BF% = body fat percentage, FFM = fat-free mass, FM = fat mass, PA = physical activity

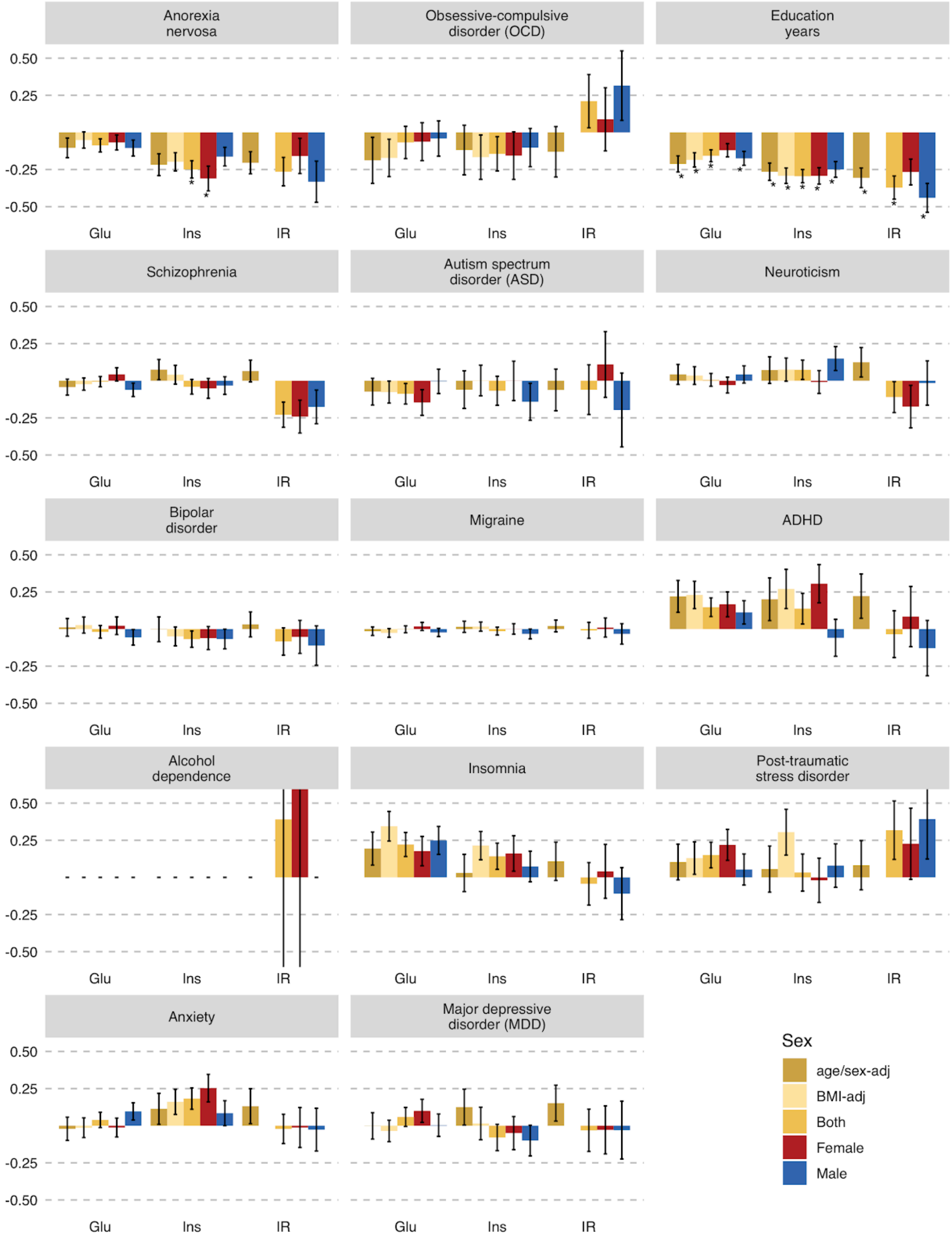

Supplementary Fig. 3a | Sex-specific genetic correlations of glycemic traits (n = up to 140,583) with psychiatric disorders (n = up to 45,699) and behavioural traits (n = up to 95,140) for <u>females only</u>. The autosomal genetic correlations were calculated by bivariate linkage disequilibrium score regression (LDSC). Colored bars represent genetic correlations, error bars depict standard errors (SE) and asterisks indicate statistically significant genetic correlations with p values less than α =0.0002. This threshold was calculated via the identification of the number of independent tests using matrix decomposition of the genetic correlation matrix and subsequent Bonferroni correction of α = 0.05 for 231 independent tests. ADHD = attention-deficit/hyperactivity disorder, Glu = fasting glucose, Ins = fasting insulin, IR = insulin resistance, adj = adjusted

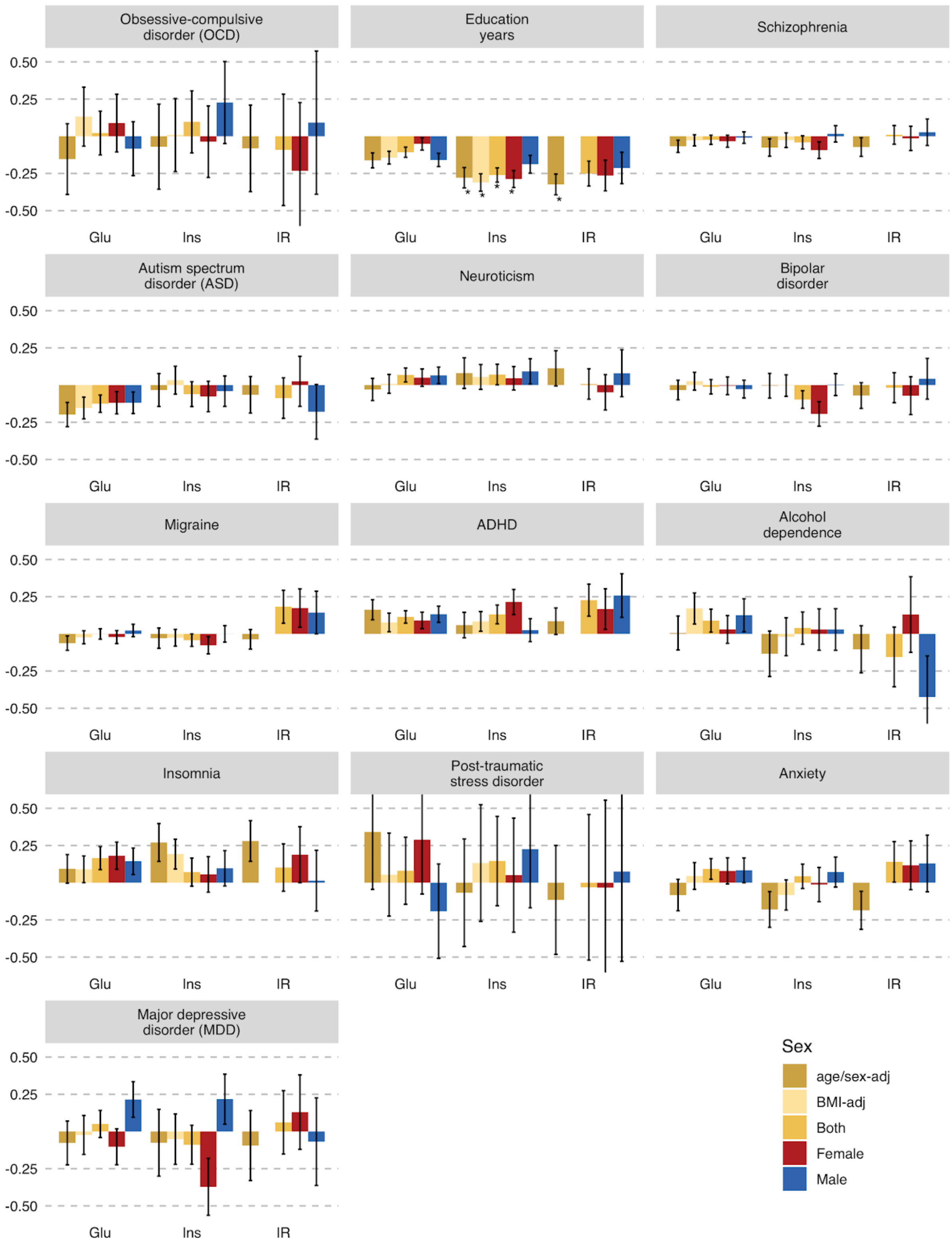

Supplementary Fig. 3b | Sex-specific genetic correlations of glycemic traits (n = up to 140,583) of psychiatric disorders (n = up to 45,699) and behavioural traits (n = up to 95,140) for males only. The autosomal genetic correlations were calculated by bivariate linkage disequilibrium score regression (LDSC). Colored bars represent genetic correlations, error bars depict standard errors (SE) and asterisks indicate statistically significant genetic correlations with p values less than α =0.0002. This threshold was calculated via the identification of the number of independent tests using matrix decomposition of the genetic correlation matrix and subsequent Bonferroni correction of α = 0.05 for 231 independent tests. ADHD = attention-deficit/hyperactivity disorder, Glu = fasting glucose, Ins = fasting insulin, IR = insulin resistance, adj = adjusted

Supplementary Note 1

Members of the ADHD Working Group of the Psychiatric Genomics Consortium and others involved in the generation of the ADHD data used in the current study

Ditte Demontis[1,2,3], Raymond K. Walters[4,5], Joanna Martin[5,6,7], Manuel Mattheisen[1,2,3,8,9,10], Thomas D. Als[1,2,3], Esben Agerbo[1,11,12], Gísli Baldursson[13], Rich Belliveau[5], Jonas Bybjerg-Grauholm[1,14], Marie Bækvad-Hansen[1,14], Felecia Cerrato[5], Kimberly Chambert[5], Claire Churchhouse[4,5,15], Ashley Dumont[5], Nicholas Eriksson[16], Michael Gandal[17,18,19,20], Jacqueline I. Goldstein[4,5,15], Katrina L. Grasby[21], Jakob Grove[1,2,3,22], Olafur O. Gudmundsson[23,24,13], Christine S. Hansen[1,14,25], Mads E. Hauberg[1,2,3], Mads V. Hollegaard[1,14], Daniel P. Howrigan[4,5], Hailiang Huang[4,5], Julian B. Maller[5,26], Alicia R. Martin[4,5,15], Nicholas G. Martin[21], Jennifer Moran[5], Jonatan Pallesen[1,2,3], Duncan S. Palmer[4,5], Carsten B. Pedersen[1,11,12], Marianne G. Pedersen[1,11,12], Timothy Poterba[4,5,15], Jesper B. Poulsen[1,14], Stephan Ripke[4,5,27], Elise B. Robinson[4,28], F. K. Satterstrom[4,5,15], Hreinn Stefansson[23], Christine Stevens[5], Patrick Turley[4,5], G. B. Walters[23,24], Hyejung Won[17,18], Margaret J. Wright[29], Ole A. Andreassen[30], Philip Asherson[31], Christie L. Burton[32], Dorret I. Boomsma[33,34], Bru Cormand[35,36,37,38], Søren Dalsgaard[11], Barbara Franke[39], Joel Gelernter[40,41], Daniel Geschwind[17,18,19], Hakon Hakonarson[42], Jan Haavik[43,44], Henry R. Kranzler[45,46], Jonna Kuntsi[31], Kate Langley[7,47], Klaus-Peter Lesch[48,49,50], Christel Middeldorp[33,51,52], Andreas Reif[53], Luis A. Rohde[54,55], Panos Roussos[56,57,58,59], Russell Schachar[32], Pamela Sklar[56,57,58], Edmund Sonuga-Barke[60], Patrick F. Sullivan[61,6], Anita Thapar[7], Joyce Y. Tung[16], Irwin D. Waldman[62], Sarah E. Medland[21], Kari Stefansson[23,24], Merete Nordentoft[1,63], David M. Hougaard[1,14], Thomas Werge[1,25,64], Ole Mors[1,65], Preben B. Mortensen[1,2,11,12], Mark J. Daly[4,5,15,66], Stephen V. Faraone[67], Anders D. Børglum[1,2,3], Benjamin M. Neale[4,5,15], Özgür Albayrak[68,69], Richard J. Anney[7], Maria J. Arranz[70], Tobias J. Banaschewski[71], Claiton Bau[55,72], Joseph Biederman[73,74], Jan K. Buitelaar[75,76], Miguel Casas[77,78,79,80], Alice Charach[81], Jennifer Crosbie[81], Astrid Dempfle[82], Alysa E. Doyle[83,84], Richard P. Ebstein[85], Josephine Elia[86,87], Christine Freitag[88], Manuel Föcker[68], Michael Gill[89], Eugenio Grevet[55,54], Ziarih Hawi[90], Johannes Hebebrand[68], Beate Herpertz-Dahlmann[91], Amaia Hervas[70], Anke Hinney[68], Sarah Hohmann[71], Peter Holmans[7], Mara Hutz[72], Abel Ickowitz[81], Stefan Johansson[92], Lindsey Kent[93], Sarah Kittel-Schneider[94], Nanda Lambregts-Rommelse[76], Gerd Lehmkuhl[95], Sandra K. Loo[96], James J. McGough[97], Jobst Meyer[98], Eric Mick[99], Frank Middletion[100], Ana Miranda[101], Nina R. Mota[55,102], Fernando Mulas[103], Aisling Mulligan[104], Freimer Nelson[18], T. T. Nguyen[105], Robert D. Oades[106], Michael C. O'Donovan[7], Michael J. Owen[7], Haukur Palmason[107], Josep A. Ramos-Quiroga[79,108,109,36], Tobias J. Renner[110,111], Marta Ribasés[79,108,36], Marcella Rietschel[112], Olga Rivero[48], Jasmin Romanos[113], Marcel Romanos[114], Aribert Rothenberger[115], Herbert Royers[116], Christina Sánchez-Mora[79,108,36], André Scherag[117,118], Benno G. Schimmelmann[119], Helmut Schäfer[105], Joseph Sergeant[120], Judith Sinzig[95,121], Susan L. Smalley[122], Hans-Christoph Steinhausen[123,124,125], Margaret Thompson[126], Alexandre Todorov[127], Alejandro A. Vasquez[128], Susanne Walitza[129,113], Yufeng Wang[130], Andreas Warnke[113], Nigel Williams[7], Stephanie H. Witt[112], Li Yang[130], Tetyana Zayats[43,4], Yanli Zhang-James[100], S. H. Lee[29,131,132], Isabell Brikell[6], Laura Ghirardi[6], Henrik Larsson[6,133], Paul Lichtenstein[6], Naomi R. Wray[29]

[1]The Lundbeck Foundation Initiative for Integrative Psychiatric Research, iPSYCH, Denmark. [2]Centre for Integrative Sequencing, iSEQ, Aarhus University, Aarhus, Denmark.

[3]Department of Biomedicine - Human Genetics, Aarhus University, Aarhus, Denmark.

[4]Analytic and Translational Genetics Unit, Department of Medicine, Massachusetts General Hospital and Harvard Medical School, Boston, Massachusetts, USA. [5]Stanley Center for Psychiatric Research, Broad Institute of Harvard and MIT, Cambridge, Massachusetts, USA.

[6]Department of Medical Epidemiology and Biostatistics, Karolinska Institutet, Stockholm,

Sweden. [7]MRC Centre for Neuropsychiatric Genetics & Genomics, School of Medicine, Cardiff University, Cardiff, United Kingdom. [8]Centre for Psychiatry Research, Department of Clinical Neuroscience, Karolinska Institutet, Stockholm, Sweden. [9]Stockholm Health Care Services, Stockholm County Council, Stockholm, Sweden. [10]Department of Psychiatry, Psychosomatics and Psychotherapy, University of Wuerzburg, Wuerzburg, Germany. [11]National Centre for Register-Based Research, Aarhus University, Aarhus, Denmark. [12]Centre for Integrated Register-based Research, Aarhus University, Aarhus, Denmark. [13]Department of Child and Adolescent Psychiatry, National University Hospital, Reykjavik, Iceland. [14]Center for Neonatal Screening, Department for Congenital Disorders, Statens Serum Institut, Copenhagen, Denmark. [15]Program in Medical and Population Genetics, Broad Institute of Harvard and MIT, Cambridge, Massachusetts, USA. [16]23andMe, Inc, Mountain View, California, USA. [17]Program in Neurogenetics, Department of Neurology, David Geffen School of Medicine, University of California, Los Angeles, Los Angeles, California, USA. [18]Center for Autism Research and Treatment and Center for Neurobehavioral Genetics, Semel Institute for Neuroscience and Human Behavior, University of California, Los Angeles, Los Angeles, California, USA. [19]Department of Human Genetics, David Geffen School of Medicine, University of California, Los Angeles, Los Angeles, California, USA. [20]Department of Psychiatry, Semel Institute for Neuroscience and Human Behavior, University of California, Los Angeles, Los Angeles, California, USA. [21]QIMR Berghofer Medical Research Institute, Brisbane, Australia. [22]Bioinformatics Research Centre, Aarhus University, Aarhus, Denmark. [23]deCODE genetics/Amgen, Reykjavík, Iceland. [24]Faculty of Medicine, University of Iceland, Reykjavík, Iceland. [25]Institute of Biological Psychiatry, MHC Sct. Hans, Mental Health Services Copenhagen, Roskilde, Denmark. [26]Genomics plc, Oxford, United Kingdom. [27]Department of Psychiatry and Psychotherapy, Charité - Universitätsmedizin, Berlin 10117, Germany. [28]Department of Epidemiology, Harvard Chan School of Public Health, Boston, Massachusetts, USA. [29]Queensland Brain Institute, University of Queensland, Brisbane, Australia. [30]NORMENT KG Jebsen Centre for Psychosis Research, Division of Mental Health and Addiction, University of Oslo and Oslo University Hospital, Oslo, Norway. [31]Social, Genetic and Developmental Psychiatry Centre, Institute of Psychiatry, Psychology and Neuroscience, King's College London, London, UK. [32]Psychiatry, Neurosciences and Mental Health, The Hospital for Sick Children, University of Toronto, Toronto, Canada. [33]Department of Biological Psychology, Neuroscience Campus Amsterdam, VU University, Amsterdam, The Netherlands. [34]EMGO Institute for Health and Care Research, Amsterdam, The Netherlands. [35]Departament de Genètica, Microbiologia i Estadística, Facultat de Biologia, Universitat de Barcelona, Barcelona, Catalonia, Spain. [36]Centro de Investigación Biomédica en Red de Enfermedades Raras (CIBERER), Instituto de Salud Carlos III, Madrid, Spain. [37]Institut de Biomedicina de la Universitat de Barcelona (IBUB), Barcelona, Catalonia, Spain. [38]Institut de Recerca Sant Joan de Déu (IRSJD), Esplugues de Llobregat, Barcelona, Catalonia, Spain. [39]Departments of Human Genetics and Psychiatry, Donders Institute for Brain, Cognition and Behaviour, Radboud University Medical Centre, Nijmegen, The Netherlands. [40]Department of Psychiatry, Genetics, and Neuroscience, Yale University School of Medicine, New Haven, Connecticut, USA. [41]Veterans Affairs Connecticut Healthcare Center, West Haven, Connecticut, USA. [42]The Center for Applied Genomics, The Children´s Hospital of Philadelphia, The Perelman School of Medicine, University of Pennsylvania, Philadelphia, PA, USA. [43]K.G. Jebsen Centre for Neuropsychiatric Disorders, Department of Biomedicine, University of Bergen, Norway. [44]Haukeland University Hospital, Bergen, Norway. [45]Department of Psychiatry, The Perelman School of Medicine, University of Pennsylvania, Philadelphia, PA, USA. [46]Veterans Integrated Service Network (VISN4) Mental Illness Research, Education, and Clinical Center (MIRECC), Crescenz VA Medical Center, Philadephia, PA, USA. [47]School

of Psychology, Cardiff University, Cardiff, United Kingdom. [48]Division of Molecular Psychiatry, Center of Mental Health, University of Wuerzburg, Wuerzburg, Germany. [49]Department of Neuroscience, School for Mental Health and Neuroscience (MHENS), Maastricht University, Maastricht, The Netherlands. [50]Laboratory of Psychiatric Neurobiology, Institute of Molecular Medicine, I.M. Sechenov First Moscow State Medical University, Moscow, Russia. [51]Child Health Research Centre, University of Queensland, Brisbane Australia. [52]Child and Youth Mental Health Service, Children's Health Queensland Hospital and Health Service, Brisbane, Australia. [53]Department of Psychiatry, Psychosomatic Medicine and Psychotherapy, University Hospital Frankfurt, Frankfurt am Main, Germany. [54]Department of Psychiatry, Faculty of Medicine, Universidade Federal do Rio Grande do Sul, Porto Alegre, Brazil. [55]ADHD Outpatient Clinic, Hospital de Clínicas de Porto Alegre, Porto Alegre, Brazil. [56]Department of Psychiatry, Icahn School of Medicine at Mount Sinai, New York, NY, USA. [57]Institute for Genomics and Multiscale Biology, Department of Genetics and Genomic Sciences, Icahn School of Medicine at Mount Sinai, New York, NY, USA. [58]Friedman Brain Institute, Department of Neuroscience, Icahn School of Medicine at Mount Sinai, New York, NY, USA. [59]Mental Illness Research Education and Clinical Center (MIRECC), James J. Peters VA Medical Center, Bronx, New York, USA. [60]Institute of Psychiatry, Psychology & Neuroscience, Kings College, London, UK. [61]Departments of Genetics and Psychiatry, University of North Carolina, Chapel Hill, NC, USA. [62]Department of Psychology, Emory University, Atlanta, Georgia, USA. [63]Mental Health Services in the Capital Region of Denmark, Mental Health Center Copenhagen, University of Copenhagen, Copenhagen, Denmark. [64]Department of Clinical Medicine, University of Copenhagen, Copenhagen, Denmark. [65]Psychosis Research Unit, Aarhus University Hospital, Risskov, Denmark. [66]Institute for Molecular Medicine Finland (FIMM), Helsinki Finland. [67]Departments of Psychiatry and Neuroscience and Physiology, SUNY Upstate Medical University, Syracuse, New York, USA. [68]Department of Child and Adolescent Psychiatry, Psychosomatics and Psychotherapy, University Hospital Essen, University of Duisburg-Essen, Essen, Germany. [69]Department of Psychosomatic Medicine and Psychotherapy, Hannover Medical School (MHH), Hannover, Germany. [70]University Hospital Mutua Terrassa, Barcelona, Spain. [71]Department of Child and Adolescent Psychiatry, Central Institute of Mental Health and Mannheim Medical Faculty, University of Heidelberg, Heidelberg, Germany. [72]Department of Genetics, Instituto de Biociências, Universidade Federal do Rio Grande do Sul, Porto Alegre, Brazil. [73]Pediatric Psychopharmacology Unit, Massachusetts General Hospital, Boston, MA, USA. [74]Department of Psychiatry, Harvard Medical School, Boston, MA, USA. [75]Department of Cognitive Neuroscience, Donders Institute for Brain, Cognition and Behavior, Radboud University Medical Centre, Nijmegen, The Netherlands. [76]Karakter Child and Adolescent Psychiatry University Center and department of Psychiatry, Donders Institute for Brain, Cognition and Behavior, Radboud University Nijmegen Medical Centre, Nijmegen, 6500 HB, The Netherlands. [77]Universitat Autònoma de Barcelona, Barcelona, Spain. [78]Programa Corporatiu "Neurodevelopment Disorders along Life Span", Institut Català de la Salut, Barcelona, Spain. [79]Department of Psychiatry, Hospital Universitari Vall d'Hebron, Barcelona, Spain. [80]Clinica Galatea y PAIMM, Mental Health Program for Impaired Physicians, Barcelona, Spain. [81]The Hospital for Sick Children, University of Toronto, Toronto, Ontario, Canada. [82]Institute of Medical Informatics and Statistics, Kiel University, Kiel, Germany. [83]Massachusetts General Hospital, Boston, MA, USA.. [84]Harvard Medical School, Boston, MA, USA.. [85]National University of Singapore, Singapore. [86]Department of Pediatrics, Nemours A.I. duPont Hospital for Children, Wilmington, DE, USA. [87]Department of Psychiatry, Sidney Kimmel Medical College, Thomas Jefferson University, Philadelphia, PA, USA. [88]Department of Child and Adolescent Psychiatry, Psychosomatics and Psychotherapy, University Hospital Frankfurt,

Goethe University, Frankfurt am Main, Germany. [89]Department of Psychiatry, Trinity College Dublin, Trinity Centre for Health Sciences, St. James's Hospital, Dublin 8, Ireland. [90]School of Psychological Sciences and Monash Institute for Cognitive and Clinical Neurosciences, Monash University, Melbourne, Australia. [91]Department of Child & Adolescent Psychiatry & Psychosomatic Medicine of University Clinics, RWTH Aachen, Aachen, Germany. [92]K.G. Jebsen Centre for Psychiatric Disorders, Department of Clinical Science, University of Bergen, Bergen, Norway. [93]University of St Andrews, St Andrews, UK. [94]Department of Psychiatry, Psychosomatic Medicine and Psychotherapy, University Hospital, Frankfurt Germany. [95]Department of Child and Adolescent Psychiatry, University of Cologne, Cologne, Germany. [96]Department of Psychiatry, University of California, Los Angeles, Los Angeles, CA, USA. [97]Semel Institute for Neuroscience & Human Behavior, David Geffen School of Medicine, University of California at Los Angeles, Los Angeles, CA, USA. [98]Institute of Psychobiology, Department of Neurobehavioral Genetics, University of Trier, Trier, Germany. [99]Quantitative Health Sciences University of Massachusetts Medical School, Worcester, MA 01655, USA. [100]Department of Psychiatry, SUNY Upstate Medical University, Syracuse, NY, USA. [101]Department of Developmental and Educational Psychology, University of Valencia, Spain. [102]Department of Human Genetics, Radboud University Medical Center, Nijmegen, Netherlands. [103]Instituto Valenciano de Neurologia Pediatrica (INVANEP), Valencia, Spain. [104]Senior Lecturer in Child and Adolescent Psychiatry, University College Dublin, Ireland. [105]University of Marburg, Marburg, Germany. [106]Clinic for Child and Adolescent Psychiatry and Psychotherapy, University of Duisburg-Essen, Essen, Germany. [107]Landspitali National University Hospital, Reykjavik, Iceland. [108]Psychiatric Genetics Unit, Group of Psychiatry, Mental Health and Addiction, Vall d'Hebron Research Institute (VHIR), Universitat Autònoma de Barcelona, Barcelona, Spain. [109]Department of Psychiatry and Legal Medicine, Universitat Autònoma de Barcelona, Barcelona, Spain. [110]Department of Child and Adolescent Psychiatry, Universitätsklinikum Tübingen, Tübingen, Germany. [111]Division of Molecular Psychiatry, ADHD Clinical Research Unit, Department of Psychiatry, Psychosomatics and Psychotherapy, University of Wuerzburg, Germany. [112]Central Institute of Mental Health, Department of Genetic Epidemiology in Psychiatry, Medical Faculty Mannheim, University of Heidelberg, Mannheim, Germany. [113]Department of Child and Adolescent Psychiatry, Psychosomatics and Psychotherapy, University of Wuerzburg, Wuerzburg, Germany. [114]University Hospital of Würzburg, Center of Mental Health, Department of Child and Adolescent Psychiatry, Psychosomatics and Psychotherapy, Wuerzburg, Germany. [115]Child and Adolescent Psychiatry/Psychotherapy, University Medical Center, Goettingen, Germany. [116]Ghent University, Dunantlaan, Ghent, B-9000 Belgium. [117]Institute for Medical Informatics, Biometry and Epidemiology (IMIBE), University of Duisburg-Essen, Essen, Germany. [118]Clinical Epidemiology, Integrated Research and Treatment Center, Center for Sepsis Control and Care (CSCC), Jena University Hospital, Jena, Germany. [119]University Hospital of Child- and Adolescent Psychiatry, University of Bern, Bern, Switzerland. [120]Vrije Universiteit, De Boelelaan, Amsterdam, The Netherlands;. [121]Department of Child and Adolescent Psychiatry and Psychotherapy, LVR – Clinic Bonn, Bonn, Germany. [122]University of California Los Angeles. [123]University of Zurich, Switzerland. [124]Aalborg University, Denmark. [125]University of Basel, Switzerland. [126]University of Southampton UK. [127]Department of Psychiatry, Washington University School of Medicine, St. Louis, Missouri, USA. [128]Department of Psychiatry & Human Genetics, Donders Institute for Brain, Cognition and Behaviour, Radboud University Medical Center, Nijmegen, The Netherlands. [129]Department of Child and Adolescent Psychiatry, University of Zurich, Zurich, Switzerland. [130]Peking University Institute of Mental Health, Beijing Shi, China. [131]School of Environmental and Rural Science, University of New England, Armidale, New South Wales,

Australia. [132]Centre for Population Health Research, School of Health Sciences and Sansom Institute of Health Research, University of South Australia, Adelaide, Australia. [133]School of Medical Sciences, Örebro University, Örebro, Sweden

Meta-Analyses of Glucose and Insulin-related traits consortium (MAGIC)
Vasiliki Lagou[1,2,3], Reedik Mägi[4], Jouke-Jan Hottenga[5,6], Harald Grallert[7,8], John R. B. Perry[9], Nabila Bouatia-Naji[10,11,12], Letizia Marullo[13], Denis Rybin[14], Rick Jansen[15], Josine L. Min[16,17], Antigone S. Dimas[18,19], Anna Ulrich[20], Liudmila Zudina[20], Jesper R. Gådin[21], Longda Jiang[20,22], Faggian Alessia[20], Joao Fadista[23], Maria G. Stathopoulou[24], Aaron Isaacs[25,26,27], Sara M. Willems[28], Pau Navarro[29], Toshiko Tanaka[30], Anne U. Jackson[31], May E. Montasser[32], Jeff R. O'Connell[32], Lawrence F. Bielak[33], Rebecca J. Webster[34], Richa Saxena[35,36,37,38], Jeanette S. Andrews[39], Beate St Pourcain[16,17], Nicholas J. Timpson[16,17], Perttu Salo[40], So-Youn Shin[41], Najaf Amin[42], Albert V. Smith[31,43,44], Guo Li[45,46], Niek Verweij[47], Anuj Goel[48], Ian Ford[49], Paul C. D. Johnson[49,50], Toby Johnson[51,52], Karen Kapur[53], Gudmar Thorleifsson[54], Rona J. Strawbridge[55,56,57], Laura J. Rasmussen-Torvik[58], Tõnu Esko[4], Evelin Mihailov[4], Tove Fall[59], Andrea Groop[60,61,62], Ross M. Fraser[63,64], Anubha Mahajan[65], Stavroula Kanoni[66], Vilmantas Giedraitis[67], Marcus E. Kleber[68], Günther Silbernagel[69], Julia Meyer[70], Martina Müller-Nurasyid[71,72,73], Andrea Ganna[60,61,62], Antti-Pekka Sarin[74,75], Loic Yengo[10,11], Dmitry Shungin[76,77,78], Jian'an Luan[9], Momoko Horikoshi[1,79,80], Ping An[81], Sanna Serena[82,83], Yvonne Boettcher[84,85], William Rayner[1,66,79], Ilja M. Nolte[86], Tatijana Zemunik[87], Erik van Iperen[88], Peter Kovacs[85], Nicholas D. Hastie[89], Sarah H. Wild[63], Stela McLachlan[63], Susan Campbell[89], Ozren Polasek[87], Olga Carlson[90], Josephine Egan[90], Wieland Kiess[85,91], Gonneke Willemsen[5], Johanna Kuusisto[92], Markku Laakso[92], Maria Dimitriou[93], Andrew A. Hicks[94], Rainer Rauramaa[95,96], Stefania Bandinelli[97], Barbara Thorand[98], Yongmei Liu[99], Iva Miljkovic[100], Lars Lind[101], Alex Doney[102], Markus Perola[40,74,103], Aroon Hingorani[104], Mika Kivimaki[104], Meena Kumari[104,105], Amanda J. Bennett[79], Christopher J. Groves[79], Christian Herder[8,106,107], Heikki A. Koistinen[108,109,110], Leena Kinnunen[108], Ulf de Faire[111], Stephan J. L. Bakker[112], Matti Uusitupa[113], Colin N. A. Palmer [102], Johan W. Jukema[114], Naveed Sattar[115], Anneli Pouta[116,117], Harold Snieder[86], Eric Boerwinkle[118,119], James S. Pankow[120], Patrik K. Magnusson[121], Ulrika Krus[122], Chiara Scapoli[13], Eco J. C. N. de Geus[5,6], Matthias Blüher[84,85], Bruce H. R. Wolffenbuttel[123], Michael A. Province[81], Goncalo R. Abecasis[31,83], James B. Meigs[61,124,125], Kees G. Hovingh[126], Jaana Lindström[127], James F. Wilson[29,63], Alan F. Wright[29], George V. Dedousis[93], Stefan R. Bornstein[128], Peter E. H. Schwarz[128], Anke Tönjes[84,85], Bernhard R. Winkelmann[129], Bernhard O. Boehm[130], Winfried März[68,131], Andres Metspalu[4], Jackie F. Price[63], Panos Deloukas[66,132,133], Antje Körner[85,91], Timo A. Lakka[95,134], Sirkka M. Keinanen-Kiukaanniemi[135,136], Timo E. Saaristo[137,138], Richard N. Bergman[139], Jaakko Tuomilehto[140,141,142,143], Nicholas J. Wareham[9], Claudia Langenberg[9], Satu Männistö[144], Paul W. Franks[77,145,146], Caroline Hayward[89], Veronique Vitart[89], Jaakko Kaprio[74,147], Sophie Visvikis-Siest[24], Beverley Balkau[148,149], David Altshuler[35,36,125], Igor Rudan[63], Michael Stumvoll[84,85], Harry Campbell[63], Cornelia M. van Duijn[25,150], Christian Gieger[151,152,153], Thomas Illig[151,154,155], Luigi Ferrucci[30], Nancy L Pedersen[121], Peter P. Pramstaller[94,156,157], Michael Boehnke[31], Timothy M. Frayling[158], Alan R. Shuldiner[32,159], Patricia A. Peyser[33], Sharon L. R. Kardia[33], Lyle J. Palmer[160], Brenda W. Penninx[15], Pierre Meneton[161], Tamara B. Harris[162], Gerjan Navis[112], Pim van der Harst[47,163], George Davey Smith[164], Nita G. Forouhi[9], Ruth J. F. Loos[9,165], Veikko Salomaa[166], Nicole Soranzo[41], Dorret I. Boomsma[5], Leif Groop[74,167,168], Tiinamaija Tuomi[74,169,170], Albert Hofman[42,171], Patricia B. Munroe[51,52], Vilmundur Gudnason[43,172], David S. Siscovick[45,46,173], Hugh Watkins[48], Cecile Lecoeur[10,11], Peter Vollenweider[174], Anders Franco-Cereceda[175], Per Eriksson[21], Marjo-Riitta Jarvelin [176,177], Kari Stefansson[54,178], Anders Hamsten[55,56,179], George Nicholson[180], Fredrik

Karpe[79,181], Emmanouil T Dermitzakis[19], Cecilia M Lindgren[1,35,79,182], Mark I. McCarthy[1,79,181], Philippe Froguel[10,11,20], Kaakinen Marika[20,183], Valeriya Lyssenko[168,184], Richard M. Watanabe[185,186,187], Erik Ingelsson[59,188,189], Jose C. Florez[36,190], Josée Dupuis[191-192], Inês Barroso[193-194], Andrew P Morris[1,103,195]

[1]Wellcome Centre for Human Genetics, University of Oxford, Oxford, United Kingdom. [2]Laboratory for Neuroimmunology, Department of Neurosciences, KU Leuven, Leuven, Belgium. VIB Center for Brain & Disease Research, Leuven, Belgium. [3]Laboratory for Translational Immunology, Department of Immunology and Microbiology, KU Leuven, Leuven, Belgium. [4]Estonian Genome Center, Institute of Genomics, University of Tartu, Tartu, Estonia. [5]Department of Biological Psychology, Vrije Universiteit, Amsterdam, the Netherlands. [6]Amsterdam Public Health research institute, VU University medical center, Amsterdam, the Netherlands. [7]Research Unit of Molecular Epidemiology, Institute of Epidemiology, Helmholtz Zentrum München Research Center for Environmental Health, Neuherberg, Germany. [8]German Center for Diabetes Research (DZD), München-Neuherberg, Germany. [9]MRC Epidemiology Unit, University of Cambridge School of Clinical Medicine, Cambridge, United Kingdom. [10]University of Lille Nord de France, Lille, France. [11]CNRS UMR8199, Institut Pasteur de Lille, Lille, France. [12]INSERM U970, Paris Cardiovascular Research Center PARCC, 75006 Paris, France. [13]Department of Life Sciences and Biotechnology, University of Ferrara, Ferrara, Italy. [14]Boston University Data Coordinating Center, Boston, Massachusetts, USA. [15]Department of Psychiatry, VU University Medical Center, Amsterdam, the Netherlands. [16]MRC Integrative Epidemiology Unit, University of Bristol, Bristol, United Kingdom. [17]Bristol Medical School, University of Bristol, Bristol, United Kingdom. [18]Biomedical Sciences Research Center "Alexander Fleming", Vari, Greece. [19]Department of Genetic Medicine and Development, University of Geneva Medical School, Geneva, Switzerland. [20]Department of Medicine, Imperial College London, London, United Kingdom. [21]Cardiovascular Medicine Unit, Center for Molecular Medicine, Department of Medicine, Karolinska Institutet, Stockholm, Karolinska University Hospital, Solna, Sweden. [22]Institute for Molecular Bioscience, The University of Queensland, Brisbane, Queensland 4072, Australia. [23]Department of Epidemiology Research, Statens Serum Institut, Copenhagen, Denmark. [24]Université de Lorraine, Inserm, IGE-PCV, F-54000, Nancy, France. [25]Genetic Epidemiology Unit, Department of Epidemiology, Erasmus Medical Center, Rotterdam, the Netherlands. [26]CARIM School for Cardiovascular Diseases, Maastricht Centre for Systems Biology (MaCSBio), Maastricht University, Maastricht, the Netherlands. [27]Department of Biochemistry, Maastricht University, Maastricht, the Netherlands. [28]Genetic Epidemiology Unit, Department of Epidemiology, Erasmus University Medical Center, Rotterdam, the Netherlands. [29]MRC Human Genetics Unit, MRC Institute of Genetics and Molecular Medicine, University of Edinburgh, Western General Hospital, Edinburgh, United Kingdom. [30]Clinical Research Branch, National Institute on Aging, Baltimore, Maryland, USA. [31]Department of Biostatistics and Center for Statistical Genetics, University of Michigan, Ann Arbor, Michigan, USA. [32]Division of Endocrinology, Diabetes, and Nutrition, Department of Medicine, University of Maryland, School of Medicine, Baltimore, Maryland, USA. [33]Department of Epidemiology, University of Michigan, Ann Arbor, Michigan, USA. [34]Laboratory for Cancer Medicine, Harry Perkins Institute of Medical Research, University of Western Australia Centre for Medical Research, Nedlands, Australia. [35]Broad Institute of Harvard and Massachusetts Institute of Technology (MIT, Cambridge, Massachusetts, USA. [36]Center for Human Genetic Research, Massachusetts General Hospital, Boston, Massachusetts, USA. [37]Department of Genetics, Harvard Medical School, Boston, Massachusetts, USA. [38]Department of Anesthesia, Critical Care and Pain Medicine, MGH, Boston, USA. [39]Department of Biostatistical Sciences,

Division of Public Health Sciences, Wake Forest University School of Medicine, Winston-Salem, North Carolina, USA. [40]Public Health Genomics Unit, Department of Chronic Disease Prevention, the National Institute for Health and Welfare, Helsinki, Finland. [41]Wellcome Trust Sanger Institute, Wellcome Trust Genome Campus, Hinxton, United Kingdom. [42]Department of Epidemiology Erasmus MC, Rotterdam, the Netherlands. [43]Icelandic Heart Association, Kopavogur, Iceland. [44]Faculty of MedicineUniversity of Iceland, Reykjavik, Iceland. [45]Cardiovascular Health Research Unit, University of Washington, Seattle, Washington, USA. [46]Department of Medicine, University of Washington, Seattle, Washington, USA. [47]Department of Cardiology, University of Groningen, University Medical Center Groningen, Groningen, The Netherlands. [48]Cardiovascular Medicine, Radcliffe Department of Medicine, University of Oxford, Oxford. [49]Robertson Centre for Biostatistics, University of Glasgow, Glasgow, United Kingdom. [50]Institute of Biodiversity, Animal Health & Comparative Medicine, University of Glasgow, Glasgow, United Kingdom. [51]Clinical Pharmacology, William Harvey Research Institute, Barts and The London School of Medicine and Dentistry, Queen Mary University of London, London, United Kingdom. [52]NIHR Barts Cardiovascular Biomedical Research Unit, Barts and The London School of Medicine and Dentistry, Queen Mary University of London, London, United Kingdom. [53]Department of Medical Genetics, University of Lausanne, Lausanne, Switzerland. [54]deCODE Genetics, Reykjavik, Iceland. [55]Cardiovascular Medicine Unit, Department of Medicine, Solna, Karolinska Institutet, Stockholm, Sweden. [56]Center for Molecular Medicine, Karolinska University Hospital Solna, Stockholm, Sweden. [57]Institute of Health and Wellbeing, University of Glasgow, Glasgow, UK. [58]Department of Preventive Medicine, Northwestern University Feinberg School of Medicine, Chicago, Illinois, USA. [59]Department of Medical Sciences, Molecular Epidemiology and Science for Life Laboratory, Uppsala University, Uppsala, Sweden. [60]Analytic and Translational Genetics Unit, Massachusetts General Hospital, Boston, Massachusetts, USA. [61]Program in Medical and Population Genetics, Broad Institute of MIT and Harvard, Cambridge, Massachusetts, USA. [62]Stanley Center for Psychiatric Research, Broad Institute of MIT and Harvard, Cambridge, Massachusetts, USA. [63]Usher Institute of Population Health Sciences and Informatics, University of Edinburgh, Edinburgh, United Kingdom. [64]Synpromics Ltd, Roslin Innovation Centre, Easter Bush Campus, Edinburgh EH25 9RG, UK. [65]Wellcome Centre for Human Genetics, University of Oxford, Oxford, United Kingdom. [66]Wellcome Trust Sanger Institute, Hinxton, United Kingdom. [67]Department of Public Health and Caring Sciences, Uppsala Universitet, Uppsala, Sweden. [68]Vth Department of Medicine, Medical Faculty Mannheim, Heidelberg University, Mannheim, Germany. [69]Division of Angiology, Department of Internal Medicine, Medical University of Graz, Austria. [70]Institute of Genetic Epidemiology, Helmholtz Zentrum München, German Research Center for Environmental Health, Neuherberg, Germany. [71]Institute of Medical Informatics, Biometry and Epidemiology, Chair of Epidemiology and Chair of Genetic Epidemiology, Ludwig-Maximilians-Universität, Munich, Germany. [72]Department of Medicine I, University Hospital Grosshadern, Ludwig-Maximilians-University, Munich, Germany. [73]Institute of Genetic Epidemiology, Helmholtz Zentrum München, German Research Center for Environmental Health, Neuherberg, Germany. [74]Institute for Molecular Medicine Finland, FIMM, University of Helsinki, Finland. [75]Public Health Genomics Unit, National Institute for Health and Welfare, Helsinki, Finland. [76]Department of Public Health & Clinical Medicine, Umeå University, Umeå, Sweden. [77]Department of Clinical Sciences, Genetic and Molecular Epidemiology Unit, Skåne University Hospital Malmö, Malmö, Sweden. [78]Department of Odontology, Umeå University, Umeå, Sweden. [79]Oxford Centre for Diabetes, Endocrinology and Metabolism, University of Oxford, Oxford, United Kingdom. [80]RIKEN, Center for Integrative Medical Sciences, Laboratory for Endocrinology, Metabolism and kidney disease,

Yokohama, Japan. [81]Division of Statistical Genomics, Washington University School of Medicine, St. Louis, Missouri, USA. [82]Istituto di Ricerca Genetica e Biomedica, CNR, Monserrato, Italy. 83 . [84]University of Leipzig, Department of Medicine, Leipzig, Germany. [85]University of Leipzig, IFB AdiposityDiseases, Leipzig, Germany. [86]Department of Epidemiology, University of Groningen, University Medical Center Groningen, Groningen, The Netherlands. [87]Faculty of Medicine, University of Split, Split, Croatia. [88]Department of Clinical Epidemiology and Biostatistics, Academic Medical Center, University of Amsterdam, Amsterdam, the Netherlands. [89]MRC Human Genetics Unit, MRC Institute of Genetics and Molecular Medicine,University of Edinburgh, Western General Hospital, Edinburgh, United Kingdom. [90]Laboratory of Clinical Investigation, National Institute of Aging, Baltimore, Maryland, USA. [91]Pediatric Research Center, Department of Women's & Child Health, University of Leipzig, Leipzig, Germany. [92]Department of Medicine, University of Eastern Finland and Kuopio University Hospital, Kuopio, Finland. [93]Department of Dietetics-Nutrition, Harokopio University, Athens, Greece. [94]Center for Biomedicine, European Academy Bozen/Bolzano (EURAC), Bolzano, Italy - Affiliated Institute of the University of Lübeck, Lübeck, Germany. [95]Kuopio Research Institute of Exercise Medicine, Kuopio, Finland. [96]Department of Clinical Physiology and Nuclear Medicine, Kuopio University Hospital, Kuopio, Finland. [97]Geriatric Unit, Azienda Sanitaria Firenze (ASF), Florence, Italy. [98]Institute of Epidemiology II, Helmholtz Zentrum München, German Research Center for Environmental Health, Neuherberg, Germany. [99]Department of Epidemiology and Prevention, Division of Public Health Sciences, Wake Forest University School of Medicine, Winston-Salem, North Carolina, USA. [100]Department of Epidemiology, Center for Aging and Population Health, University of Pittsburgh, Pittsburgh, Pennsylvania, USA. [101]Department of Medical Sciences, Uppsala University, Akademiska sjukhuset, Uppsala, Sweden. [102]Pat McPherson Centre for Pharmacogenetics and Pharmacogenomics, Division of Molecular and Clinical Medicine, Ninewells Hospital and Medical School, University of Dundee, Dundee, United Kingdom. [103]Estonian Genome Center, University of Tartu, Tartu, Estonia. [104]Department of Epidemiology and Public Health, University College London, London, United Kingdom. [105]University of Essex, Wivenhoe Park, Colchester, Essex, United Kingdom. [106]Institute of Clinical Diabetology, German Diabetes Center, Leibniz Center for Diabetes Research at Heinrich Heine University Düsseldorf, Düsseldorf, Germany. [107]Division of Endocrinology and Diabetology, Medical Faculty, Heinrich Heine University Düsseldorf, Düsseldorf, Germany. [108]Department of Public Health Solutions, National Institute for Health and Welfare, P.O. Box 30, Helsinki FI-00271, Finland. [109]University of Helsinki and Helsinki University Central Hospital: Department of Medicine, P.O. Box 340, Haartmaninkatu 4, Helsinki FI-00029, USA. [110]Minerva Foundation Institute for Medical Research, Biomedicum 2U, Tukholmankatu 8, Helsinki FI-00290, Finland. [111]Division of Cardiovascular Epidemiology, Institute of Environmental Medicine, Karolinska Institutet, Stockholm, Sweden. [112]Department of Internal Medicine, University of Groningen, University Medical Center Groningen, Groningen, The Netherlands. [113]Institute of Public Health and Clinical Nutrition, University of Eastern Finland, Kuopio, Finland. [114]Department of Cardiology C5-P, Leiden University Medical Center, Leiden, the Netherlands. [115]Institute of Cardiovascular and Medical Sciences, University of Glasgow, Glasgow, United Kingdom. [116]National Institute for Health and Welfare, Oulu, Finland. [117]Department of Clinical Sciences/Obstetrics and Gynecology, University of Oulu, Oulu, Finland. [118]IMM Center for Human Genetics, University of Texas Health Science Center at Houston, Houston, Texas, USA. [119]Division of Epidemiology, School of Public Health, University of Texas Health Science Center at Houston, Houston, Texas, USA. [120]Division of Epidemiology and Community Health, School of Public Health, University of Minnesota, Minneapolis, Minnesota, USA. [121]Department of Medical Epidemiology and Biostatistics,

Karolinska Institutet, Stockholm, Sweden. [122]Department of Clinical Sciences, Diabetes and Endocrinology Research Unit, University Hospital Malmö, Lund University, Malmö, Sweden . [123]Department of Endocrinology, University of Groningen, University Medical Center Groningen, Groningen, The Netherlands. [124]General Medicine Division, Massachusetts General Hospital, Boston, Massachusetts, USA. [125]Department of Medicine, Harvard Medical School, Boston, Massachusetts, USA. [126]Department Vascular Medicine, Academic Medical Center, Amsterdam, the Netherlands. [127]National Institute for Health and Welfare, Diabetes Prevention Unit, Helsinki, Finland. [128]Department of Medicine III, University of Dresden, Medical Faculty Carl Gustav Carus, Dresden, Germany. [129]Cardiology Group, Frankfurt-Sachsenhausen, Germany. [130]Division of Endocrinology and Diabetes, Department of Medicine, University Hospital, Ulm, Germany. [131]Synlab Academy, Synlab Holding Deutschland GmbH, Mannheim, Germany. [132]William Harvey Research Institute, Barts and The London School of Medicine and Dentistry, Queen Mary University of London, London, United Kingdom. [133]Princess Al-Jawhara Al-Brahim Centre of Excellence in Research of Hereditary Disorders (PACER-HD), King Abdulaziz University, Jeddah, Saudi Arabia. [134]Institute of Biomedicine/Physiology, University of Eastern Finland, Kuopio Campus, Kuopio, Finland. [135]Faculty of Medicine, Institute of Health Sciences, University of Oulu, Oulu, Finland. [136]Unit of General Practice, Oulu University Hospital, Oulu, Finland. [137]Finnish Diabetes Association, Tampere, Finland. [138]Pirkanmaa Hospital District, Tampere, Finland. [139]Diabetes and Obesity Research Institute, Cedars-Sinai Medical Center, Los Angeles, California, USA. [140]Department of Chronic Disease Prevention, National Institute for Health and Welfare, Helsinki, Finland. [141]Dasman Diabetes Institute, Dasman, Kuwait. [142]Centre for Vascular Prevention, Danube-University Krems, Krems, Austria. [143]Diabetes Research Group, King Abdulaziz University, Jeddah, Saudi Arabia. [144]Department of Public Health Solutions, National Institute for Health and Welfare, Helsinki, Finland. [145]Department of Nutrition, Harvard School of Public Health, Boston, Massachusetts, USA. [146]Department of Public Health & Clinical Medicine, Units of Medicine and Nutritional Research, Umeå University, Umeå, Sweden. [147]Department of Public Health, University of Helsinki, Helsinki, Finland. [148]Inserm, CESP Center for Research in Epidemiology and Public Health, U1018, , Villejuif, France. [149]Univ Paris-Saclay, Univ Paris Sud, UVSQ, UMRS 1018, , UMRS 1018, Villejuif, France. [150]Centre for Medical Systems Biology, Leiden, the Netherlands. [151]Research Unit of Molecular Epidemiology, Helmholtz Zentrum München, German Research Center for Environmental Health, Neuherberg, Germany. [152]Institute of Epidemiology II, Helmholtz Zentrum München, German Research Center for Environmental Health, Neuherberg, Germany. [153]German Center for Diabetes Research (DZD), Neuherberg, Germany. [154]Hannover Unified Biobank, Hannover Medical School, Hannover, Germany. [155]Institute of Human Genetics, Hannover Medical School, Hannover, Germany. [156]Department of Neurology, General Central Hospital, Bolzano, Italy. [157]Department of Neurology, University of Lübeck, Lübeck, Germany. [158]Genetics of Complex Traits, Peninsula Medical School, University of Exeter, United Kingdom. [159]The Regeneron Genetics Center, Regeneron Pharmaceuticals, Tarrytown, NY. [160]School of Public Health, University of Adelaide, Adelaide, Australia. [161]U872 Institut National de la Santé et de la Recherche Médicale, Centre de Recherche des Cordeliers, 75006 75006 Paris, France. [162]Geriatric Epidemiology Section, Laboratory of Epidemiology, Demography, and Biometry, National Institute on Aging, Bethesda, Maryland. [163]Department of Genetics, University Medical Center Groningen, University of Groningen, Groningen, the Netherlands. [164]MRC Integrative Epidemiology Unit (IEU), University of Bristol, Bristol, UK. [165]The Charles Bronfman Institute for Personalized Medicine, Icahn School of Medicine at Mount Sinai, New York, NY, USA. [166]National Institute for Health and Welfare, Helsinki, Finland. [167]Department of Medicine, Helsinki University Hospital, University of Helsinki, Helsinki,

Finland. [168]Department of Clinical Sciences, Diabetes and Endocrinology Research Unit, University Hospital Malmö, Lund University, Malmö, Sweden. [169]Endocrinology, Abdominal Centre, University of Helsinki and Helsinki University Hospital, Helsinki, Finland. [170]Diabetes and Obesity Research Program, University of Helsinki and Folkhälsan Research Center, Helsinki, Finland. [171]Netherlands Consortium for healthy ageing, the Hague, the Netherlands. [172]Faculty of Medicine University of Iceland, Reykjavik, Iceland. [173]Department of Epidemiology, University of Washington, Seattle, Washington, USA. [174]Department of Medicine, University Hospital Lausanne, Lausanne, Switzerland. [175]Cardiothoracic Surgery Unit, Department of Molecular Medicine and Surgery, Karolinska Institutet, Stockholm, Sweden. [176]Department of Epidemiology and Biostatistics and HPA-MRC Center, School of Public Health, Imperial College London, London, United Kingdom. [177]Institute of Health Sciences, University of Oulu, Finland. [178]Faculty of Medicine, University of Iceland, Reykjavík, Iceland. [179]Department of Cardiology, Karolinska University Hospital Solna, Stockholm, Sweden. [180]Department of Statistics, University of Oxford, Oxford, United Kingdom. [181]Oxford National Institute for Health Research Biomedical Research Centre, Churchill Hospital, Oxford, United Kingdom. [182]Big Data Institute, Li Ka Shing Centre for Health Information and Discovery, University of Oxford, Oxford, United Kingdom. [183]Department of Clinical and Experimental Medicine, School of Biosciences and Medicine, University of Surrey, Guildford, UK. [184]Department of Clinical Science, University of Bergen, Bergen, Norway. [185]Department of Preventive Medicine, Keck School of Medicine of USC, Los Angeles, California, USA. [186]Department of Physiology & Neuroscience, Keck School of Medicine of USC, Los Angeles, California, USA. [187]USC Diabetes and Obesity Research Institute, Los Angeles, California, USA. [188]Department of Medicine, Division of Cardiovascular Medicine, Stanford University School of Medicine, Stanford, California, USA. [189]Stanford Cardiovascular Institute, Stanford University, Stanford, CA 94305. [190]Diabetes Research Center, Diabetes Unit, Massachusetts General Hospital, Boston, Massachusetts, USA. [191]Department of Biostatistics, Boston University School of Public Health, Boston, Massachusetts, USA. [192]National Heart, Lung, and Blood Institute's Framingham Heart Study, Framingham, Massachusetts, USA. [193]Wellcome Trust Sanger Institute, Wellcome Trust Genome Campus, Hinxton, United Kingdom. [194]University of Cambridge Metabolic Research Laboratories and NIHR Cambridge Biomedical Research Centre, Wellcome Trust-MRC Institute of Metabolic Science, Cambridge, United Kingdom. [195]Department of Biostatistics, University of Liverpool, Liverpool, United Kingdom.

Autism Working Group of the Psychiatric Genomics Consortium

Richard J. L. Anney[1,2], Stephan Ripke[3,4,5], Verneri Anttila[3,4], Peter Holmans[1], Hailiang Huang[3,4], Lambertus Klei[6], Phil H. Lee[3,4,7], Sarah E. Medland[8], Benjamin Neale[3,4], Elise Robinson[3,4], Lauren A. Weiss[9,10], Joana Almeida[11], Thomas D. Als[12,13,14], David Amaral[15,16,17], Evdokia Anagnostou[18], Elena Bacchelli[19], Joel S. Bader[20], Marie Baekvad-Hansen[21], Anthony J. Bailey[22,23], Gillian Baird[24], Vanessa H. Bal[9], Agatino Battaglia[25], Arthur L. Beaudet[26], Raphael Bernier[27], Catalina Betancur[28,29,30], Nadia Bolshakova[2], Sven Bölte[31,32,33], Patrick F. Bolton[34,35], Anders D. Børglum[12,13,14], Thomas Bourgeron[36,37,38,39], Sean Brennan[2], Cátia Café[11], Rita M. Cantor[40,41], Jillian Casey[42,43], Patrícia B. S. Celestino-Soper[26,44], Andreas G. Chiocchetti[31], Ines C. Conceição[45,46], Judith Conroy[42,43], Catarina T. Correia[45,46], Michael L. Cuccaro[47], Geraldine Dawson[48,49], Maretha V. De Jonge[50], Silvia De Rubeis[51,52], Richard Delorme[36,37,38,53], Ditte Demontis[12,13,14], Eftichia Duketis[31], Frederico Duque[11,54], Sean Ennis[43,55], A. Gulhan Ercan-Sencicek[56], M. Daniele Fallin[57], Bridget Fernandez[58], Susan E. Folstein[59], Eric Fombonne[60], Christine M. Freitag[31], Louise Gallagher[2], John Gilbert[47], Christopher Gillberg[61], Arthur P. Goldberg[51,52], Jonas Grauholm[21], Andrew Green[43,55], Jonathan M. Green[62,63], Dorothy E. Grice[52], Jakob Grove[12,13,14,64],

Stephen J. Guter[65], Jonathan L. Haines[66], Christine S. Hansen[21], Thomas F. Hansen[13,67], Robert Hendren[9], Irva Hertz-Picciotto[15,68], Mads V. Hollegaard[21], David M. Hougaard[21], Christina M. Hultman[69], Bozenna Iliadou[69], Suma Jacob[65,70], Sabine M. Klauck[71], Alexander Kolevzon[51,52,72,73], Christine Ladd-Acosta[74], Ann S. Le Couteur[75,76], Marion Leboyer[36,77,78,79], David H. Ledbetter[80], Francesco Lescai[12,13,14], Christa Lese Martin[81], Pat Levitt[82], Catherine Lord[83], Jennifer K. Lowe[84,85,86], Elena Maestrini[19], Tiago Magalhaes[43,87], Pall Magnusson[88], Shrikant M. Mane[89], Donna M. Martin[90], Igor Martsenkovsky[91], Manuel Mattheisen[12,13,14], Susan G. McGrew[92], William M. McMahon[93], Alison Merikangas[2], Nancy Minshew[6], Anthony P. Monaco[94,95], Daniel Moreno-De-Luca[96], Eric M. Morrow[97], Ole Mors[13,98], Preben B. Mortensen[12,13,99], Susana Mouga[11,54], Michael T. Murtha[56], Merete Nordentoft[13,100], Bent Norgaard-Pedersen[21], John I. Nurnberger[44,101], Guiomar Oliveira[11,54], Alistair T. Pagnamenta[94], Jeremy R. Parr[75,76], Andrew D. Paterson[102,103,104], Milica Pejovic Milovancevic[105], Margaret A. Pericak-Vance[47], Dalila Pinto[51,52,72,73,106,107], Joseph Piven[108], Jesper Poulsen[21], Christopher S. Poultney[51,52], Fritz Poustka[31], Regina Regan[43,87], Karola Rehnström[109], Abraham Reichenberg[51,52], Jennifer Reichert[51,52], Wendy Roberts[110], Kathryn Roeder[111,112], Bernadette Rogé[113], Guy A. Rouleau[114], Evald Saemundsen[115], Stephan J. Sanders[9], Sven Sandin[69], Gerard D. Schellenberg[116], Stephen W. Scherer[102,103,117], Teimuraz Silagadze[118], Latha Soorya[51,52,119], Matthew W. State[9], Hreinn Stefansson[120], Kari Stefansson[120], Stacy Steinberg[120], Oscar Svantesson[69], Peter Szatmari[121], Ann P. Thompson[122], Kathryn Tsang[9,10], Herman van Engeland[50], Astrid M. Vicente[45,46], Veronica J. Vieland[123], Jacob A. S. Vorstman[50], Simon Wallace[22], Christopher A. Walsh[124,125,126,127,128], Regina Waltes[31], Thomas H. Wassink[129], Thomas Werge[67,130], Ellen M. Wijsman[131,132], A. Jeremy Willsey[9], Kerstin Wittemeyer[133], Timothy W. Yu[124], Lonnie Zwaigenbaum[134], Joseph D. Buxbaum[51,52,72,73,106,135], Aravinda Chakravarti[20], Edwin H. Cook[65], Hilary Coon[93], Daniel H. Geschwind[41,84,85,86], Michael Gill[2], Hakon Hakonarson[136,137], Joachim Hallmayer[138], Aarno Palotie[109], Susan Santangelo[139], James S. Sutcliffe[66,140], Dan E. Arking[20], Bernie Devlin[6], and Mark J. Daly[3,4]

[1]MRC Centre for Neuropsychiatric Genetics & Genomics, Cardiff University, Cardiff, CF24 4HQ, UK. [2]Dept. of Psychiatry, Trinity College Dublin, Dublin, D8, Ireland. [3]Analytic and Translational Genetics Unit, Dept. of Medicine, Massachusetts General Hospital and Harvard Medical School, Boston, MA 02114, USA. [4]Stanley Center for Psychiatric Research and Program in Medical and Population Genetic, Broad Institute of Harvard and MIT, Cambridge, MA 02142, USA. [5]Dept. of Psychiatry and Psychotherapy, Charité Universitätsmedizin Berlin, CCM, Berlin 10117, Germany. [6]Dept. of Psychiatry, University of Pittsburgh School of Medicine, Pittsburgh, PA 15213, USA. [7]Dept. of Psychiatry, Harvard Medical School, Boston, MA 02115, USA. [8]Queensland Institute of Medical Research, Brisbane, QLD, 4006, Australia. [9]Dept. of Psychiatry, University of California San Francisco, San Francisco, CA 94143, USA. [10]Inst. Human Genetics, University of California San Francisco, San Francisco, CA 94143, USA. [11]Unidade de Neurodesenvolvimento e Autismo do Serviço do Centro de Desenvolvimento da Criança and Centro de Investigação e Formação Clinica, Pediatric Hospital, Centro Hospitalar e Universitário de Coimbra, Coimbra, 3041-80, Portugal. [12]Dept. of Biomedicine and Human Genetics, Aarhus University, Aarhus, DK-8000, Denmark. [13]Lundbeck Foundation Initiative for Integrative Psychiatric Research (iPSYCH), Copenhagen, Denmark. [14]Centre for Integrative Sequencing (iSEQ), Aarhus University, Aarhus, DK-8000, Denmark. [15]The MIND Institute, School of Medicine, University of California Davis, Davis, CA 95817, USA. [16]Dept. of Psychiatry, School of Medicine, University of California Davis, Davis, CA 95817, USA. [17]Dept. of Behavioural Sciences, School of Medicine, University of California Davis, Davis, CA 95817, USA. [18]Bloorview Research Institute, University of Toronto, Toronto, ON, M4G 1R8,

Canada. [19]Dept. of Pharmacy and Biotechnology, University of Bologna, Bologna, 40126, Italy. [20]McKusick-Nathans Institute of Genetic Medicine, Johns Hopkins University, Baltimore, MD 21218, USA. [21]Dept. for Congenital Disorders, Center for Neonatal Screening, Statens Serum Institut, Copenhagen, DK-2300, Denmark. [22]Dept. of Psychiatry, University of Oxford and Warneford Hospital, Oxford, OX3 7JX, UK. [23]Mental Health and Addictions Research Unit, University of British Colombia, Vancouver, BC, V5Z 4H4, Canada. [24]Paediatric Neurodisability, King's Health Partners, Kings College London, London, SE1 7EH, UK. [25]Stella Maris Institute for Child and Adolescent Neuropsychiatr, Pisa, 56018, Italy. [26]Dept. of Molecular and Human Genetics, Baylor College of Medicine, Houston, TX 77030, USA. [27]Dept. of Psychiatry and Behavioral Sciences, University of Washington, Seattle, WA 98195, USA. [28]INSERM U1130, Paris, 75005, France. [29]CNRS UMR 8246, Paris, 75005, France. [30]Sorbonne Universités, UPMC Univ Paris 6, Neuroscience Paris Seine, Paris, 75005, France. [31]Dept. of Child and Adolescent Psychiatry, Psychosomatics and Psychotherapy, JW Goethe University Frankfurt, Frankfurt am Main, 60528, Germany. [32]Dept. of Women's and Children's Health, Center of Neurodevelopmental Disorders, Karolinska Institutet, Stockholm, SE-113 30, Sweden. [33]Child and Adolescent Psychiatry, Center for Psychiatry Research, Stockholm County Council, Stockholm, SE-171 77, Sweden. [34]Institute of Psychiatry, Kings College London, London, SE5 8AF, UK. [35]South London & Maudsley Biomedical Research Centre for Mental Health, London, SE5 8AF, UK. [36]FondaMental Foundation, Créteil, 94000, France. [37]Human Genetics and Cognitive Functions Unit, Institut Pasteur, Paris, 75015, France. [38]Centre National de la Recherche Scientifique URA 2182 Institut Pasteur, Paris, 75724, France. [39]University Paris Diderot, Sorbonne Paris Cité, Paris, 75013, France. [40]Dept. of Psychiatry, David Geffen School of Medicine at University of California Los Angeles, Los Angeles, CA 90095, USA. [41]Dept. of Human Genetics, David Geffen School of Medicine at University of California Los Angeles, Los Angeles, CA 90095, USA. [42]Temple Street Children's University Hospital, Dublin, D1, Ireland. [43]Academic Centre on Rare Diseases, University College Dublin, Dublin, D4, Ireland. [44]Dept. of Medical and Molecular Genetics and Program in Medical Neuroscience, Indiana University School of Medicine, Indianapolis, IN 46202, USA. [45]Instituto Nacional de Saúde Dr. Ricardo Jorge, Lisboa, 1600, Portugal. [46]Center for Biodiversity, Functional and Integrative Genomics, Campus da FCUL, Lisboa, 1649, Portugal. [47]The John P. Hussman Institute for Human Genomics, University of Miami, Miami, FL 33101, USA. [48]Duke Center for Autism and Brain Developments, Duke University School of Medicine, Durham, NC 27705, USA. [49]Duke Institute for Brain Sciences, Duke University School of Medicine, Durham, NC 27708, USA. [50]Dept. of Psychiatry, Brain Center Rudolf Magnus, University Medical Center Utrecht, Utrecht, 3584 CG, The Netherlands. [51]Seaver Autism Center for Research and Treatment, Icahn School of Medicine at Mount Sinai, New York, NY 10029, USA. [52]Dept. of Psychiatry, Icahn School of Medicine at Mount Sinai, New York, NY 10029, USA. [53]Dept. of Child and Adolescent Psychiatry, Robert Debré Hospital, Assistance Publique – Hôpitaux de Paris, Paris, 75019, France. [54]University Clinic of Pediatrics and Institute for Biomedical Imaging and Life Science, Faculty of Medicine, University of Coimbra, Coimbra, 3041-80, Portugal. [55]Centre for Medical Genetics, Our Lady's Hospital Crumlin, Dublin, D12, Ireland. [56]Programs on Neurogenetics, Yale University School of Medicine, New Haven, CT 06520, USA. [57]Dept. of Mental Health, Johns Hopkins Bloomberg School of Public Health, Baltimore, MD 21205, USA. [58]Memorial University of Newfoundland, St. John's, NL, A1B 3X9, Canada. [59]Division of Child and Adolescent Psychiatry, Dept. of Psychiatry, Miller School of Medicine, University of Miami, Miami, FL 33136, USA. [60]Dept. of Psychiatry and Institute for Development and Disability, Oregon Health & Science University, Portland, OR 97239, USA. [61]Gillberg Neuropsychiatry Centre, University of Gothenburg, Gothenburg, S-405 30, Sweden. [62]Manchester Academic Health

Sciences Centre, Manchester, M13 9NT, UK. [63]Institute of Brain, Behaviour, and Mental Health, University of Manchester, Manchester, M13 9PT, UK. [64]Bioinformatics Research Centre, Aarhus University, Aarhus, Denmark [65]Institute for Juvenile Research, Dept. of Psychiatry, University of Illinois at Chicago, Chicago, IL 60612, USA. [66]Dept. of Molecular Physiology & Biophysics, Vanderbilt University, Nashville, TN 37232, USA. [67]Institute of Biological Psychiatry, Mental Health Center, Mental Health Services Copenhagen, Copenhagen, Denmark. [68]Dept. of Public Health Sciences, School of Medicine, University of California Davis, Davis, CA 95616, USA. [69]Karolinska Institutet, Solna, SE-171 77, Sweden. [70]Institute of Translational Neuroscience and Dept. of Psychiatry, University of Minnesota, Minneapolis, MN 55454, USA. [71]Division of Molecular Genome Analysis and Working Group Cancer Genome Research, Deutsches Krebsforschungszentrum, Heidelberg, D-69120, Germany. [72]Friedman Brain Institute, Icahn School of Medicine at Mount Sinai, New York, NY 10029, USA. [73]The Mindich Child Health and Development Institute, Icahn School of Medicine at Mount Sinai, New York, NY 10029, USA. [74]Dept. of Epidemiology, Johns Hopkins Bloomberg School of Public Health, Baltimore, MD 21205, USA. [75]Institute of Neuroscience, Newcastle University, Newcastle Upon Tyne, NE2 4HH, UK. [76]Institue of Health and Science, Newcastle University, Newcastle Upon Tyne, NE2 4AX, UK. [77]INSERM U955, Paris, 94010, France. [78]Faculté de Médecine, Université Paris Est, Créteil, 94000, France. [79]Dept. of Psychiatry, Henri Mondor-Albert Chenevier Hospital, Assistance Publique – Hôpitaux de Paris, Créteil, 94000, France. [80]Chief Scientific Officer, Geisinger Health System, Danville, PA 17837, USA. [81]Autism & Developmental Medicine Institute, Geisinger Health System, Danville, PA 17837, USA. [82]Dept. of Pediatrics, Keck School of Medicine, University of Southern California, Los Angeles, CA 90027, USA. [83]Dept. of Psychiatry, Weill Cornell Medical College, Cornell University, New York, NY 10065, USA. [84]Center for Autism Research and Treatment, Semel Institute, David Geffen School of Medicine at University of California Los Angeles, Los Angeles, CA 90095, USA. [85]Program in Neurogenetics, Dept. of Neurology, David Geffen School of Medicine, University of California, Los Angeles, Los Angeles, CA 90095, USA. [86]Center for Neurobehavioral Genetics, Semel Institute, David Geffen School of Medicine, University of California, Los Angeles, Los Angeles, CA 90095, USA. [87]National Childrens Research Centre, Our Lady's Hospital Crumlin, Dublin, D12, Ireland. [88]Dept. of Child and Adolescent Psychiatry, National University Hospital, Reykjavik, IS-101, Iceland. [89]Yale Center for Genomic Analysis, Yale University School of Medicine, New Haven, CT 06516, USA. [90]Dept. of Pediatrics and Human Genetics, University of Michigan, Ann Arbor, MI 48109, USA. [91]Dept. of Child, Adolescent Psychiatry and Medical-Social Rehabilitation, Ukrainian Research Institute of Social Forensic Psychiatry and Drug Abuse, Kyiv, 04080, Ukraine. [92]Dept. of Pediatrics, Vanderbilt University, Nashville, TN 37232, USA. [93]Dept. of Psychiatry, University of Utah, Salt Lake City, UT 84108, USA. [94]Wellcome Trust Centre for Human Genetics, Oxford University, Oxford, OX3 7BN, UK. [95]Tufts University, Boston, MA 02155, USA. [96]Dept. of Psychiatry, Yale University School of Medicine, New Haven, CT 06511, USA. [97]Dept. of Psychiatry and Human Behaviour, Brown University, Providence, RI 02912, USA. [98]Aarhus University Hospital, Risskov, DK-8240, Denmark. [99]National Centre for Register-based Research, University. [100]Mental Health Services in the Capital Region of Denmark, Mental Health Center, Mental Health Services Copenhagen, Copenhagen, Denmark. [101]Institute of Psychiatric Research, Dept. of Psychiatry, Indiana University School of Medicine, Indianapolis, IN 46202, USA. [102]Dept. of Molecular Genetics, University of Toronto, Toronto, ON, M5S 1A8, Canada. [103]The Centre for Applied Genomics, The Hospital for Sick Children, Toronto, ON, M5G 1L4, Canada. [104]Dalla Lana School of Public Health, Toronto, ON, M5T 3M7, Canada. [105]Institute of Mental Health and Medical Faculty, University of Belgrade, Belgrade, 11 000, Serbia. [106]Dept. of Genetics and Genomic Sciences, Icahn

School of Medicine at Mount Sinai, New York, NY 10029, USA. [107]The Icahn Institute for Genomics and Multiscale Biology, Icahn School of Medicine at Mount Sinai, New York, NY 10029, USA. [108]University of North Carolina, Chapel Hill, NC 27599, USA. [109]Sanger Institute, Hinxton, CB10 1SA, UK. [110]Autism Research Unit, The Hospital for Sick Children, Toronto, ON, M5G 1L4, Canada. [111]Dept. of Computational Biology, Carnegie Mellon University, Pittsburgh, PA 15213, USA. [112]Dept. of Statistics, Carnegie Mellon University, Pittsburgh, PA 15213, USA. [113]Centre d'Etudes et de Recherches en Psychopathologie, Toulouse University, Toulouse, 31058, France. [114]Montreal Neurological Institute, Dept of Neurology and Neurosurgery, McGill University, Montreal, QC, H3A 2B4, Canada. [115]State Diagnostic and Counseling Centre, Kopavogur, IS-201, Iceland. [116]Dept. of Pathology and Laboratory Medicine, University of Pennsylvania, Philadelphia, PA 19102, USA. [117]McLaughlin Centre, University of Toronto, Toronto, ON, M5G 0A4, Canada. [118]Dept. of Psychiatry and Drug Addiction, Tbilisi State Medical University, Tbilisi, 0186, Georgia. [119]Dept. of Psychiatry, Rush University Medical Center, Chicago, IL 60612, USA. [120]deCODE Genetics, Reykjavik, IS-101, Iceland. [121]Dept. of Psychiatry, University of Toronto, ON, M5T 1R8, Canada. [122]Dept. of Psychiatry and Behavioral Neurosciences, McMaster University, Hamilton, ON, L8S 4L8, Canada. [123]Battelle Center for Mathematical Medicine, The Research Institute at Nationwide Children's Hospital, Columbus, OH 43205, USA. [124]Division of Genetics, Children's Hospital Boston, Harvard Medical School, Boston, MA 02115, USA.[125]Program in Genetics and Genomics, Harvard Medical School, Boston, MA 02115, USA. [126]Howard Hughes Medical Institute, Harvard Medical School, Boston, MA 02115, USA. [127]Dept. of Pediatrics, Harvard Medical School, Boston, MA 02115, USA. [128]Dept. of Neurology, Harvard Medical School, Boston, MA 02115, USA. [129]Dept. of Psychiatry, Carver College of Medicine, Iowa City, IA 52242, USA. [130]Dept. of Clinical Medicine, University of Copenhagen, Copenhagen, DK-2200, Denmark. [131]Dept. of Medicine, University of Washington, Seattle, WA 98195, USA. [132]Dept. of Biostatistics, University of Washington, Seattle, WA 98195, USA. [133]School of Education, University of Birmingham, Birmingham, B15 2TT, UK. [134]Dept. of Pediatrics, University of Alberta, Edmonton, AB, T6G 1C9, Canada. [135]Dept. of Neuroscience, Icahn School of Medicine at Mount Sinai, New York, NY 10029, USA. [136]The Center for Applied Genomics and Division of Human Genetics, Children's Hospital of Philadelphia, University of Pennsylvania School of Medicine, Philadelphia, PA 19104, USA. [137]Dept of Pediatrics, University of Pennsylvania, Philadelphia, PA 19104, USA. [138]Dept. of Psychiatry, Stanford University, Stanford, CA 94305, USA. [139]Maine Medical Center Research Institute, Portland, ME 04074, USA. [140]Center for Human Genetics Research, Vanderbilt University, Nashville, TN 37232, USA.

Bipolar Disorder Working Group of the Psychiatric Genomics Consortium

Alessandro Serretti[1], Andreas J. Forstner[2,3,4,5,6], Anil P. Ori[7,8], Annelie Nordin Adolfsson[9], Bruno Etain[10,11,12,13], Céline S. Reinbold[2,6], Christina M. Hultman[14], Claudia Giambartolomei[15], David Curtis[16,17,18], Dennis Hellgren[14], Diego Albani[19], Eduard Vieta[20], Eli A. Stahl[21,22,23], Erlend Bøen[24], Euijung Ryu[25], Fabian Streit[26,27], Frank Bellivier[10,28,11,12], Guy A. Rouleau[29,30], Howard J. Edenberg[31], Hreinn Stefansson[27], Ingrid Agartz[32,24,33], James A. Knowles[34,35], James L. Kennedy[36,37,38,39], Jana Strohmaier[26], Jens Treutlein[26], Joanna M. Biernacka[25], John B. Vincent[40], Wei Xu[41,42], Jolanta Lissowska[43], Josef Frank[26], Jurgen Del-Favero[44], Kari Stefansson[27,45,46,47], Danielle Posthuma[47,48], Laura J. Scott[18], Lilijana Oruc[49], Loes M. Olde Loohuis[7], Marcella Rietschel[26], Maria Grigoroiu-Serbanescu[50], Mark Frye[51], Amanda L. Dobbyn[21,22], Laura Huckins[21,22], Jessica S. Johnson[21,22], Markus Leber[52], Michael Boehnke[18], Mikael Landén[14,53], Nelson B. Freimer[7], Olav B. Smeland[54,55,56,57,58], Pamela Sklar[21,22], Panos Roussos[21,22,59], Per Hoffmann[2,4,6], Peter Zandi[60], Robert Karlsson[14], Patrick

F. Sullivan[14,61,62], Rolf Adolfsson[9], Sarah E. Bergen[14], Sascha B. Fischer[2,6], Simon Xi[63], Srdjan Djurovic[64,65], Dominic Holland[54,66], Stefan Herms[2,4,6], Anders M. Dale[54,67,68,46], Stephanie H. Witt[26], Sven Cichon[2,4,6,69], Thomas W. Mühleisen[2,69], Thorgeir E. Thorgeirsson[27], Torbjørn Elvsåshagen[70,71], Ney Alliey-Rodriguez[72], Elliot S. Gershon[72,73], Maria Hipolito[74], William B. Lawson[74], Evaristus A. Nwulia[74], J Raymond DePaulo[75], James B. Potash[75], Fernando Goes[75], Pamela B. Mahon[75,76], William Bunney[77], Vassily Trubetskoy[78], Swapnil Awasthi[78], Michael Bauer[79], Andrea Pfennig[79], Wolfgang Maier[80], Julie Garnham[81], Claire O'Donovan[81], Claire Slaney[81], Martin Alda[81,82], Eystein Stordal[83,84], Alexander W. Charney[22], Shaun M. Purcell[22,76], René S. Kahn[22], Piotr M. Czerski[85], Joanna Hauser[85], Gustavo Turecki[86], Pablo Cervantes[87,88], Cristiana Cruceanu[87,89], Nathalie Brunkhorst-Kanaan[90], Murielle Brum[90], Sarah Kittel-Schneider[90], Andreas Reif[90], Tiffany A. Greenwood[68], Tatyana Shehktman[68], Paul D. Shilling[68], John Kelsoe[68], Caroline M. Nievergelt[68,91], Melvin G. McInnis[92], Robert C. Thompson[92], Stanley J. Watson[92], Sebastian Zöllner[92], Bernhard T. Baune[93], Udo Dannlowski[93], John S. Strauss[38,40], John P. Rice[94], Jack D. Barchas[95], Martin Hautzinger[96], Bertram Müller-Myhsok[89,97,98], Arne E. Vaaler[99], Derek W. Morris[100,101], Ole A. Andreassen[55,56], Ingrid Melle[102,103], Ketil J. Oedegaard[104,105], Andrew McQuillin[106], Nicholas Bass[106], Toni-Kim Clarke[107], Douglas H. Blackwood[107], Andrew M. McIntosh[107,108], Lili Milani[109], Andres Metspalu[109,110], Marion Leboyer[111,10,112], Engilbert Sigurdsson[113], James D. McKay[114], Scott D. Gordon[115], Sarah E. Medland[115], Nicholas G. Martin[115,116], Shawn E. Levy[117], Richard M. Myers[117], Sara A. Paciga[118], Francis J. McMahon[119], James Boocock[120], Helena Medeiros[35], Michele T. Pato[35], Carlos Pato[35,121], David St Clair[122], Maciej Trzaskowski[123], Enda M. Byrne[123], Grant W. Montgomery[123], Yunpeng Wang[124,125], Peng Zhang[126], Franziska Degenhardt[4], Anna C. Koller[4], Anna Maaser[4], Markus M. Nöthen[4], Monika Budde[127], Urs Heilbronner[127], Katrin Gade[127,128], Thomas G. Schulze[127,75,26,128,119], Cristina Sánchez-Mora[129,130,131], Marta Ribasés[129,130,131], Maria Soler Artigas[129,130,131], Miquel Casas[129,130,132,131], Josep A. Ramos-Quiroga[129,130,132,131], Marie Bækvad-Hansen[133,134], Jonas Bybjerg-Grauholm[133,134], Christine Søholm Hansen[133,134], David M. Hougaard[133,134], Ole Mors[133,135], Merete Nordentoft[133,136], Esben Agerbo[133,137,138], Carsten Bøcker Pedersen[133,137,138], Marianne Giørtz Pedersen[133,137,138], Thomas Werge[133,139,140], Thomas D. Als[141,142,133], Anders D. Børglum[141,142,133], Jakob Grove[141,142,133,143], Preben B. Mortensen[141,133,137,138], Manuel Mattheisen[141,142,32,144,133], Manolis Kogevinas[145], Susanne Lucae[146], Tune H. Pers[23,147], Benjamin M. Neale[23,148,149], Tõnu Esko[23,150,109,151], Peter A. Holmans[152], Alexander L. Richards[152], Valentina Escott-Price[152], Liz Forty[152], Christine Fraser[152], Marian L. Hamshere[152], Nicholas Craddock[152], Ian Jones[152], George Kirov[152], Michael C. O'Donovan[152], Michael J. Owen[152], Arianna Di Florio[152,62], Douglas M. Ruderfer[153], José Guzman-Parra[154], Fabio Rivas[154], Fermin Mayoral[154], Gunnar Morken[155,156], Donald J. MacIntyre[157,158], Margit Burmeister[159], Huda Akil[160], Fan Meng[160,92], Anne T. Spijker[161], Szabolcs Szelinger[162], Aiden Corvin[101], Michael Gill[101], Janice M. Fullerton[163,164], Peter R. Schofield[163,164], Claudio Toma[163,164], Qingqin S. Li[165], Eline J. Regeer[166], Niamh Mullins[167], Stéphane Jamain[168,111], Alan F. Schatzberg[169], Vishwajit Nimgaonkar[170], Janet L. Sobell[171], Ralph Kupka[172,173,174], Adebayo Anjorin[175], Roy H. Perlis[176,177], John I. Nurnberger[178], Jacob Lawrence[179], Helmut Vedder[180], Judith A. Badner[181], Marco P. Boks[88], Roel A. Ophoff[88,120,7], William Byerley[182], Chunyu Liu[183], Wade H. Berrettini[184], Allan H. Young[185], Katherine Gordon-Smith[186], Amy Perry[186], Lisa A. Jones[186], Jennifer M. Whitehead Pavlides[187], Naomi R. Wray[187,123], Sigrid Børte[188,189,190], Susan L. McElroy[191], William A. Scheftner[192], Elaine K. Green[193], Melissa J. Green[194,163], Philip B. Mitchell[194], Cynthia S. Weickert[194,163,195], Thomas W. Weickert[194], Nicholas J. Schork[196], Radhika Kandaswamy[197], Peter McGuffin[197,163,195], Simone de Jong[197,198], Margarita Rivera[197,199], Saskia P. Hagenaars[197,198], Cathryn M. Lewis[197,198,200], Liam Abbott[148], Richard Belliveau[148], Felecia Cerrato[148], Kimberly Chambert[148], Danfeng Chen[148],

Ashley Dumont[148], Diane Gage[148], Jaqueline Goldstein[148], Claire Churchhouse[148,149], Steve McCarroll[148,150], Mark J. Daly[148,149], Stephan Ripke[148,78,149], Phil H. Lee[148,149,201], Jordan W. Smoller[148,202,203], David W. Craig[204], William Coryell[205], Eva Reininghaus[206], Susanne Bengesser[206], NIna Dalkner[206], Armin Birner[206], Panagiotis Ferentinos[207,208], Kristi Krebs[209], Christine Lochner[210], Dan J. Stein[211], Nathan McGregor[212], Bendik S. Winsvold[213,190], Ole K. Drange[155], Ben M. Brumpton[190], Kristian Hveem[190,214], John-Anker Zwart[213,190,125]

[1]Department of Biomedical and NeuroMotor Sciences, University of Bologna, Bologna, IT. [2]Department of Biomedicine, University of Basel, Basel, CH. [3]Department of Psychiatry (UPK), University of Basel, Basel, CH. [4]Institute of Human Genetics, University of Bonn, School of Medicine & University Hospital Bonn, Bonn, DE. [5]Centre for Human Genetics, University of Marburg, Marburg, DE. [6]Institute of Medical Genetics and Pathology, University Hospital Basel, Basel, CH. [7]Center for Neurobehavioral Genetics, University of California Los Angeles, Los Angeles, CA, US. [8]Biostatistics, University of Minnesota System, Minneapolis, MN, US. [9]Department of Clinical Sciences, Psychiatry, Umeå University Medical Faculty, Umeå, SE. [10]Department of Psychiatry and Addiction Medicine, Assistance Publique - Hôpitaux de Paris, Paris, FR. [11]UMR-S1144 Team 1: Biomarkers of relapse and therapeutic response in addiction and mood disorders, INSERM, Paris, FR. [12]Psychiatry, Université Paris Diderot, Paris, FR. [13]Centre for Affective Disorders, Institute of Psychiatry, Psychology and Neuroscience, London, GB. [14]Department of Medical Epidemiology and Biostatistics, Karolinska Institutet, Stockholm, SE. [15]Department of Pathology and Laboratory Medicine, University of California Los Angeles, Los Angeles, CA, US. [16]Centre for Psychiatry, Queen Mary University of London, London, GB. [17]UCL Genetics Institute, University College London, London, GB. [18]Center for Statistical Genetics and Department of Biostatistics, University of Michigan, Ann Arbor, MI, US. [19]Department of Neuroscience, Istituto Di Ricerche Farmacologiche Mario Negri IRCCS, Milano, IT. [20]Clinical Institute of Neuroscience, Hospital Clinic, University of Barcelona, IDIBAPS, CIBERSAM, Barcelona, ES. [21]Department of Genetics and Genomic Sciences, Icahn School of Medicine at Mount Sinai, New York, NY, US. [22]Department of Psychiatry, Icahn School of Medicine at Mount Sinai, New York, NY, US. [23]Medical and Population Genetics, Broad Institute, Cambridge, MA, US. [24]Department of Psychiatric Research, Diakonhjemmet Hospital, Oslo, NO. [25]Department of Health Sciences Research, Mayo Clinic, Rochester, MN, US. [26]Department of Genetic Epidemiology in Psychiatry, Central Institute of Mental Health, Medical Faculty Mannheim, Heidelberg University, Mannheim, DE. [27]deCODE Genetics / Amgen, Reykjavik, IS. [28]Paris Bipolar and TRD Expert Centres, FondaMental Foundation, Paris, FR. [29]Department of Neurology and Neurosurgery, McGill University, Faculty of Medicine, Montreal, QC, CA. [30]Montreal Neurological Institute and Hospital, Montreal, QC, CA. [31]Biochemistry and Molecular Biology, Indiana University School of Medicine, Indianapolis, IN, US. [32]Department of Clinical Neuroscience, Centre for Psychiatry Research, Karolinska Institutet, Stockholm, SE. [33]NORMENT, KG Jebsen Centre for Psychosis Research, Division of Mental Health and Addiction, Institute of Clinical Medicine and Diakonhjemmet Hospital, University of Oslo, Oslo, NO. [34]Cell Biology, SUNY Downstate Medical Center College of Medicine, Brooklyn, NY, US. [35]Institute for Genomic Health, SUNY Downstate Medical Center College of Medicine, Brooklyn, NY, US. [36]Campbell Family Mental Health Research Institute, Centre for Addiction and Mental Health, Toronto, ON, CA. [37]Neurogenetics Section, Centre for Addiction and Mental Health, Toronto, ON, CA. [38]Department of Psychiatry, University of Toronto, Toronto, ON, CA. [39]Institute of Medical Sciences, University of Toronto, Toronto, ON, CA. [40]Centre for Addiction and Mental Health, Toronto, ON, CA. [41]Department of Biostatistics, Princess Margaret Cancer Centre, Toronto, ON, CA. [42]Dalla Lana School of Public Health, University

of Toronto, Toronto, ON, CA. [43]Cancer Epidemiology and Prevention, M. Sklodowska-Curie Cancer Center and Institute of Oncology, Warsaw, PL. [44]Applied Molecular Genomics Unit, VIB Department of Molecular Genetics, University of Antwerp, Antwerp, Belgium. [45]Faculty of Medicine, University of Iceland, Reykjavik, IS. [46]Department of Cognitive Science, University of California San Diego, La Jolla, CA, US. [47]Department of Complex Trait Genetics, Center for Neurogenomics and Cognitive Research, Amsterdam Neuroscience, Vrije Universiteit Amsterdam, Amsterdam, NL. [48]Department of Clinical Genetics, Amsterdam Neuroscience, Vrije Universiteit Medical Center, Amsterdam, NL. [49]Department of Clinical Psychiatry, Psychiatry Clinic, Clinical Center University of Sarajevo, Sarajevo, BA. [50]Biometric Psychiatric Genetics Research Unit, Alexandru Obregia Clinical Psychiatric Hospital, Bucharest, RO. [51]Department of Psychiatry & Psychology, Mayo Clinic, Rochester, MN, US. [52]Clinic for Psychiatry and Psychotherapy, University Hospital Cologne, Cologne, DE. [53]Institute of Neuroscience and Physiology, University of Gothenburg, Gothenburg, SE. [54]Department of Neurosciences, University of California San Diego, La Jolla, CA, US. [55]Div Mental Health and Addiction, Oslo University Hospital, Oslo, NO. [56]NORMENT, University of Oslo, Oslo, NO. [57]Department of Human Genetics, University of Michigan, Ann Arbor, MI, US. [58]Department of Medical & Molecular Genetics, Indiana University, Indianapolis, IN, US. [59]Department of Neuroscience, Icahn School of Medicine at Mount Sinai, New York, NY, US. [60]Department of Mental Health, Johns Hopkins University Bloomberg School of Public Health, Baltimore, MD, US. [61]Department of Genetics, University of North Carolina at Chapel Hill, Chapel Hill, NC, US. [62]Department of Psychiatry, University of North Carolina at Chapel Hill, Chapel Hill, NC, US. [63]Computational Sciences Center of Emphasis, Pfizer Global Research and Development, Cambridge, MA, US. [64]Department of Medical Genetics, Oslo University Hospital Ullevål, Oslo, NO. [65]NORMENT, KG Jebsen Centre for Psychosis Research, Department of Clinical Science, University of Bergen, Bergen, NO. [66]Center for Multimodal Imaging and Genetics, University of California San Diego, La Jolla, CA, US. [67]Department of Radiology, University of California San Diego, La Jolla, CA, US. [68]Department of Psychiatry, University of California San Diego, La Jolla, CA, US. [69]Institute of Neuroscience and Medicine (INM-1), Research Centre Jülich, Jülich, DE. [70]Department of Neurology, Oslo University Hospital, Oslo, NO. [71]NORMENT, KG Jebsen Centre for Psychosis Research, Oslo University Hospital, Oslo, NO. [72]Department of Psychiatry and Behavioral Neuroscience, University of Chicago, Chicago, IL, US. [73]Department of Human Genetics, University of Chicago, Chicago, IL, US. [74]Department of Psychiatry and Behavioral Sciences, Howard University Hospital, Washington, DC, US. [75]Department of Psychiatry and Behavioral Sciences, Johns Hopkins University School of Medicine, Baltimore, MD, US. [76]Psychiatry, Brigham and Women's Hospital, Boston, MA, US. [77]Department of Psychiatry and Human Behavior, University of California, Irvine, Irvine, CA, US. [78]Department of Psychiatry and Psychotherapy, Charité - Universitätsmedizin, Berlin, DE. [79]Department of Psychiatry and Psychotherapy, University Hospital Carl Gustav Carus, Technische Universität Dresden, Dresden, DE. [80]Department of Psychiatry and Psychotherapy, University of Bonn, Bonn, DE. [81]Department of Psychiatry, Dalhousie University, Halifax, NS, CA. [82]National Institute of Mental Health, Klecany, CZ. [83]Department of Psychiatry, Hospital Namsos, Namsos, NO. [84]Department of Neuroscience, Norges Teknisk Naturvitenskapelige Universitet Fakultet for naturvitenskap og teknologi, Trondheim, NO. [85]Department of Psychiatry, Laboratory of Psychiatric Genetics, Poznan University of Medical Sciences, Poznan, PL. [86]Department of Psychiatry, McGill University, Montreal, QC, CA. [87]Department of Psychiatry, Mood Disorders Program, McGill University Health Center, Montreal, QC, CA. [88]Psychiatry, UMC Utrecht Hersencentrum Rudolf Magnus, Utrecht, NL. [89]Department of Translational Research in Psychiatry, Max Planck Institute of Psychiatry, Munich, DE. [90]Department of Psychiatry, Psychosomatic Medicine

and Psychotherapy, University Hospital Frankfurt, Frankfurt am Main, DE. [91]Research/Psychiatry, Veterans Affairs San Diego Healthcare System, San Diego, CA, US. [92]Department of Psychiatry, University of Michigan, Ann Arbor, MI, US. [93]Department of Psychiatry, University of Münster, Münster, DE. [94]Department of Psychiatry, Washington University in Saint Louis, Saint Louis, MO, US. [95]Department of Psychiatry, Weill Cornell Medical College, New York, NY, US. [96]Department of Psychology, Eberhard Karls Universität Tübingen, Tubingen, DE. [97]Munich Cluster for Systems Neurology (SyNergy), Munich, DE. [98]University of Liverpool, Liverpool, GB. [99]Dept of Psychiatry, Sankt Olavs Hospital Universitetssykehuset i Trondheim, Trondheim, NO. [100]Discipline of Biochemistry, Neuroimaging and Cognitive Genomics (NICOG) Centre, National University of Ireland, Galway, Galway, IE. [101]Neuropsychiatric Genetics Research Group, Dept of Psychiatry and Trinity Translational Medicine Institute, Trinity College Dublin, Dublin, IE. [102]Division of Mental Health and Addiction, Oslo University Hospital, Oslo, NO. [103]Division of Mental Health and Addiction, University of Oslo, Institute of Clinical Medicine, Oslo, NO. [104]Division of Psychiatry, Haukeland Universitetssjukehus, Bergen, NO. [105]Faculty of Medicine and Dentistry, University of Bergen, Bergen, NO. [106]Division of Psychiatry, University College London, London, GB. [107]Division of Psychiatry, University of Edinburgh, Edinburgh, GB. [108]Centre for Cognitive Ageing and Cognitive Epidemiology, University of Edinburgh, Edinburgh, GB. [109]Estonian Genome Center, University of Tartu, Tartu, EE. [110]Institute of Molecular and Cell Biology, University of Tartu, Tartu, EE. [111]Faculté de Médecine, Université Paris Est, Créteil, FR. [112]INSERM, Paris, FR. [113]Faculty of Medicine, Department of Psychiatry, School of Health Sciences, University of Iceland, Reykjavik, IS. [114]Genetic Cancer Susceptibility Group, International Agency for Research on Cancer, Lyon, FR. [115]Genetics and Computational Biology, QIMR Berghofer Medical Research Institute, Brisbane, QLD, AU. [116]School of Psychology, The University of Queensland, Brisbane, QLD, AU. [117]HudsonAlpha Institute for Biotechnology, Huntsville, AL, US. [118]Human Genetics and Computational Biomedicine, Pfizer Global Research and Development, Groton, CT, US. [119]Human Genetics Branch, Intramural Research Program, National Institute of Mental Health, Bethesda, MD, US. [120]Human Genetics, University of California Los Angeles, Los Angeles, CA, US. [121]College of Medicine Institute for Genomic Health, SUNY Downstate Medical Center College of Medicine, Brooklyn, NY, US. [122]Institute for Medical Sciences, University of Aberdeen, Aberdeen, UK. [123]Institute for Molecular Bioscience, The University of Queensland, Brisbane, QLD, AU. [124]Institute of Biological Psychiatry, Mental Health Centre Sct. Hans, Copenhagen, DK. [125]Institute of Clinical Medicine, University of Oslo, Oslo, NO. [126]Institute of Genetic Medicine, Johns Hopkins University School of Medicine, Baltimore, MD, US. [127]Institute of Psychiatric Phenomics and Genomics (IPPG), University Hospital, LMU Munich, Munich, DE. [128]Department of Psychiatry and Psychotherapy, University Medical Center Göttingen, Göttingen, DE. [129]Instituto de Salud Carlos III, Biomedical Network Research Centre on Mental Health (CIBERSAM), Madrid, ES. [130]Department of Psychiatry, Hospital Universitari Vall d´Hebron, Barcelona, ES. [131]Psychiatric Genetics Unit, Group of Psychiatry Mental Health and Addictions, Vall d´Hebron Research Institut (VHIR), Universitat Autònoma de Barcelona, Barcelona, ES. [132]Department of Psychiatry and Forensic Medicine, Universitat Autònoma de Barcelona, Barcelona, ES. [133]iPSYCH, The Lundbeck Foundation Initiative for Integrative Psychiatric Research, DK. [134]Center for Neonatal Screening, Department for Congenital Disorders, Statens Serum Institut, Copenhagen, DK. [135]Psychosis Research Unit, Aarhus University Hospital, Risskov, DK. [136]Mental Health Services in the Capital Region of Denmark, Mental Health Center Copenhagen, University of Copenhagen, Copenhagen, DK. [137]National Centre for Register-Based Research, Aarhus University, Aarhus, DK. [138]Centre for Integrated Register-based Research, Aarhus University, Aarhus, DK. [139]Institute of Biological

Psychiatry, MHC Sct. Hans, Mental Health Services Copenhagen, Roskilde, DK. [140]Department of Clinical Medicine, University of Copenhagen, Copenhagen, DK. [141]iSEQ, Center for Integrative Sequencing, Aarhus University, Aarhus, DK. [142]Department of Biomedicine - Human Genetics, Aarhus University, Aarhus, DK. [143]Bioinformatics Research Centre, Aarhus University, Aarhus, DK. [144]Department of Psychiatry, Psychosomatics and Psychotherapy, Center of Mental Health, University Hospital Würzburg, Würzburg, DE. [145]ISGlobal, Barcelona, ES. [146]Max Planck Institute of Psychiatry, Munich, DE. [147]Division of Endocrinology and Center for Basic and Translational Obesity Research, Boston Children's Hospital, Boston, MA, US. [148]Stanley Center for Psychiatric Research, Broad Institute, Cambridge, MA, US. [149]Analytic and Translational Genetics Unit, Massachusetts General Hospital, Boston, MA, US. [150]Department of Genetics, Harvard Medical School, Boston, MA, US. [151]Division of Endocrinology, Children's Hospital Boston, Boston, MA, US. [152]Medical Research Council Centre for Neuropsychiatric Genetics and Genomics, Division of Psychological Medicine and Clinical Neurosciences, Cardiff University, Cardiff, GB. [153]Medicine, Psychiatry, Biomedical Informatics, Vanderbilt University Medical Center, Nashville, TN, US. [154]Mental Health Department, University Regional Hospital, Biomedicine Institute (IBIMA), Málaga, ES. [155]Mental Health, Faculty of Medicine and Health Sciences, Norwegian University of Science and Technology - NTNU, Trondheim, NO. [156]Psychiatry, St Olavs University Hospital, Trondheim, NO. [157]Mental Health, NHS 24, Glasgow, GB. [158]Division of Psychiatry, Centre for Clinical Brain Sciences, University of Edinburgh, Edinburgh, GB. [159]Molecular & Behavioral Neuroscience Institute and Department of Computational Medicine & Bioinformatics, University of Michigan, Ann Arbor, MI, US. [160]Molecular & Behavioral Neuroscience Institute, University of Michigan, Ann Arbor, MI, US. [161]Mood Disorders, PsyQ, Rotterdam, NL. [162]Neurogenomics, TGen, Los Angeles, AZ, US. [163]Neuroscience Research Australia, Sydney, NSW, AU. [164]School of Medical Sciences, University of New South Wales, Sydney, NSW, AU. [165]Neuroscience Therapeutic Area, Janssen Research and Development, LLC, Titusville, NJ, US. [166]Outpatient Clinic for Bipolar Disorder, Altrecht, Utrecht, NL. [167]Pamela Sklar Division of Psychiatric Genomics, Department of Genetics and Genomic Sciences, Icahn School of Medicine at Mount Sinai, New York, US.. [168]Psychiatrie Translationnelle, Inserm U955, Créteil, FR. [169]Psychiatry and Behavioral Sciences, Stanford University School of Medicine, Stanford, CA, US. [170]Psychiatry and Human Genetics, University of Pittsburgh, Pittsburgh, PA, US. [171]Psychiatry and the Behavioral Sciences, University of Southern California, Los Angeles, CA, US. [172]Psychiatry, Altrecht, Utrecht, NL. [173]Psychiatry, GGZ inGeest, Amsterdam, NL. [174]Psychiatry, VU medisch centrum, Amsterdam, NL. [175]Psychiatry, Berkshire Healthcare NHS Foundation Trust, Bracknell, GB. [176]Psychiatry, Harvard Medical School, Boston, MA, US. [177]Division of Clinical Research, Massachusetts General Hospital, Boston, MA, US. [178]Psychiatry, Indiana University School of Medicine, Indianapolis, IN, US. [179]Psychiatry, North East London NHS Foundation Trust, Ilford, GB. [180]Psychiatry, Psychiatrisches Zentrum Nordbaden, Wiesloch, DE. [181]Psychiatry, Rush University Medical Center, Chicago, IL, US. [182]Psychiatry, University of California San Francisco, San Francisco, CA, US. [183]Psychiatry, University of Illinois at Chicago College of Medicine, Chicago, IL, US. [184]Psychiatry, University of Pennsylvania, Philadelphia, PA, US. [185]Psychological Medicine, Institute of Psychiatry, Psychology & Neuroscience, King's College London, London, GB. [186]Psychological Medicine, University of Worcester, Worcester, GB. [187]Queensland Brain Institute, The University of Queensland, Brisbane, QLD, AU. [188]Research and Communication Unit for Musculoskeletal Health, Division of Clinical Neuroscience, Oslo University Hospital, Ullevål, Oslo, Norway. [189]Institute of Clinical Medicine, Faculty of Medicine, University of Oslo, Oslo, Norway. [190]K. G. Jebsen Center for Genetic Epidemiology, Department of Public Health and Nursing, Faculty of Medicine and Health

Sciences, Norwegian University of Science and Technology, Trondheim, Norway. [191]Research Institute, Lindner Center of HOPE, Mason, OH, US. [192]Rush University Medical Center, Chicago, IL, US. [193]School of Biomedical and Healthcare Sciences, Plymouth University Peninsula Schools of Medicine and Dentistry, Plymouth, GB. [194]School of Psychiatry, University of New South Wales, Sydney, NSW, AU. [195]Department of Neuroscience, SUNY Upstate Medical University, Syracuse, NY USA. [196]Scripps Translational Science Institute, La Jolla, CA, US. [197]Social, Genetic and Developmental Psychiatry Centre, King's College London, London, GB. [198]NIHR Maudsley BRC, King's College London, London, GB. [199]Department of Biochemistry and Molecular Biology II, Institute of Neurosciences, Center for Biomedical Research, University of Granada, Granada, ES. [200]Department of Medical & Molecular Genetics, King's College London, London, GB. [201]Psychiatric and Neurodevelopmental Genetics Unit, Massachusetts General Hospital, Boston, MA, US. [202]Department of Psychiatry, Massachusetts General Hospital, Boston, MA, US. [203]Psychiatric and Neurodevelopmental Genetics Unit (PNGU), Massachusetts General Hospital, Boston, MA, US. [204]Translational Genomics, USC, Phoenix, AZ, US. [205]University of Iowa Hospitals and Clinics, Iowa City, IA, US. [206]Medical University of Graz, Department of Psychiatry and Psychotherapeutic Medicine. [207]National and Kapodistrian University of Athens, 2nd Department of Psychiatry, Attikon General Hospital, Athens, Greece. [208]Social, Genetic and Developmental Psychiatry Centre, King's College London, London, UK. [209]Estonian Genome Center, Institute of Genomics, University of Tartu, Tartu, Estonia. [210]SAMRC Unit on Risk & Resilience in Mental Disorders, Dept of Psychiatry, Stellenbosch University. [211]SAMRC Unit on Risk & Resilience in Mental Disorders, Dept of Psychiatry & Neuroscience Institute, University of Cape Town. [212]SAMRC Unit on Risk & Resiliience in Mental Disorders, Dept of Psychiatry, Stellenbosch University. [213]Department of Research, Innovation and Education, Division of Clinical Neuroscience, Oslo University Hospital, Oslo, Norway. [214]HUNT Research Center, Department of Public Health and Nursing, Faculty of Medicine and Health Sciences, Norwegian University of Science and Technology, Trondheim, Norway

Eating Disorders Working Group of the Psychiatric Genomics Consortium

Melissa A. Munn-Chernoff[1], Laura M. Thornton[1], Zeynep Yilmaz[1,2], Jessica H. Baker[1], Scott Gordon[3], Sarah E. Medland[3], Hunna J. Watson[1,4,5], Julien Bryois[6], Anke Hinney[7], Virpi M. Leppä[6], Manuel Mattheisen[8,9,10,11], Stephan Ripke[12,13,14], Shuyang Yao[6], Paola Giusti-Rodríguez[2], Roger A. Adan[15,16,17], Lars Alfredsson[18], Tetsuya Ando[19], Ole A. Andreassen[20], Wade H. Berrettini[21], Ilka Boehm[22], Claudette Boni[23], Vesna Boraska Perica[24,25], Katharina Buehren[26], Roland Burghardt[27], Matteo Cassina[28], Sven Cichon[29,30,31], Maurizio Clementi[28], Roger D. Cone[32], Philippe Courtet[33], Scott Crow[34], James J. Crowley[2,9], Unna N. Danner[35], Oliver S. Davis[36,37], Martina de Zwaan[38], George Dedoussis[39], Daniela Degortes[40], Janiece E. DeSocio[41], Danielle M. Dick[42,43,44], Dimitris Dikeos[45], Christian Dina[46], Monika Dmitrzak-Weglarz[47], Elisa Docampo[48,49,50], Laramie E. Duncan[51], Karin Egberts[52], Stefan Ehrlich[22], Geòrgia Escaramís[48,49,50], Tõnu Esko[53,54], Xavier Estivill[48,49,50,55], Anne Farmer[56], Angela Favaro[40], Fernando Fernández-Aranda[57,58], Manfred M. Fichter[59,60], Krista Fischer[53], Manuel Föcker[61], Lenka Foretova[62], Andreas J. Forstner[63,64,65,30], Monica Forzan[28], Christopher S. Franklin[24], Steven Gallinger[66], Ina Giegling[67], Johanna Giuranna[68], Fragiskos Gonidakis[69], Philip Gorwood[70,71], Monica Gratacos Mayora[48,49,50], Sébastien Guillaume[33], Yiran Guo[72], Hakon Hakonarson[72,73], Konstantinos Hatzikotoulas[74,24], Joanna Hauser[75], Johannes Hebebrand[7], Sietske G. Helder[56,76], Stefan Herms[29,30], Beate Herpertz-Dahlmann[26], Wolfgang Herzog[77], Laura M. Huckins[24,78], James I. Hudson[79], Hartmut Imgart[80], Hidetoshi Inoko[81], Vladimir Janout[82], Susana Jiménez-Murcia[57,58], Antonio Julià[83], Gursharan Kalsi[56], Deborah Kaminská[84], Leila Karhunen[85], Andreas Karwautz[86], Martien J. Kas[15,87], James L.

Kennedy[88,89,90], Anna Keski-Rahkonen[91], Kirsty Kiezebrink[92], Youl-Ri Kim[93], Kelly L. Klump[94], Gun Peggy S. Knudsen[95], Maria C. La Via[1], Stephanie Le Hellard[96,97,98], Robert D. Levitan[89], Dong Li[72], Lisa Lilenfeld[99], Bochao Danae Lin[15], Jolanta Lissowska[100], Jurjen Luykx[15], Pierre J. Magistretti[101,102], Mario Maj[103], Katrin Mannik[53,104], Sara Marsal[83], Christian R. Marshall[105], Morten Mattingsdal[106], Sara McDevitt[107,108], Peter McGuffin[56], Andres Metspalu[53,109], Ingrid Meulenbelt[110], Nadia Micali[111,112], Karen Mitchell[113,114], Alessio Maria Monteleone[103], Palmiero Monteleone[115], Benedetta Nacmias[116], Marie Navratilova[62], Ioanna Ntalla[39], Julie K. O'Toole[117], Roel A. Ophoff[118,119], Leonid Padyukov[120], Aarno Palotie[54,121,122], Jacques Pantel[23], Hana Papezova[84], Dalila Pinto[78], Raquel Rabionet[123,124,125], Anu Raevuori[91], Nicolas Ramoz[23], Ted Reichborn-Kjennerud[95,126], Valdo Ricca[127], Samuli Ripatti[128], Franziska Ritschel[22,129], Marion Roberts[56], Alessandro Rotondo[130], Dan Rujescu[67], Filip Rybakowski[131], Paolo Santonastaso[132], André Scherag[133], Stephen W. Scherer[134,135], Ulrike Schmidt[136], Nicholas J. Schork[137], Alexandra Schosser[138], Jochen Seitz[26], Lenka Slachtova[139], P. Eline Slagboom[140], Margarita C. Slof-Op 't Landt[141,142], Agnieszka Slopien[143], Sandro Sorbi[116,144], Beata Świątkowska[145], Jin P. Szatkiewicz[2], Ioanna Tachmazidou[24], Elena Tenconi[40], Alfonso Tortorella[146,147], Federica Tozzi[148], Janet Treasure[136], Artemis Tsitsika[149], Marta Tyszkiewicz-Nwafor[143], Konstantinos Tziouvas[150], Annemarie A. van Elburg[16,151], Eric F. van Furth[141,142], Gudrun Wagner[86], Esther Walton[22], Elisabeth Widen[121], Eleftheria Zeggini[74,24], Stephanie Zerwas[1], Stephan Zipfel[152], Andrew W. Bergen[153,154], Joseph M. Boden[155], Harry Brandt[156], Steven Crawford[156], Katherine A. Halmi[157], L. John Horwood[155], Craig Johnson[158], Allan S. Kaplan[88,89,90], Walter H. Kaye[159], James Mitchell[160], Catherine M. Olsen[161], John F. Pearson[162], Nancy L. Pedersen[6], Michael Strober[163,164], Thomas Werge[165], David C. Whiteman[161], D. Blake Woodside[89,90,166,167], Jakob Grove[8,168,169,170], Anjali K. Henders[171], Janne T. Larsen[168,172,173], Richard Parker[3], Liselotte V. Petersen[168,172,173], Jennifer Jordan[174,175], Martin A. Kennedy[176], Andreas Birgegård[9,10,6], Paul Lichtenstein[6], Claes Norring[9,10], Mikael Landén[6,177], Preben Bo Mortensen[168,172,173], Tracey D. Wade[178], Grant W. Montgomery[3,171,179], Nicholas G. Martin[3], Patrick F. Sullivan[1,2,6], Jaakko Kaprio[91,121]

[1]Department of Psychiatry, University of North Carolina at Chapel Hill, Chapel Hill, North Carolina, USA. [2]Department of Genetics, University of North Carolina at Chapel Hill, Chapel Hill, North Carolina, USA. [3]QIMR Berghofer Medical Research Institute, Brisbane, Queensland, Australia. [4]School of Psychology, Curtin University, Perth, Western Australia, Australia. [5]School of Paediatrics and Child Health, University of Western Australia, Perth, Western Australia, Australia. [6]Department of Medical Epidemiology and Biostatistics, Karolinska Institutet, Stockholm, Sweden. [7]Department of Child and Adolescent Psychiatry, University Hospital Essen, University of Duisburg-Essen, Essen, Germany. [8]Department of Biomedicine, Aarhus University, Aarhus, Denmark. [9]Department of Clinical Neuroscience, Karolinska Institutet, Stockholm, Sweden. [10]Center for Psychiatry Research, Stockholm Health Care Services, Stockholm City Council, Stockholm, Sweden. [11]Department of Psychiatry, Psychosomatics and Psychotherapy, University of Würzburg, Würzburg, Germany. [12]Analytic and Translational Genetics Unit, Department of Medicine, Massachusetts General Hospital and Harvard Medical School, Boston, Massachusetts, USA. [13]Stanley Center for Psychiatric Research, Broad Institute of the Massachusetts Institute of Technology and Harvard University, Cambridge, Massachusetts, USA. [14]Department of Psychiatry and Psychotherapy, Charité - Universitätsmedizin, Berlin, Germany. [15]Brain Center Rudolf Magnus, Department of Translational Neuroscience, University Medical Center Utrecht, Utrecht, The Netherlands. [16]Center for Eating Disorders Rintveld, Altrecht Mental Health Institute, Zeist, The Netherlands. [17]Sahlgrenska Academy, University of Gothenburg, Gothenburg, Sweden. [18]Institute of Environmental Medicine, Karolinska

Institutet, Stockholm, Sweden. [19]Department of Behavioral Medicine, National Institute of Mental Health, National Center of Neurology and Psychiatry, Kodaira, Tokyo, Japan. [20]NORMENT Centre, Division of Mental Health and Addiction, University of Oslo, Oslo University Hospital, Oslo, Norway. [21]Department of Psychiatry, Center for Neurobiology and Behavior, University of Pennsylvania Perelman School of Medicine, Philadelphia, Pennsylvania, USA. [22]Division of Psychological and Social Medicine and Developmental Neurosciences, Faculty of Medicine, Technische Universität Dresden, Dresden, Germany. [23]INSERM U894, Centre of Psychiatry and Neuroscience, Paris, France. [24]Wellcome Sanger Institute, Wellcome Genome Campus, Hinxton, Cambridge, UK. [25]Department of Medical Biology, School of Medicine, University of Split, Split, Croatia. [26]Department of Child and Adolescent Psychiatry, Psychosomatics and Psychotherapy, RWTH Aachen University, Aachen, Germany. [27]Klinikum Frankfurt/Oder, Frankfurt, Germany. [28]Clinical Genetics Unit, Department of Woman and Child Health, University of Padova, Padova, Italy. [29]Institute of Medical Genetics and Pathology, University Hospital Basel, Basel, Switzerland. [30]Department of Biomedicine, University of Basel, Basel, Switzerland. [31]Institute of Neuroscience and Medicine (INM-1), Research Center Juelich, Juelich, Germany. [32]Life Sciences Institute and Department of Molecular and Integrative Physiology, University of Michigan, Ann Arbor, Michigan, USA. [33]Department of Emergency Psychiatry and Post-Acute Care, CHRU Montpellier, University of Montpellier, Montpellier, France. [34]Department of Psychiatry, University of Minnesota, Minneapolis, Minnesota, USA. [35]Altrecht Eating Disorders Rintveld, Altrecht Mental Health Institute, Zeist, The Netherlands. [36]MRC Integrative Epidemiology Unit, University of Bristol, Bristol, UK. [37]School of Social and Community Medicine, University of Bristol, Bristol, UK. [38]Department of Psychosomatic Medicine and Psychotherapy, Hannover Medical School, Hannover, Germany. [39]Department of Nutrition and Dietetics, Harokopio University, Athens, Greece. [40]Department of Neurosciences, University of Padova, Padova, Italy. [41]College of Nursing, Seattle University, Seattle, Washington, USA. [42]Department of Psychology, Virginia Commonwealth University, Richmond, Virginia, USA. [43]College Behavioral and Emotional Health Institute, Virginia Commonwealth University, Richmond, Virginia, USA. [44]Department of Human & Molecular Genetics, Virginia Commonwealth University, Richmond, Virginia, USA. [45]Department of Psychiatry, Athens University Medical School, Athens University, Athens, Greece. [46]l'institut du thorax, INSERM, CNRS, Univ Nantes, Nantes, France. [47]Department of Psychiatric Genetics, Poznan University of Medical Sciences, Poznan, Poland. [48]Barcelona Institute of Science and Technology, Barcelona, Spain. [49]Universitat Pompeu Fabra, Barcelona, Spain. [50]Centro de Investigación Biomédica en Red en Epidemiología y Salud Pública (CIBERESP), Barcelona, Spain. [51]Department of Psychiatry and Behavioral Sciences, Stanford University, Stanford, California, USA. [52]Department of Child and Adolescent Psychiatry, Psychosomatics and Psychotherapy, University Hospital of Würzburg, Centre for Mental Health, Würzburg, Germany. [53]Estonian Genome Center, University of Tartu, Tartu, Estonia. [54]Program in Medical and Population Genetics, Broad Institute of the Massachusetts Institute of Technology and Harvard University, Cambridge, Massachusetts, USA. [55]Genomics and Disease, Bioinformatics and Genomics Programme, Centre for Genomic Regulation, Barcelona, Spain. [56]Institute of Psychiatry, Psychology and Neuroscience, Social, Genetic and Developmental Psychiatry (SGDP) Centre, King's College London, London, UK. [57]Department of Psychiatry, University Hospital of Bellvitge –IDIBELL and CIBERobn, Barcelona, Spain. [58]Department of Clinical Sciences, School of Medicine, University of Barcelona, Barcelona, Spain.

[59]Department of Psychiatry and Psychotherapy, Ludwig-Maximilians-University, Munich, Germany. [60]Schön Klinik Roseneck affiliated with the Medical Faculty of the University of Munich, Munich, Germany. [61]Department of Child and Adolescent Psychiatry, University of

Münster, Münster, Germany. [62]Department of Cancer, Epidemiology and Genetics, Masaryk Memorial Cancer Institute, Brno, Czech Republic. [63]Centre for Human Genetics, University of Marburg, Marburg, Germany. [64]Institute of Human Genetics, University of Bonn, School of Medicine & University Hospital Bonn, Bonn, Germany. [65]Department of Psychiatry (UPK), University of Basel, Basel, Switzerland. [66]Department of Surgery, Faculty of Medicine, University of Toronto, Toronto, Ontario, Canada. [67]Department of Psychiatry, Psychotherapy and Psychosomatics, Martin-Luther-University Halle-Wittenberg, Halle (Saale), Germany. [68]Department of Child and Adolescent Psychiatry, University Hospital Essen, University of Duisburg-Essen, Essen, Germany. [69]1st Psychiatric Department, National and Kapodistrian University of Athens, Medical School, Eginition Hospital, Athens, Greece. [70]INSERM U1266, Institute of Psychiatry and Neuroscience of Paris, Paris, France. [71]CMME (GHU Paris Psychiatrie et Neurosciences), Paris Descartes University, Paris, France. [72]Center for Applied Genomics, Children's Hospital of Philadelphia, Philadelphia, Pennsylvania, USA. [73]Department of Pediatrics, University of Pennsylvania Perelman School of Medicine, Philadelphia, Pennsylvania, USA. [74]Institute of Translational Genomics, Helmholtz Zentrum München - German Research Centre for Environmental Health, Neuherberg, Germany. [75]Department of Adult Psychiatry, Poznan University of Medical Sciences, Poznan, Poland. [76]Zorg op Orde, Delft, The Netherlands. [77]Department of General Internal Medicine and Psychosomatics, Heidelberg University Hospital, Heidelberg University, Heidelberg, Germany. [78]Department of Psychiatry, and Genetics and Genomics Sciences, Division of Psychiatric Genomics, Icahn School of Medicine at Mount Sinai, New York, New York, USA. [79]Biological Psychiatry Laboratory, McLean Hospital/Harvard Medical School, Boston, Massachusetts, USA. [80]Eating Disorders Unit, Parklandklinik, Bad Wildungen, Germany. [81]Department of Molecular Life Science, Division of Basic Medical Science and Molecular Medicine, School of Medicine, Tokai University, Isehara, Japan. [82]Faculty of Health Sciences, Palacky University, Olomouc, Czech Republic. [83]Rheumatology Research Group, Vall d'Hebron Research Institute, Barcelona, Spain. [84]Department of Psychiatry, First Faculty of Medicine, Charles University, Prague, Czech Republic. [85]Institute of Public Health and Clinical Nutrition, Department of Clinical Nutrition, University of Eastern Finland, Kuopio, Finland. [86]Eating Disorders Unit, Department of Child and Adolescent Psychiatry, Medical University of Vienna, Vienna, Austria. [87]Groningen Institute for Evolutionary Life Sciences, University of Groningen, Groningen, The Netherlands. [88]Centre for Addiction and Mental Health, Toronto, Ontario, Canada. [89]Institute of Medical Science, University of Toronto, Toronto, Ontario, Canada. [90]Department of Psychiatry, University of Toronto, Toronto, Ontario, Canada. [91]Department of Public Health, University of Helsinki, Helsinki, Finland. [92]Institute of Applied Health Sciences, School of Medicine, Medical Sciences and Nutrition, University of Aberdeen, Aberdeen, UK. [93]Department of Psychiatry, Seoul Paik Hospital, Inje University, Seoul, Korea. [94]Department of Psychology, Michigan State University, East Lansing, Michigan, USA. [95]Department of Mental Disorders, Norwegian Institute of Public Health, Oslo, Norway. [96]Department of Clinical Science, Norwegian Centre for Mental Disorders Research (NORMENT), University of Bergen, Bergen, Norway. [97]Dr. Einar Martens Research Group for Biological Psychiatry, Center for Medical Genetics and Molecular Medicine, Haukeland University Hospital, Bergen, Norway. [98]Department of Clinical Medicine, Laboratory Building, Haukeland University Hospital, Bergen, Norway. [99]The Chicago School of Professional Psychology, Washington DC Campus, USA. [100]Department of Cancer Epidemiology and Prevention, M Skłodowska-Curie Cancer Center - Oncology Center, Warsaw, Poland. [101]BESE Division, King Abdullah University of Science and Technology, Thuwal, Saudi Arabia. [102]Department of Psychiatry, University of Lausanne-University Hospital of Lausanne (UNIL-CHUV), Lausanne, Switzerland. [103]Department of Psychiatry,

University of Campania "Luigi Vanvitelli", Naples, Italy. [104]Center for Integrative Genomics, University of Lausanne, Lausanne, Switzerland. [105]Department of Paediatric Laboratory Medicine, Division of Genome Diagnostics, The Hospital for Sick Children, Toronto, Ontario, Canada. [106]NORMENT KG Jebsen Centre, Division of Mental Health and Addiction, University of Oslo, Oslo University Hospital, Oslo, Norway. [107]Department of Psychiatry, University College Cork, Cork, Ireland. [108]Eist Linn Adolescent Unit, Bessborough, Health Service Executive South, Cork, Ireland. [109]Institute of Molecular and Cell Biology, University of Tartu, Tartu, Estonia. [110]Molecular Epidemiology Section (Department of Biomedical Datasciences), Leiden University Medical Centre, Leiden, The Netherlands. [111]Department of Psychiatry, Faculty of Medicine, University of Geneva, Geneva, Switzerland. [112]Division of Child and Adolescent Psychiatry, Geneva University Hospital, Geneva, Switzerland. [113]National Center for PTSD, VA Boston Healthcare System, Boston, Massachusetts, USA. [114]Department of Psychiatry, Boston University School of Medicine, Boston, Massachusetts, USA. [115]Department of Medicine, Surgery and Dentistry "Scuola Medica Salernitana", University of Salerno, Salerno, Italy. [116]Department of Neuroscience, Psychology, Drug Research and Child Health (NEUROFARBA), University of Florence, Florence, Italy. [117]Kartini Clinic, Portland, Oregon, USA. [118]Center for Neurobehavioral Genetics, Semel Institute for Neuroscience and Human Behavior, University of California Los Angeles, Los Angeles, California, USA. [119]Department of Psychiatry, Erasmus MC, University Medical Center Rotterdam, Rotterdam, The Netherlands. [120]Division of Rheumatology, Department of Medicine, Center for Molecular Medicine, Karolinska Institutet and Karolinska University Hospital, Stockholm, Sweden. [121]Institute for Molecular Medicine FIMM, HiLIFE, University of Helsinki, Helsinki, Finland. [122]Center for Human Genome Research, Massachusetts General Hospital, Boston, Massachusetts, USA. [123]Saint Joan de Déu Research Institute, Saint Joan de Déu Barcelona Children's Hospital, Barcelona, Spain. [124]Institute of Biomedicine (IBUB), University of Barcelona, Barcelona, Spain. [125]Department of Genetics, Microbiology and Statistics, University of Barcelona, Barcelona, Spain. [126]Institute of Clinical Medicine, University of Oslo, Oslo, Norway. [127]Department of Health Science, University of Florence, Florence, Italy. [128]Department of Biometry, University of Helsinki, Helsinki, Finland. [129]Eating Disorders Research and Treatment Center, Department of Child and Adolescent Psychiatry, Faculty of Medicine, Technische Universität Dresden, Dresden, Germany. [130]Department of Psychiatry, Neurobiology, Pharmacology, and Biotechnologies, University of Pisa, Pisa, Italy. [131]Department of Psychiatry, Poznan University of Medical Sciences, Poznan, Poland. [132]Department of Neurosciences, Padua Neuroscience Center, University of Padova, Padova, Italy. [133]Institute of Medical Statistics, Computer and Data Sciences, Jena University Hospital, Jena, Germany. [134]Department of Genetics and Genomic Biology, The Hospital for Sick Children, Toronto, Ontario, Canada. [135]McLaughlin Centre, University of Toronto, Toronto, Ontario, Canada. [136]Institute of Psychiatry, Psychology and Neuroscience, Department of Psychological Medicine, King's College London, London, UK. [137]J. Craig Venter Institute (JCVI), La Jolla, California, USA. [138]Department of Psychiatry and Psychotherapy, Medical University of Vienna, Vienna, Austria. [139]Department of Pediatrics and Center of Applied Genomics, First Faculty of Medicine, Charles University, Prague, Czech Republic. [140]Molecular Epidemiology Section (Department of Medical Statistics), Leiden University Medical Centre, Leiden, The Netherlands. [141]Center for Eating Disorders Ursula, Rivierduinen, Leiden, The Netherlands. [142]Department of Psychiatry, Leiden University Medical Centre, Leiden, The Netherlands. [143]Department of Child and Adolescent Psychiatry, Poznan University of Medical Sciences, Poznan, Poland. [144]IRCCS Fondazione Don Carlo Gnocchi, Florence, Italy. [145]Department of Environmental Epidemiology, Nofer Institute of Occupational Medicine, Lodz, Poland. [146]Department of Psychiatry, University of

Naples SUN, Naples, Italy. [147]Department of Psychiatry, University of Perugia, Perugia, Italy. [148]Brain Sciences Department, Stremble Ventures, Limassol, Cyprus. [149]Adolescent Health Unit, Second Department of Pediatrics, "P. & A. Kyriakou" Children's Hospital, University of Athens, Athens, Greece. [150]Pediatric Intensive Care Unit, "P. & A. Kyriakou" Children's Hospital, University of Athens, Athens, Greece. [151]Faculty of Social and Behavioral Sciences, Utrecht University, Utrecht, The Netherlands. [152]Department of Internal Medicine VI, Psychosomatic Medicine and Psychotherapy, University Medical Hospital Tuebingen, Tuebingen, Germany. [153]BioRealm, LLC, Walnut, California, USA. [154]Oregon Research Institute, Eugene, Oregon, USA. [155]Christchurch Health and Development Study, University of Otago, Christchurch, New Zealand. [156]The Center for Eating Disorders at Sheppard Pratt, Baltimore, Maryland, USA. [157]Department of Psychiatry, Weill Cornell Medical College, New York, New York, USA. [158]Eating Recovery Center, Denver, Colorado, USA. [159]Department of Psychiatry, University of California San Diego, La Jolla, California, USA. [160]Department of Psychiatry and Behavioral Science, University of North Dakota School of Medicine and Health Sciences, Fargo, North Dakota, USA. [161]Population Health Department, QIMR Berghofer Medical Research Institute, Brisbane, Queensland, Australia. [162]Biostatistics and Computational Biology Unit, University of Otago, Christchurch, New Zealand. [163]Department of Psychiatry and Biobehavioral Science, Semel Institute for Neuroscience and Human Behavior, University of California Los Angeles, Los Angeles, California, USA. [164]David Geffen School of Medicine, University of California Los Angeles, Los Angeles, California, USA. [165]Department of Clinical Medicine, University of Copenhagen, Copenhagen, Denmark. [166]Centre for Mental Health, University Health Network, Toronto, Ontario, Canada. [167]Program for Eating Disorders, University Health Network, Toronto, Ontario, Canada. [168]The Lundbeck Foundation Initiative for Integrative Psychiatric Research (iPSYCH), Aarhus, Denmark. [169]Centre for Integrative Sequencing, iSEQ, Aarhus University, Aarhus, Denmark. [170]Bioinformatics Research Centre, Aarhus University, Aarhus, Denmark. [171]Institute for Molecular Bioscience, University of Queensland, Brisbane, Queensland, Australia. [172]National Centre for Register-Based Research, Aarhus BSS, Aarhus University, Aarhus, Denmark. [173]Centre for Integrated Register-based Research (CIRRAU), Aarhus University, Aarhus, Denmark. [174]Department of Psychological Medicine, University of Otago, Christchurch, New Zealand. [175]Canterbury District Health Board, Christchurch, New Zealand. [176]Department of Pathology and Biomedical Science, University of Otago, Christchurch, New Zealand. [177]Department of Psychiatry and Neurochemistry, Institute of Neuroscience and Physiology, The Sahlgrenska Academy at the University of Gothenburg, Gothenburg, Sweden. [178]School of Psychology, Flinders University, Adelaide, South Australia, Australia. [179]Queensland Brain Institute, University of Queensland, Brisbane, Queensland, Australia

Major Depressive Disorder Working Group of the Psychiatric Genomics Consortium

Naomi R. Wray[1,2], Stephan Ripke[3,4,5], Manuel Mattheisen[6,7,8], Maciej Trzaskowski[1], Enda M. Byrne[1], Abdel Abdellaoui[9], Mark J. Adams[10], Esben Agerbo[11,12,13], Tracy M. Air[14], Till F. Andlauer[15,16], Silviu-Alin Bacanu[17], Marie Bækvad-Hansen[18,13], Aartjan T. Beekman[19], Tim B. Bigdeli[17,20], Elisabeth B. Binder[21,15], Julien Bryois[22], Henriette N. Buttenschøn[23,24,13], Jonas Bybjerg-Grauholm[18,13], Na Cai[25,26], Enrique Castelao[27], Jane Hvarregaard Christensen[8,24,13], Toni-Kim Clarke[10], Lucía Colodro-Conde[28], Baptiste Couvy-Duchesne[29,2], Nick Craddock[30], Gregory E. Crawford[31,32], Gail Davies[33], Ian J. Deary[33], Franziska Degenhardt[34], Eske M. Derks[28], Nese Direk[35,36], Conor V. Dolan[9], Erin C. Dunn[37,38,39], Thalia C. Eley[40], Valentina Escott-Price[41], Farnush F. Kiadeh[42], Hilary K. Finucane[43,44], Jerome C. Foo[45], Andreas J. Forstner[46,47,34,48], Josef Frank[45], Michael Gill[49], Fernando S. Goes[50], Scott D. Gordon[28], Jakob Grove[51,8,24,13], Lynsey S. Hall[10,52,18], Christine Søholm Hansen[13], Thomas

F. Hansen[53,54,55], Stefan Herms[47,34], Ian B. Hickie[56], Per Hoffmann[47,34], Georg Homuth[57], Carsten Horn[58], Jouke-Jan Hottenga[9], David M. Hougaard[18,13], David M. Howard[10,40], Marcus Ising[59], Rick Jansen[19], Ian Jones[60], Lisa A. Jones[61], Eric Jorgenson[62], James A. Knowles[63], Isaac S. Kohane[64,65,66], Julia Kraft[4], Warren W. Kretzschmar[67], Zoltán Kutalik[68,69], Yihan Li[67], Penelope A. Lind[28], Donald J. MacIntyre[70,71], Dean F. MacKinnon[50], Robert M. Maier[2], Wolfgang Maier[72], Jonathan Marchini[73], Hamdi Mbarek[9], Patrick McGrath[74], Peter McGuffin[40], Sarah E. Medland[28], Divya Mehta[2,75], Christel M. Middeldorp[76,77,9], Evelin Mihailov[78], Yuri Milaneschi[19], Lili Milani[78], Francis M. Mondimore[50], Grant W. Montgomery[1], Sara Mostafavi[79,80], Niamh Mullins[40], Matthias Nauck[81,82], Bernard Ng[80], Michel G. Nivard[9], Dale R. Nyholt[83], Hogni Oskarsson[84], Michael J. Owen[60], Jodie N. Painter[28], Carsten B. Pedersen[11,12,13], Marianne G. Pedersen[11,12,13], Roseann E. Peterson[17,85], Erik Pettersson[22], Wouter J. Peyrot[19], Giorgio Pistis[27], Danielle Posthuma[86,87], Jorge A. Quiroz[88], Per Qvist[8,24,13], John P. Rice[89], Brien P. Riley[17], Margarita Rivera[90,40], Saira S. Mirza[35], Robert Schoevers[91], Eva C. Schulte[92,93], Ling Shen[62], Jianxin Shi[94], Stanley I. Shyn[95], Engilbert Sigurdsson[96], Grant C. Sinnamon[97], Johannes H. Smit[19], Daniel J. Smith[98], Hreinn Stefansson[99], Stacy Steinberg[99], Fabian Streit[45], Jana Strohmaier[45], Katherine E. Tansey[100], Henning Teismann[101], Alexander Teumer[102], Wesley Thompson[103,54,104,13], Pippa A. Thomson[105], Thorgeir E. Thorgeirsson[99], Matthew Traylor[106], Jens Treutlein[45], Vassily Trubetskoy[4], André G. Uitterlinden[107], Daniel Umbricht[108], Sandra van der Auwera[109], Albert M. van Hemert[110], Alexander Viktorin[22], Peter M. Visscher[1,2], Yunpeng Wang[54,104,13], Bradley T. Webb[85], Shantel M. Weinsheimer[54,13], Jürgen Wellmann[101], Gonneke Willemsen[9], Stephanie H. Witt[45], Yang Wu[1], Hualin S. Xi[111], Jian Yang[112,2], Futao Zhang[1], Volker Arolt[113], Bernhard T. Baune[114,115,116], Klaus Berger[101], Dorret I. Boomsma[9], Sven Cichon[47,34,117,118], Udo Dannlowski[113], EJC de Geus[119,9], J Raymond DePaulo[50], Enrico Domenici[120], Katharina Domschke[121,122], Tõnu Esko[78,5], Hans J. Grabe[109], Steven P. Hamilton[123], Caroline Hayward[124], Andrew C. Heath[89], Kenneth S. Kendler[17], Stefan Kloiber[125,126,59], Glyn Lewis[127], Qingqin S. Li[128], Susanne Lucae[59], Pamela A. Madden[89], Patrik K. Magnusson[22], Nicholas G. Martin[28], Andrew M. McIntosh[33,10], Andres Metspalu[78,129], Ole Mors[130,13], Preben B. Mortensen[11,24,12,13], Bertram Müller-Myhsok[15,131,132], Merete Nordentoft[133,13], Markus M. Nöthen[34], Michael C. O'Donovan[60], Sara A. Paciga[134], Nancy L. Pedersen[22], Brenda W. Penninx[19], Roy H. Perlis[37,135], David J. Porteous[105], James B. Potash[136], Martin Preisig[27], Marcella Rietschel[45], Catherine Schaefer[62], Thomas G. Schulze[45,137,138,139,93], Jordan W. Smoller[37,38,39], Kari Stefansson[140,99], Henning Tiemeier[141,35,142], Rudolf Uher[143], Henry Völzke[102], Myrna M. Weissman[144,74], Thomas Werge[145,54,13], Cathryn M. Lewis[146,40], Douglas F. Levinson[147], Anders D. Børglum[8,24,13], Patrick F. Sullivan[22,148,149]

[1]Institute for Molecular Bioscience, The University of Queensland, Brisbane, QLD, AU. [2]Queensland Brain Institute, The University of Queensland, Brisbane, QLD, AU. [3]Analytic and Translational Genetics Unit, Massachusetts General Hospital, Boston, MA, US. [4]Department of Psychiatry and Psychotherapy, Universitätsmedizin Berlin Campus Charité Mitte, Berlin, DE. [5]Medical and Population Genetics, Broad Institute, Cambridge, MA, US. [6]Department of Psychiatry, Psychosomatics and Psychotherapy,, University of Wurzburg, Wurzburg, DE. [7]Centre for Psychiatry Research, Department of Clinical Neuroscience, Karolinska Institutet, Stockholm, SE. [8]Department of Biomedicine, Aarhus University, Aarhus, DK. [9]Dept of Biological Psychology & EMGO+ Institute for Health and Care Research, Vrije Universiteit Amsterdam, Amsterdam, NL. [10]Division of Psychiatry, University of Edinburgh, Edinburgh, GB. [11]Centre for Integrated Register-based Research, Aarhus University, Aarhus, DK. [12]National Centre for Register-Based Research, Aarhus University, Aarhus, DK. [13]iPSYCH, The Lundbeck Foundation Initiative for Integrative

Psychiatric Research, DK. [14]Discipline of Psychiatry, University of Adelaide, Adelaide, SA, AU. [15]Department of Translational Research in Psychiatry, Max Planck Institute of Psychiatry, Munich, DE. [16]Department of Neurology, Klinikum rechts der Isar,, Technical University of Munich, Munich, DE. [17]Department of Psychiatry, Virginia Commonwealth University, Richmond, VA, US. [18]Center for Neonatal Screening, Department for Congenital Disorders, Statens Serum Institut, Copenhagen, DK. [19]Department of Psychiatry, Vrije Universiteit Medical Center and GGZ inGeest, Amsterdam, NL. [20]Virginia Institute for Psychiatric and Behavior Genetics, Richmond, VA, US. [21]Department of Psychiatry and Behavioral Sciences, Emory University School of Medicine, Atlanta, GA, US. [22]Department of Medical Epidemiology and Biostatistics, Karolinska Institutet, Stockholm, SE. [23]Department of Clinical Medicine, Translational Neuropsychiatry Unit, Aarhus University, Aarhus, DK. [24]iSEQ, Centre for Integrative Sequencing, Aarhus University, Aarhus, DK. [25]Human Genetics, Wellcome Trust Sanger Institute, Cambridge, GB. [26]Statistical genomics and systems genetics, European Bioinformatics Institute (EMBL-EBI), Cambridge, GB. [27]Department of Psychiatry, Lausanne University Hospital and University of Lausanne, Lausanne, CH. [28]Genetics and Computational Biology, QIMR Berghofer Medical Research Institute, Brisbane, QLD, AU. [29]Centre for Advanced Imaging, The University of Queensland, Brisbane, QLD, AU. [30]Psychological Medicine, Cardiff University, Cardiff, GB. [31]Center for Genomic and Computational Biology, Duke University, Durham, NC, US. [32]Department of Pediatrics, Division of Medical Genetics, Duke University, Durham, NC, US. [33]Centre for Cognitive Ageing and Cognitive Epidemiology, University of Edinburgh, Edinburgh, GB. [34]Institute of Human Genetics, University of Bonn, School of Medicine & University Hospital Bonn, Bonn, DE. [35]Epidemiology, Erasmus MC, Rotterdam, Zuid-Holland, NL. [36]Psychiatry, Dokuz Eylul University School Of Medicine, Izmir, TR. [37]Department of Psychiatry, Massachusetts General Hospital, Boston, MA, US. [38]Psychiatric and Neurodevelopmental Genetics Unit (PNGU), Massachusetts General Hospital, Boston, MA, US. [39]Stanley Center for Psychiatric Research, Broad Institute, Cambridge, MA, US. [40]Social, Genetic and Developmental Psychiatry Centre, King's College London, London, GB. [41]Neuroscience and Mental Health, Cardiff University, Cardiff, GB. [42]Bioinformatics, University of British Columbia, Vancouver, BC, CA. [43]Department of Epidemiology, Harvard T.H. Chan School of Public Health, Boston, MA, US. [44]Department of Mathematics, Massachusetts Institute of Technology, Cambridge, MA, US. [45]Department of Genetic Epidemiology in Psychiatry, Central Institute of Mental Health, Medical Faculty Mannheim, Heidelberg University, Mannheim, Baden-Württemberg, DE. [46]Department of Psychiatry (UPK), University of Basel, Basel, CH. [47]Department of Biomedicine, University of Basel, Basel, CH. [48]Centre for Human Genetics, University of Marburg, Marburg, DE. [49]Department of Psychiatry, Trinity College Dublin, Dublin, IE. [50]Psychiatry & Behavioral Sciences, Johns Hopkins University, Baltimore, MD, US. [51]Bioinformatics Research Centre, Aarhus University, Aarhus, DK. [52]Institute of Genetic Medicine, Newcastle University, Newcastle upon Tyne, GB. [53]Danish Headache Centre, Department of Neurology, Rigshospitalet, Glostrup, DK. [54]Institute of Biological Psychiatry, Mental Health Center Sct. Hans, Mental Health Services Capital Region of Denmark, Copenhagen, DK. [55]iPSYCH, The Lundbeck Foundation Initiative for Psychiatric Research, Copenhagen, DK. [56]Brain and Mind Centre, University of Sydney, Sydney, NSW, AU. [57]Interfaculty Institute for Genetics and Functional Genomics, Department of Functional Genomics, University Medicine and Ernst Moritz Arndt University Greifswald, Greifswald, Mecklenburg-Vorpommern, DE. [58]Roche Pharmaceutical Research and Early Development, Pharmaceutical Sciences, Roche Innovation Center Basel, F. Hoffmann-La Roche Ltd, Basel, CH. [59]Max Planck Institute of Psychiatry, Munich, DE. [60]MRC Centre for Neuropsychiatric Genetics and Genomics, Cardiff University, Cardiff, GB. [61]Department of Psychological Medicine, University of Worcester, Worcester, GB.

[62]Division of Research, Kaiser Permanente Northern California, Oakland, CA, US. [63]Psychiatry & The Behavioral Sciences, University of Southern California, Los Angeles, CA, US. [64]Department of Biomedical Informatics, Harvard Medical School, Boston, MA, US. [65]Department of Medicine, Brigham and Women's Hospital, Boston, MA, US. [66]Informatics Program, Boston Children's Hospital, Boston, MA, US. [67]Wellcome Trust Centre for Human Genetics, University of Oxford, Oxford, GB. [68]Institute of Social and Preventive Medicine (IUMSP), Lausanne University Hospital and University of Lausanne, Lausanne, VD, CH. [69]Swiss Institute of Bioinformatics, Lausanne, VD, CH. [70]Division of Psychiatry, Centre for Clinical Brain Sciences, University of Edinburgh, Edinburgh, GB. [71]Mental Health, NHS 24, Glasgow, GB. [72]Department of Psychiatry and Psychotherapy, University of Bonn, Bonn, DE. [73]Statistics, University of Oxford, Oxford, GB. [74]Psychiatry, Columbia University College of Physicians and Surgeons, New York, NY, US. [75]School of Psychology and Counseling, Queensland University of Technology, Brisbane, QLD, AU. [76]Child and Youth Mental Health Service, Children's Health Queensland Hospital and Health Service, South Brisbane, QLD, AU. [77]Child Health Research Centre, University of Queensland, Brisbane, QLD, AU. [78]Estonian Genome Center, University of Tartu, Tartu, EE. [79]Medical Genetics, University of British Columbia, Vancouver, BC, CA. [80]Statistics, University of British Columbia, Vancouver, BC, CA. [81]DZHK (German Centre for Cardiovascular Research), Partner Site Greifswald, University Medicine, University Medicine Greifswald, Greifswald, Mecklenburg-Vorpommern, DE. [82]Institute of Clinical Chemistry and Laboratory Medicine, University Medicine Greifswald, Greifswald, Mecklenburg-Vorpommern, DE. [83]Institute of Health and Biomedical Innovation, Queensland University of Technology, Brisbane, QLD, AU. [84]Humus, Reykjavik, IS. [85]Virginia Institute for Psychiatric & Behavioral Genetics, Virginia Commonwealth University, Richmond, VA, US. [86]Clinical Genetics, Vrije Universiteit Medical Center, Amsterdam, NL. [87]Complex Trait Genetics, Vrije Universiteit Amsterdam, Amsterdam, NL. [88]Solid Biosciences, Boston, MA, US. [89]Department of Psychiatry, Washington University in Saint Louis School of Medicine, Saint Louis, MO, US. [90]Department of Biochemistry and Molecular Biology II, Institute of Neurosciences, Biomedical Research Center (CIBM), University of Granada, Granada, ES. [91]Department of Psychiatry, University of Groningen, University Medical Center Groningen, Groningen, NL. [92]Department of Psychiatry and Psychotherapy, University Hospital, Ludwig Maximilian University Munich, Munich, DE. [93]Institute of Psychiatric Phenomics and Genomics (IPPG), University Hospital, Ludwig Maximilian University Munich, Munich, DE. [94]Division of Cancer Epidemiology and Genetics, National Cancer Institute, Bethesda, MD, US. [95]Behavioral Health Services, Kaiser Permanente Washington, Seattle, WA, US. [96]Faculty of Medicine, Department of Psychiatry, University of Iceland, Reykjavik, IS. [97]School of Medicine and Dentistry, James Cook University, Townsville, QLD, AU. [98]Institute of Health and Wellbeing, University of Glasgow, Glasgow, GB. [99]deCODE Genetics / Amgen, Reykjavik, IS. [100]College of Biomedical and Life Sciences, Cardiff University, Cardiff, GB. [101]Institute of Epidemiology and Social Medicine, University of Münster, Münster, Nordrhein-Westfalen, DE. [102]Institute for Community Medicine, University Medicine Greifswald, Greifswald, Mecklenburg-Vorpommern, DE. [103]Department of Psychiatry, University of California, San Diego, San Diego, CA, US. [104]KG Jebsen Centre for Psychosis Research, Norway Division of Mental Health and Addiction, Oslo University Hospital, Oslo, NO. [105]Medical Genetics Section, CGEM, IGMM, University of Edinburgh, Edinburgh, GB. [106]Clinical Neurosciences, University of Cambridge, Cambridge, GB. [107]Internal Medicine, Erasmus MC, Rotterdam, Zuid-Holland, NL. [108]Roche Pharmaceutical Research and Early Development, Neuroscience, Ophthalmology and Rare Diseases Discovery & Translational Medicine Area, Roche Innovation Center Basel, F. Hoffmann-La Roche Ltd, Basel, CH. [109]Department of

Psychiatry and Psychotherapy, University Medicine Greifswald, Greifswald, Mecklenburg-Vorpommern, DE. [110]Department of Psychiatry, Leiden University Medical Center, Leiden, NL. [111]Computational Sciences Center of Emphasis, Pfizer Global Research and Development, Cambridge, MA, US. [112]Institute for Molecular Bioscience; Queensland Brain Institute, The University of Queensland, Brisbane, QLD, AU. [113]Department of Psychiatry, University of Münster, Münster, Nordrhein-Westfalen, DE. [114]Department of Psychiatry, University of Münster, Münster, DE. [115]Department of Psychiatry, Melbourne Medical School, University of Melbourne, Melbourne, AU. [116]Florey Institute for Neuroscience and Mental Health,, University of Melbourne, Melbourne, AU. [117]Institute of Medical Genetics and Pathology, University Hospital Basel, University of Basel, Basel, CH. [118]Institute of Neuroscience and Medicine (INM-1), Research Center Juelich, Juelich, DE. [119]Amsterdam Public Health Institute, Vrije Universiteit Medical Center, Amsterdam, NL. [120]Centre for Integrative Biology, Università degli Studi di Trento, Trento, Trentino-Alto Adige, IT. [121]Department of Psychiatry and Psychotherapy, Medical Center - University of Freiburg,, Faculty of Medicine, University of Freiburg, Freiburg, DE. [122]Center for NeuroModulation, Faculty of Medicine, University of Freiburg, Freiburg, DE. [123]Psychiatry, Kaiser Permanente Northern California, San Francisco, CA, US. [124]Medical Research Council Human Genetics Unit, Institute of Genetics and Molecular Medicine, University of Edinburgh, Edinburgh, GB. [125]Department of Psychiatry, University of Toronto, Toronto, ON, CA. [126]Centre for Addiction and Mental Health, Toronto, ON, CA. [127]Division of Psychiatry, University College London, London, GB. [128]Neuroscience Therapeutic Area, Janssen Research and Development, LLC, Titusville, NJ, US. [129]Institute of Molecular and Cell Biology, University of Tartu, Tartu, EE. [130]Psychosis Research Unit, Aarhus University Hospital, Risskov, Aarhus, DK. [131]Munich Cluster for Systems Neurology (SyNergy), Munich, DE. [132]University of Liverpool, Liverpool, GB. [133]Mental Health Center Copenhagen, Copenhagen Universtity Hospital, Copenhagen, DK. [134]Human Genetics and Computational Biomedicine, Pfizer Global Research and Development, Groton, CT, US. [135]Psychiatry, Harvard Medical School, Boston, MA, US. [136]Psychiatry, University of Iowa, Iowa City, IA, US. [137]Department of Psychiatry and Behavioral Sciences, Johns Hopkins University, Baltimore, MD, US. [138]Department of Psychiatry and Psychotherapy, University Medical Center Göttingen, Goettingen, Niedersachsen, DE. [139]Human Genetics Branch, NIMH Division of Intramural Research Programs, Bethesda, MD, US. [140]Faculty of Medicine, University of Iceland, Reykjavik, IS. [141]Child and Adolescent Psychiatry, Erasmus MC, Rotterdam, Zuid-Holland, NL. [142]Psychiatry, Erasmus MC, Rotterdam, Zuid-Holland, NL. [143]Psychiatry, Dalhousie University, Halifax, NS, CA. [144]Division of Epidemiology, New York State Psychiatric Institute, New York, NY, US. [145]Department of Clinical Medicine, University of Copenhagen, Copenhagen, DK. [146]Department of Medical & Molecular Genetics, King's College London, London, GB. [147]Psychiatry & Behavioral Sciences, Stanford University, Stanford, CA, US. [148]Genetics, University of North Carolina at Chapel Hill, Chapel Hill, NC, US. [149]Psychiatry, University of North Carolina at Chapel Hill, Chapel Hill, NC, US

Obsessive Compulsive Disorder and Tourette Syndrome Working Group of the Psychiatric Genomics Consortium

Kelly Anderson[1], Paul D. Arnold[2,3], Kathleen D. Askland[4], Cristina Barlassina[5], Laura Bellodi[6], Katharina Bey[7], O. Joseph Bienvenu[8], Donald Black[9], Michael Bloch[10], Helena Brentani[11], Christie L. Burton[3], Beatriz Camarena[12], Carolina Cappi[13], Danielle Cath[14], Maria Cavallini[15], Valentina Ciullo[16], David Conti[17], Edwin Cook[18], Vladimir Coric[19], Nancy Cox[20], James J. Crowley[21,22], Bernadette A. Cullen[8], Danielle Cusi[23], Lea K. Davis[20,24], Richard Delorme[25,26], Damiaan Denys[27,28], Eske Derks[29], Valsamma Eapen[30,31], Christopher

Edlund[32], Lauren Erdman[3], Peter Falkai[33], Abigail J Fyer[34], Daniel A. Geller[35], Fernando S. Goes[8], Hans Grabe[36], Marco A. Grados[8], Benjamin D. Greenberg[4, 37, 38], Dorothy Grice[39], Edna Grünblatt[40,41,42,43], Wei Guo[44], Matthew Halvorson[45], Gregory L. Hanna[46], Ana G. Hounie[11], Michael Jenike[35], Clare Keenan[47], James Kennedy[48,49,50,51], Ekaterina A. Khramtsova[24], James A. Knowles[32, 52], Cristoph Lange[53], Nuria Lanzagorta[54], Marion Leboyer[25,55,56], Bingbin Li[57], Kung-Yee Liang[58], Christine Lochner[59], Jurjen Luykx[60,61], Fabio Macciardi[62], Brion Maher[8], Carol A. Mathews[63], Manuel Mattheisen[21,40,64,65], James T. McCracken[66], Nathaniel McGregor[67], Nicole C. McLaughlin[4], Euripedes C. Miguel[11], Rainald Moessner[68], Benjamin Neale[35,69] , Gerald Nestadt[8], Paul S. Nestadt[8,39], Humberto Nicolini[54,70] , Erika Nurmi[66], Lisa Osiecki[35], Michele Pato[32], Carlos Pato[32], David L. Pauls[71], John Piacentini[32], Fabrizio Piras[72,73], Federica Piras[72], Christopher Pittenger[74], Danielle Posthuma[75], Ann E. Pulver[8], Steven A. Rasmussen[4], Scott Rauch[76], Margaret A. Richter[49, 50, 51], Mark A. Riddle[8], Stephan Ripke[69,77,78], Maria Rosário[11], David Rosenberg[79], Stephan Ruhrmann[80], Aline S. Sampaio[81], Jack F. Samuels[8], Jeremiah M Scharf[35, 69, 82], Yin Yao Shugart[83], Jan Smit[75], Dirk J. A. Smit[28, 75], Gianfranco Spalletta[72,84] , Dan J. Stein[85], S Evelyn Stewart[86], Eric Storch[84], Barbara Stranger[87], Maurizio Turiel[88], Homero Vallada[13], Jeremy Veenstra-VanderWeele[34,89], Karin Verweij[90], Nienke Vulink[28], Michael Wagner[7], Susanne Walitza[41, 42], Ying Wang[8], Jens Wendland[91], Dongmei Yu[35, 69], Gwyneth Zai[49, 51]

[1]Epidemiology & Biostatistics, Western University, Ontario, Canada, [2]Mental Health Research and Education, University of Calgary Cumming School of Medicine, Calgary, Canada, [3]Genetics and Genome Biology, Hospital for Sick Children, Toronto, Canada, [4]Psychiatry & Human Behavior, Butler Hospital, Brown University, Providence, RI, USA, [5]Dept. of Health Science, University of Milan, Milan, Italy, [6]Psychology faculty, Università Vita-Salute San Raffaele, Milan, Italy, [7]Klinik und Poliklinik für Psychiatrie und Psychotherapie, University of Bonn, Bonn, Nordrhein-Westfalen, Germany, [8]Psychiatry, Johns Hopkins University, Baltimore, MD, USA, [9]Psychiatry, University of Iowa Roy J and Lucille A Carver College of Medicine, Iowa City, IA, USA, [10]Child Study Center, Yale School of Medicine, New Haven CT, USA, [11]Institute of Psychiatry, University of São Paulo, São Paulo, Brazil, [12]Psychiatric genetics, Instituto Nacional de Psiquiatría Ramon de la Fuente, Mexico City, Mexico, [13]Psychiatry, University of São Paulo, School of Medicine: Sao Paulo, Spain, [14]Social and Behavioural Sciences, Utrecht University, Utrecht, Netherlands, [15]Center for Eating Disorders, Ospedale San Raffaele: Milan, Italy, [16]Psychiatry, Università degli Studi di Roma La Sapienza, Rome, Italy, [17]Center for Genetic Epidemiology, Keck School of Medicine, University of Southern California, Los Angeles, CA, USA, [18]Psychiatry, University of Illinois at Chicago College of Medicine, Chicago, IL, USA, [19]Psychiatry, Yale School of Medicine, New Haven, CT, USA, [20]Genetics Institute, Vanderbilt University Medical Center, Nashville, TN, USA, [21]Dept. of Clinical Neuroscience, Karolinska Institutet, Stockholm, Sweden, [22]Dept. of Genetics and Psychiatry, University of North Carolina, Chapel Hill, NC, USA, [23]Institute of Biomedical Technologies, Italian National Research Council, Milan, Italy, [24]Genetic Medicine, University of Chicago, Chicago, IL, USA, [25]Psychiatry Department, Assistance Publique - Hôpitaux de Paris, Paris, France, [26]Medical School, Université Paris Diderot, Paris, France, [27]Neuromodulation & Behavior, Netherlands Institute for Neuroscience: Amsterdam, Holland, [28]Dept. of Psychiatry, Academic Medical Center, Amsterdam, Holland, [29]Translational Neurogenomics, QIMR Berghofer Medical Research Institute: Herston, Australia, [30]Dept. of Psychiatry, University of New South Wales, Sydney, Australia, [31]Child Psychiatry, South Western Sydney Local Health District & Ingham Institute, Liverpool, Australia, [32]Dept. of Psychiatry, Keck School of Medicine, University of Southern California, Los Angeles, CA, USA, [33]Dept. of Psychiatry and Psychotherapy, Ludwig-

Maximilians-Universitat Munchen, München, Germany, [34]Department of Psychiatry, Columbia University, New York, NY, USA, [35]Dept. of Psychiatry, Massachusetts General Hospital, Boston, MA, USA, [36]Dept. of Psychiatry and Psychotherapy, University Medicine Greifswald, Greifswald, Germany, [37]Psychiatry and Human Behavior, Brown University Warren Alpert Medical School, Providence, RI, USA, [38]Center for Neurorestoration and Neurotechnology, Providence VA Medical Center, Providence, RI, USA, [39]Dept. of Psychiatry, Icahn School of Medicine at Mount Sinai, New York, NY, USA, [40]Dept. of Psychiatry, Psychosomatic and Psychotherapy, University Hospital of Würzburg, Würzburg, Germany, [41]Dept. of Child and Adolescent Psychiatry and Psychotherapy, Psychiatric Hospital, University of Zurich, Zurich, Germany, [42]Neuroscience Center Zurich, University of Zurich and ETH Zurich, Zurich, Germany, [43]Zurich Center for Integrative Human Physiology, University of Zurich, Zurich, Germany, [44]Statistical Genomics and Data Analysis Core, National Institute of Mental Health, Bethesda, MD, USA, [45]Dept. of Psychiatry, University of North Carolina at Chapel Hill School of Medicine, Chapel Hill, NC, USA, [46]Dept. of Psychiatry, University of Michigan, Ann Arbor, MI, USA, [47]Massachusetts Institute of Technology, Cambridge, MA, USA, [48]Campbell Family Mental Health Research Institute, Centre for Addiction and Mental Health: Toronto, Canada, [49]Neurogenetics, Centre for Addiction and Mental Health: Toronto, Canada, [50]Institute of Medical Sciences, University of Toronto: Toronto, Canada, [51]Dept. of Psychiatry, University of Toronto, Toronto, Canada, [52]Cell Biology, SUNY Downstate Medical Center College of Medicine, Brooklyn, NY, USA, [53]Dept. of Biostatistics, Harvard T.H. Chan, School of Public Health, Boston, MA, USA, [54]Group of Medical and Family Studies Carracci, Mexico City, Mexico, [55]Psychiatry, INSERM, Paris, France, [56]Psychiatry, Université Paris-Est Créteil Val de Marne Faculté de médecine: Creteil, France, [57]Rapid7, Toronto, Canada, [58]National Health Research Institute, Taipei, Taiwan, [59]Psychiatry, Stellenbosch University, Stellenbosch, South Africa, [60]Psychiatry, Utrecht University, Utrecht, Netherlands, [61]Translational Neuroscience, Utrecht University, Utrecht, Netherlands, [62]Psychiatry & Human Behavior, University of California, Irvine UCI, Irvine, CA, USA, [63]Dept. of Psychiatry and UF Genetics Institute, University of Florida, Gainesville, FL, USA, [64]Biomedicine, Aarhus University, Aarhus, Denmark, [65]Health Care Services, Stockholm County Council, Stockholm, Germany, [66]Psychiatry and Biobehavioral Sciences, Brain Research Institute, University of California Los Angeles UCLA, Los Angeles, CA, USA, [67]Genetics, Stellenbosch University, Stellenbosch, South Africa, [68]Dept. of Psychiatry and Psychotherapy, University of Tübingen, Tübingen, Germany, [69]Psychiatric Research, Broad Institute, Cambridge, MA, USA, [70]Instituto Nacional de Medicina Genómica, Arenal Tepepan, Mexico, [71]Dept. of Psychiatry, Harvard Medical School, Boston, MA, USA, [72]Dept. of Clinical and Behavioral Neurology, IRCCS Santa Lucia Foundation, Rome, Italy, [73]Center for Studies and Research, Enrico Fermi Center for Study and Research, Rome, Italy, [74]Dept. of Psychiatry, Yale University School of Medicine, New Haven, CT, USA, [75]Clinical Genetics, Vrije University, Amsterdam, Holland, [76]Dept. of Psychiatry, McLean Harvard Medical School Affliate, Belmont, MA, USA, [77]Analytic and Translational Genetics Unit, Massachusetts General Hospital, Boston, MA, USA, [78]Dept. of Psychiatry, Charité – Universitätsmedizin, Berlin, Germany, [79]Dept. of Psychiatry, School of Medicine, Wayne State University, Detroit, MI, USA, [80]Dept. of Psychiatry, University of Cologne, Bonn, Germany, [81]Dept. of Psychiatry, Federal University of Bahia, Salvador, Brazil, [82]Dept. of Neurology, Brigham and Women's Hospital, Boston, MA, USA, [83]National Institute of Health, Bethesda, MD, USA, [84]Dept. of Psychiatry, Baylor College of Medicine, Houston, TX, USA, [85]Dept. of Psychiatry, University of Cape Town, Cape Town, South Africa, [86]Dept. of Psychiatry, University of Ottawa, Ottawa, Canada, [87]Institute for Genomics and Systems Biology, University of Chicago, Chicago, IL, USA, [88]Orthopedic Institute IRCCS, University of Milan, Milan, Italy, [89]Dept. of Psychiatry, New

York Psychiatric Institute, New York, NY, USA, [90]Dept. of Psychiatry, University of Amsterdam, Amsterdam, Holland, [91]Pfizer Inc., Pharma Therapeutics Clinical Research, New York, NY, USA

Post-Traumatic Stress Disorder Working Group of the Psychiatric Genomics Consortium

Laramie E. Duncan[1,2,3], Andrew Ratanatharathorn[4], Allison E. Aiello[5], Lynn M. Almli[6], Ananda B. Amstadter[7], Allison E. Ashley-Koch[8], Dewleen G. Baker[9,10], Jean C. Beckham[11,12], Laura J. Bierut[13], Jonathan Bisson[14], Bekh Bradley[15,16], Chia-Yen Chen[3,17,18], Shareefa Dalvie[19], Lindsay A. Farrer[20], Sandro Galea[21], Melanie E. Garrett[8], Joel E. Gelernter[22], Guia Guffanti[18,23], Michael A. Hauser[8], Eric O. Johnson[24], Ronald C. Kessler[25], Nathan A. Kimbrel[11,12], Anthony King[26], Nastassja Koen[27,28], Henry R. Kranzler[29], Mark W. Logue[30,31], Adam X. Maihofer[32], Alicia R. Martin[2,3], Mark W. Miller[30,33], Rajendra A. Morey[12,34], Nicole R. Nugent[35,36], John P. Rice[37], Stephan Ripke[2,3,38], Andrea L. Roberts[39], Nancy L. Saccone[40], Jordan W. Smoller[2,17], Dan J. Stein[27,28], Murray B. Stein[32,41,42], Jennifer A. Sumner[43], Monica Uddin[44], Robert J. Ursano[45], Derek E. Wildman[46], Rachel Yehuda[47,48], Hongyu Zhao[49], Mark J. Daly[2,3], Israel Liberzon[26,50], Kerry J. Ressler[18,23], Caroline M. Nievergelt[9,10] and Karestan C. Koenen[2,17,51]

[1]Department of Psychiatry, Stanford University, Stanford, CA, USA. [2]Broad Institute of MIT and Harvard, Stanley Center for Psychiatric Research, Boston, MA, USA. [3]The Analytic and Translational Genetics Unit, Massachusetts General Hospital, Boston, MA, USA. [4]Department of Epidemiology, Columbia University, New York, NY, USA. [5]Department of Epidemiology, Gillings School of Global Public Health, University of North Carolina, Chapel Hill, Chapel Hill, NC, USA. [6]Department of Psychiatry and Behavioral Sciences, Emory University, Atlanta, GA, USA. [7]Department of Psychiatry, Virginia Commonwealth University, Richmond, VA, USA. [8]Department of Medicine, Duke Molecular Physiology Institute, Duke University Medical Center, Durham, NC, USA. [9]Veterans Affairs San Diego Healthcare System and Veterans Affairs Center of Excellence for Stress and Mental Health, San Diego, CA, USA. [10]Department of Psychiatry, University of California, San Diego, San Diego, CA, USA. [11]Veterans Affairs Durham Healthcare System, Durham, NC, USA. [12]Department of Psychiatry and Behavioral Sciences, Duke University Medical Center, Durham, NC, USA. [13]Department of Psychiatry, Washington University School of Medicine, St Louis, MO, USA. [14]Division of Psychological Medicine and Clinical Neurosciences, Cardiff University, Cardiff, UK. [15]Atlanta VA Medical Center, Atlanta, GA, USA. [16]Department of Psychiatry, Emory University, Atlanta, GA, USA. [17]Psychiatric and Neurodevelopmental Genetics Unit, Center for Human Genetic Research, and Department of Psychiatry, Massachusetts General Hospital, Boston, MA, USA. [18]Department of Psychiatry, Harvard University, Cambridge, MA, USA. [19]Division of Human Genetics, University of Cape Town, Cape Town, South Africa. [20]Biomedical Genetics, Boston University School of Medicine, Boston, MA, USA. [21]Boston University School of Public Health, Boston, MA, USA. [22]Department of Psychiatry, Yale University School of Medicine and VA CT Healthcare System, New Haven, CT, USA. [23]Department of Psychiatry, McLean Hospital, Belmont, MA, USA. [24]RTI International, Research Triangle Park, NC, USA. [25]Department of Health Care Policy, Harvard Medical School, Boston, MA, USA. [26]Department of Psychiatry, University of Michigan, Ann Arbor, MI, USA. [27]Department of Psychiatry and Mental Health, University of Cape Town, Cape Town, South Africa. [28]MRC Unit on Anxiety & Stress Disorders, Groote Schuur Hospital, Cape Town, South Africa. [29]Department of Psychiatry, University of Pennsylvania Perelman School of Medicine and VISN 4 MIRECC, Crescenz VAMC, Philadelphia, PA, USA. [30]VA Boston Healthcare System, Jamaica Plain,

MA, USA. [31]Department of Medicine, Boston University School of Medicine, Boston, MA, USA. [32]Department of Psychiatry, University of California, San Diego, La Jolla, CA, USA. [33]Department of Psychiatry, Boston University School of Medicine, Boston, MA, USA. [34]Durham VA Medical Center, Durham, NC, USA. [35]Division of Behavioral Genetics, Department of Psychiatry, Rhode Island Hospital, Providence, RI, USA. [36]Department of Psychiatry and Human Behavior, Alpert Medical School of Brown University, Providence, RI, USA. [37]Department of Psychiatry, Washington University, St Louis, MO, USA. [38]Department of Psychiatry and Psychotherapy, Charité, Campus Mitte, Berlin, Germany. [39]Department of Social and Behavioral Sciences, Harvard T. H. Chan School of Public Health Cambridge, MA, USA. [40]Department of Genetics, Washington University, St Louis, MO, USA. [41]Veterans Affairs San Diego Healthcare System, San Diego, CA, USA. [42]Department of Family Medicine and Public Health, University of California, San Diego, La Jolla, CA, USA. [43]Center for Cardiovascular Behavioral Health, Columbia University Medical Center, New York, NY, USA. [44]Genomics Program, College of Public Health, University of South Florida, Tampa FL. [45]Center for the Study of Traumatic Stress, Department of Psychiatry, Uniformed Services University of the Health Sciences, Bethesda, MD, USA. [46]Genomics Program, College of Public Health, University of South Florida, Tampa, FL, USA. [47]James J. Peters Bronx Veterans Affairs and Department of Psychiatry, Icahn School of Medicine at Mount Sinai, Bronx, NY, USA. [48]Department of Neuroscience, Icahn School of Medicine at Mount Sinai, Bronx, NY, USA. [49]Department of Biostatistics, Yale University, New Haven, CT, USA. [50]VA Ann Arbor Health System, Ann Arbor, MI, USA and [51]Department of Epidemiology, Harvard T. H. Chan School of Public Health, Cambridge, MA, USA.

Schizophrenia Working Group of the Psychiatric Genomics Consortium

Rolf Adolfsson[1], Ingrid Agartz[2], Esben Agerbo[3], Margot Albus[4], Madeline Alexander[5], Farooq Amin[6], Ole A. Andreassen[2], Silviu A. Bacanu[7], Martin Begemann[8], Richard A. Belliveau Jr[9], Judit Bene[10], Sarah E Bergen[11], Elizabeth Bevilacqua[9], Tim B. Bigdeli[7], Donald W. Black[12], Douglas H. R. Blackwood[13], Anders D. Børglum[14], Elvira Bramon[15], Richard Bruggeman[16], Nancy G. Buccola[17], Randy L. Buckner[18], Brendan Bulik-Sullivan[19], Joseph D Buxbaum[20], William Byerley[21], Wiepke Cahn[22], Guiqing Cai[20], Murray J. Cairns[23], Dominique Campion[24], Rita M. Cantor[25], Vaughan J. Carr[23], Noa Carrera[26], Stanley V. Catts[27], Kimberley D. Chambert[9], Raymond C. K. Chan[28], Eric Y. H. Chen[29], Ronald Y. L. Chen[30], Wei Cheng[31], Eric F. C. Cheung[32], Siow Ann Chong[33], Sven Cichon[34], C Robert Cloninger[35], David Cohen[36], Nadine Cohen[37], David A. Collier[38], Paul Cormican[39], Aiden Corvin[39], Nick Craddock[26], Benedicto Crespo-Facorro[40], James J. Crowley[41], David Curtis[42], Mark J. Daly[19], Ariel Darvasi[43], Michael Davidson[44], Kenneth L. Davis[20], Franziska Degenhardt[45], Jurgen Del Favero[46], Lynn E. DeLisi[47], Ditte Demontis[14], Dimitris Dikeos[48], Timothy Dinan[49], Srdjan Djurovic[2], Enrico Domenici[50], Gary Donohoe[51] , Elodie Drapeau[20], Jubao Duan[52], Frank Dudbridge[53], Hannelore Ehrenreich[8], Peter Eichhammer[54], Johan Eriksson[55], Valentina Escott-Price[26], Tõnu Esko[56], Laurent Essioux[57], Ayman H. Fanous[58], Kai-How Farh[19], Martilias S. Farrell[41], Josef Frank[59], Lude Franke[60], Robert Freedman[61], Nelson B. Freimer[62], Joseph I. Friedman[20], Menachem Fromer[20], Giulio Genovese[9], Elliot S. Gershon[63], Ina Giegling[64], Michael Gill[39] , Paola Giusti-Rodríguez[41], Stephanie Godard[65], Jacqueline I. Goldstein[19], Srihari Gopal[66], Jacob Gratten[67], Lieuwe de Haan[68], Agnes A. Steixner[8], Marian L. Hamshere[26], Mark Hansen[69], Thomas Hansen[70], Vahram Haroutunian[20], Annette M. Hartmann[64], Frans A. Henskens[71], Stefan Herms[34], Joel N. Hirschhorn[72], Per Hoffmann[34], Andrea Hofman[45], Mads V. Hollegaard[73], Peter A. Holmans[26], David M. Hougaard[73], Hailiang Huang[19], Christina M Hultman[11], Masashi Ikeda[74], Nakao Iwata[74], Assen V. Jablensky[75], Inge Joa[76] , Erik G. Jönsson[77], Antonio Julià[78], Anna K. Kähler[11], René

S. Kahn[22], Luba Kalaydjieva[79], Sena Karachanak-Yankova[80], Juha Karjalainen[60], David Kavanagh[26], Matthew C. Keller[81], Brian J. Kelly[82], Kenneth S. Kendler[83], James L. Kennedy[84], Andrey Khrunin[85], Yunjung Kim[41], George Kirov[26], Janis Klovins[86], Jo Knight[84], James A. Knowles[87], Bettina Konte[64], Vaidutis Kucinskas[88], Zita Ausrele Kucinskiene[88], Hana Kuzelova-Ptackova[89], Max Lam[33], Claudine Laurent[5], Phil Lee[90], S Hong Lee[67], Jimmy Lee Chee Keong[33], Sophie E. Legge[26], Todd Lencz[91], Bernard Lerer[92], Douglas F. Levinson[5], Miaoxin Li[93], Tao Li[94], Qingqin S Li[66], Kung-Yee Liang[95], Jeffrey Lieberman[96], Svetlana Limborska[85], Jianjun Liu[97], Jouko Lönnqvist[98], Carmel M. Loughland[23], Jan Lubinski[99], Milan Macek Jr[89], Patrik K. E. Magnusson[11], Brion S. Maher[100], Wolfgang Maier[101], Anil K. Malhotra[102], Jacques Mallet[103], Sara Marsal[78], Manuel Mattheisen[14], Morten Mattingsdal[2], Robert W. McCarley[47], Steven A. McCarroll[9], Colm McDonald[104], Andrew McQuillin[105], Sandra Meier[106], Carin J. Meijer[68], Bela Melegh[10], Ingrid Melle[2], Raquelle I. Mesholam-Gately[107], Andres Metspalu[56], Patricia T. Michie[108], Lili Milani[56], Vihra Milanova[109], Younes Mokrab[38], Jennifer L. Moran[9], Derek W. Morris[51], Ole Mors[110], Preben B. Mortensen[3], Bryan J. Mowry[67], Bertram Müller-Myhsok[111], Kieran C. Murphy[112], Robin M. Murray[113], Inez Myin-Germeys[114], Benjamin M. Neale[19], Mari Nelis[56], Igor Nenadic[115], Deborah A. Nertney[116], Gerald Nestadt[117], Kristin K. Nicodemus[118], Liene Nikitina-Zake[86], Laura Nisenbaum[119], Annelie Nordin[77], Nina Norgren[97], Markus M. Nöthen[45], Eadbhard O'Callaghan[120], Michael C. O'Donovan[26], Colm O'Dushlaine[9], F. Anthony O'Neill[121], Sang-Yun Oh[122], Ann Olincy[61], Line Olsen[70], Roel A. Ophoff[62], Jim Van Os[113], Michael J. Owen[26], Sara A. Paciga[123], Aarno Palotie[124], Christos Pantelis[125], George N. Papadimitriou[48], Marina Mitjans[8], Elena Parkhomenko[20], Michele T. Pato[87], Carlos N. Pato[87], Tiina Paunio[126], Psychosis Endophenotypes International Consortium[127], Diana O. Perkins[128], Tune H. Pers[129], Tracey L. Petryshen[130], Olli Pietiläinen[131], Jonathan Pimm[105], Andrew J. Pocklington[26], Danielle Posthuma[132], John Powell[113], Alkes Price[133], Ann E. Pulver[117], Shaun M. Purcell[20], Digby Quested[134], Henrik B. Rasmussen[70], Abraham Reichenberg[20], Mark A. Reimers[83], Alexander L. Richards[26], Marcella Rietschel[59], Brien P. Riley[83], Stephan Ripke[19], Joshua L. Roffman[130], Panos Roussos[20], Douglas M. Ruderfer[20], Dan Rujescu[64], Veikko Salomaa[136], Alan R. Sanders[52], Ulrich Schall[82], Thomas G. Schulze[137], Sibylle G. Schwab[138], Edward M. Scolnick[9], Rodney J. Scott[139], Larry J. Seidman[107], Pak C. Sham[30], Jianxin Shi[140], Jeremy M. Silverman[20], Kang Sim[33], Pamela Sklar[20], Petr Slominsky[85], Jordan W. Smoller[90], Hon-Cheong So[30], Erik Söderman[77], Chris C. A. Spencer[141], David St Clair[142], Eli A. Stahl[20], Elisabeth Stogmann[143] , Richard E. Straub[144], Eric Strengman[145], Jana Strohmaier[59], T. Scott Stroup[96], Mythily Subramaniam[33], Patrick F. Sullivan[41], Jaana Suvisaari[98], Dragan M. Svrakic[35], Jin P. Szatkiewicz[41], Srinivas Thirumalai[146], Draga Toncheva[147], Paul A. Tooney[139], Sarah Tosato[148], Peter M. Visscher[67], John Waddington[149], Dermot Walsh[150], James T. R. Walters[26], Dai Wang[66], Qiang Wang[94], Bradley T. Webb[7], Daniel R. Weinberger[144], Mark Weiser[44], Thomas Werge[70], Dieter B. Wildenauer[75], Nigel M. Williams[26], Stephanie Williams[41], Stephanie H. Witt[59], Aaron R. Wolen[7], Emily H. M. Wong[30], Brandon K. Wormley[83], Naomi R. Wray[67], Wellcome Trust Case-Control Consortium 2[153] , Jing Qin Wu[139], Hualin Simon Xi[154], Clement C. Zai[84], Xuebin Zheng[97], Fritz Zimprich[143]

[1]Department of Clinical Sciences, Psychiatry, Umeå University, SE-901 87 Umeå, Sweden. [2]NORMENT, KG Jebsen Centre for Psychosis Research, Institute of Clinical Medicine, University of Oslo, 0424 Oslo, Norway. [3]National Centre for Register-based Research, Aarhus University, DK-8210 Aarhus, Denmark. [4]State Mental Hospital, 85540 Haar, Germany. [5]Department of Psychiatry and Behavioral Sciences, Stanford University, Stanford, California 94305, USA. [6]Department of Psychiatry and Behavioral Sciences, Emory University, Atlanta, Georgia 30322, USA. [7]Virginia Institute for Psychiatric and Behavioral

Genetics, Department of Psychiatry, Virginia Commonwealth University, Richmond, Virginia 23298, USA. [8]Clinical Neuroscience, Max Planck Institute of Experimental Medicine, Göttingen 37075, Germany. [9]Stanley Center for Psychiatric Research, Broad Institute of MIT and Harvard, Cambridge, Massachusetts 02142, USA. [10]Department of Medical Genetics, University of Pécs, Pécs H-7624, Hungary. [11]Department of Medical Epidemiology and Biostatistics, Karolinska Institutet, Stockholm SE-17177, Sweden. [12]Department of Psychiatry, University of Iowa Carver College of Medicine, Iowa City, Iowa 52242, USA. [13]Division of Psychiatry, University of Edinburgh, Edinburgh EH10 5HF, UK. [14]Department of Biomedicine, University, DK-8000 Aarhus C, Denmark. [15]University College London, London WC1E 6BT, UK. [16]University Medical Center Groningen, Department of Psychiatry, University of Groningen, NL-9700 RB, The Netherlands. [17]School of Nursing, Louisiana State University Health Sciences Center, New Orleans, Louisiana 70112, USA. [18]Center for Brain Science, Harvard University, Cambridge, Massachusetts 02138, USA. [19]Analytic and Translational Genetics Unit, Massachusetts General Hospital, Boston, Massachusetts 02114, USA. [20]Department of Psychiatry, Icahn School of Medicine at Mount Sinai, New York, New York 10029, USA. [21]Department of Psychiatry, University of California at San Francisco, San Francisco, California, 94143 USA. [22]University Medical Center Utrecht, Department of Psychiatry, Rudolf Magnus Institute of Neuroscience, 3584 Utrecht, The Netherlands. [23]Schizophrenia Research Institute, Sydney NSW 2010, Australia. [24]Centre Hospitalier du Rouvray and INSERM U1079 Faculty of Medicine, 76301 Rouen, France. [25]Department of Human Genetics, David Geffen School of Medicine, University of California, Los Angeles, California 90095, USA. [26]MRC Centre for Neuropsychiatric Genetics and Genomics, Institute of Psychological Medicine and Clinical Neurosciences, School of Medicine, Cardiff University, Cardiff, CF24 4HQ, UK. [27]Royal Brisbane and Women's Hospital, University of Queensland, Brisbane QLD 4072, Australia. [28]Institute of Psychology, Chinese Academy of Science, Beijing 100101, China. [29]State Key Laboratory for Brain and Cognitive Sciences, Li Ka Shing Faculty of Medicine, The University of Hong Kong, Hong Kong, China. [30]Department of Psychiatry, Li Ka Shing Faculty of Medicine, The University of Hong Kong, Hong Kong, China. [31]Department of Computer Science, University of North Carolina, Chapel Hill, North Carolina 27514, USA. [32]Castle Peak Hospital, Hong Kong, China. [33]Institute of Mental Health, Singapore 539747, Singapore. [34]Division of Medical Genetics, Department of Biomedicine, University of Basel, Basel, CH-4058, Switzerland. [35]Department of Psychiatry, Washington University, St. Louis, Missouri 63110, USA. [36]Department of Child and Adolescent Psychiatry, Assistance Publique Hospitaux de Paris, Pierre and Marie Curie Faculty of Medicine and Institute for Intelligent Systems and Robotics, Paris, 75013, France. [37]Blue Note Biosciences, Princeton, New Jersey 08540, USA. [38]Eli Lilly and Company Limited, Erl Wood Manor, Sunninghill Road, Windlesham, Surrey, GU20 6PH, UK. [39]Neuropsychiatric Genetics Research Group, Department of Psychiatry, Trinity College Dublin, Dublin 8, Ireland. [40]University Hospital Marqués de Valdecilla, Instituto de Formación e Investigación Marqués de Valdecilla, University of Cantabria, E-39008 Santander, Spain. [41]Department of Genetics, University of North Carolina, Chapel Hill, North Carolina 27599-7264, USA. [42]Department of Psychological Medicine, Queen Mary University of London, London E1 1BB, UK. [43]Department of Genetics, The Hebrew University of Jerusalem, 91905 Jerusalem, Israel. [44]Sheba Medical Center, Tel Hashomer 52621, Israel. [45]Institute of Human Genetics, University of Bonn, D-53127 Bonn, Germany. [46]Applied Molecular Genomics Unit, VIB Department of Molecular Genetics, University of Antwerp, B-2610 Antwerp, Belgium. [47]VA Boston Health Care System, Brockton, Massachusetts 02301, USA. [48]First Department of Psychiatry, University of Athens Medical School, Athens 11528, Greece. [49]Department of Psychiatry, University College Cork, Co. Cork, Ireland. [50]Centre for Integrative Biology,

University of Trento, Trento, Italy. [51]Cognitive Genetics and Therapy Group, School of Psychology and Discipline of Biochemistry, National University of Ireland Galway, Co. Galway, Ireland. [52]Department of Psychiatry and Behavioral Sciences, NorthShore University HealthSystem, Evanston, Illinois 60201, USA. [53]Department of Non-Communicable Disease Epidemiology, London School of Hygiene and Tropical Medicine, London WC1E 7HT, UK. [54]Department of Psychiatry, University of Regensburg, 93053 Regensburg, Germany. [55]Folkhälsan Research Center, Helsinki, Finland, Biomedicum Helsinki 1, Haartmaninkatu 8, FI-00290, Helsinki, Finland. [56]Estonian Genome Center, University of Tartu, Tartu 50090, Estonia. [57]Translational Technologies and Bioinformatics, Pharma Research and Early Development, F.Hoffman-La Roche, CH-4070 Basel, Switzerland. [58]Mental Health Service Line, Washington VA Medical Center, Washington DC 20422, USA. [59]Department of Genetic Epidemiology in Psychiatry, Central Institute of Mental Health, Medical Faculty Mannheim, University of Heidelberg, Heidelberg, D-68159 Mannheim, Germany. [60]Department of Genetics, University of Groningen, University Medical Centre Groningen, 9700 RB Groningen, The Netherlands. [61]Department of Psychiatry, University of Colorado Denver, Aurora, Colorado 80045, USA. [62]Center for Neurobehavioral Genetics, Semel Institute for Neuroscience and Human Behavior, University of California, Los Angeles, California 90095, USA. [63]Departments of Psychiatry and Human Genetics, University of Chicago, Chicago, Illinois 60637 USA. [64]Department of Psychiatry, University of Halle, 06112 Halle, Germany. [65]Departments of Psychiatry and Human and Molecular Genetics, INSERM, Institut de Myologie, Hôpital de la Pitiè-Salpêtrière, Paris, 75013, France. [66]Neuroscience Therapeutic Area, Janssen Research and Development, Raritan, New Jersey 08869, USA. [67]Queensland Brain Institute, The University of Queensland, Brisbane, QLD 4072, Australia. [68]Academic Medical Centre University of Amsterdam, Department of Psychiatry, 1105 AZ Amsterdam, The Netherlands. [69]Illumina, La Jolla, California, California 92122, USA. [70]Institute of Biological Psychiatry, Mental Health Centre Sct. Hans, Mental Health Services Copenhagen, DK-4000, Denmark. [71]School of Electrical Engineering and Computer Science, University of Newcastle, Newcastle NSW 2308, Australia. [72]Department of Genetics, Harvard Medical School, Boston, Massachusetts 02115, USA. [73]Section of Neonatal Screening and Hormones, Department of Clinical Biochemistry, Immunology and Genetics, Statens Serum Institut, Copenhagen, DK-2300, Denmark. [74]Department of Psychiatry, Fujita Health University School of Medicine, Toyoake, Aichi, 470-1192, Japan. [75]School of Psychiatry and Clinical Neurosciences, The University of Western Australia, Perth, WA 6009, Australia. [76]Regional Centre for Clinical Research in Psychosis, Department of Psychiatry, Stavanger University Hospital, 4011 Stavanger, Norway. [77]Department of Clinical Neuroscience, Psychiatry Section, Karolinska Institutet, SE-17176 Stockholm, Sweden. [78]Rheumatology Research Group, Vall d'Hebron Research Institute, Barcelona, 08035, Spain. [79]Centre for Medical Research, The University of Western Australia, Perth, WA 6009, Australia. [80]Department of Medical Genetics, Medical University, Sofia 1431, Bulgaria. [81]Department of Psychology, University of Colorado Boulder, Boulder, Colorado 80309, USA. [82]Priority Centre for Translational Neuroscience and Mental Health, University of Newcastle, Newcastle NSW 2300, Australia. [83]Virginia Institute for Psychiatric and Behavioral Genetics, Departments of Psychiatry and Human and Molecular Genetics, Virginia Commonwealth University, Richmond, Virginia 23298, USA. [84]Lancaster Medical School and Data Science Institute, Lancaster University, Bailrigg, LA1 4YG, UK. [85]Institute of Molecular Genetics, Russian Academy of Sciences, Moscow 123182, Russia. [86]Latvian Biomedical Research and Study Centre, Riga, LV-1067, Latvia. [87]Department of Psychiatry and Zilkha Neurogenetics Institute, Keck School of Medicine at University of Southern California, Los Angeles, California 90089, USA. [88]Faculty of Medicine, Vilnius University, LT-01513 Vilnius,

Lithuania. [89]Department of Biology and Medical Genetics, 2nd Faculty of Medicine and University Hospital Motol, 150 06 Prague, Czech Republic. [90]Psychiatric and Neurodevelopmental Genetics Unit, Massachusetts General Hospital, Boston, Massachusetts 02114, USA. [91]Hofstra Northwell School of Medicine, Hempstead, New York 11549, USA [92]Department of Psychiatry, Hadassah-Hebrew University Medical Center, Jerusalem 91120, Israel. [93]Centre for Genomic Sciences, The University of Hong Kong, Hong Kong, China. [94]Mental Health Centre and Psychiatric Laboratory, West China Hospital, Sichuan University, Chengdu, 610041, Sichuan, China. [95]Department of Biostatistics, Johns Hopkins University Bloomberg School of Public Health, Baltimore, Maryland 21205, USA. [96]Department of Psychiatry, Columbia University, New York, New York 10032, USA. [97]Human Genetics, Genome Institute of Singapore, A*STAR, Singapore 138672, Singapore. [98]Department of Mental Health and Substance Abuse Services; National Institute for Health and Welfare, P.O. BOX 30, FI-00271 Helsinki, Finland. [99]Department of Genetics and Pathology, International Hereditary Cancer Center, Pomeranian Medical University in Szczecin, 70-453 Szczecin, Poland. [100]Department of Mental Health, Bloomberg School of Public Health, Johns Hopkins University, Baltimore, Maryland 21205, USA. [101]Department of Psychiatry, University of Bonn, D-53127 Bonn, Germany. [102]The Zucker Hillside Hospital, Glen Oaks, New York 11004, USA. [103]Centre National de la Recherche Scientifique, Laboratoire de Génétique Moléculaire de la Neurotransmission et des Processus Neurodégénératifs, Hôpital de la Pitié Salpêtrière, 75013, Paris, France. [104]Department of Psychiatry, National University of Ireland Galway, Co. Galway, Ireland. [105]Molecular Psychiatry Laboratory, Division of Psychiatry, University College London, London WC1E 6JJ, UK. [106]Department of Genetic Epidemiology in Psychiatry, Central Institute of Mental Health, Medical Faculty Mannheim, University of Heidelberg, Heidelberg,D-68159 Mannheim, Germany. [107]Massachusetts Mental Health Center Public Psychiatry Division of the Beth Israel Deaconess Medical Center, Boston, Massachusetts 02114, USA. [108]School of Psychology, University of Newcastle, Newcastle NSW 2308, Australia. [109]First Psychiatric Clinic, Medical University, Sofia 1431, Bulgaria. [110]Department P, Aarhus University Hospital, DK-8240 Risskov, Denmark. [111]Max Planck Institute of Psychiatry, 80336 Munich, Germany. [112]Department of Psychiatry, Royal College of Surgeons in Ireland, Dublin 2, Ireland. [113]King's College London, London SE5 8AF, UK. [114]Maastricht University Medical Centre, South Limburg Mental Health Research and Teaching Network, EURON, 6229 HX Maastricht, The Netherlands. [115]Department of Psychiatry and Psychotherapy, Jena University Hospital, 07743 Jena, Germany. [116]Queensland Centre for Mental Health Research, University of Queensland, Brisbane QLD 4076, Australia. [117]Department of Psychiatry and Behavioral Sciences, Johns Hopkins University School of Medicine, Baltimore, Maryland 21205, USA. [118]Department of Psychiatry, Trinity College Dublin, Dublin 2, Ireland. [119]Eli Lilly and Company, Lilly Corporate Center, Indianapolis, 46285 Indiana, USA. [120]DETECT Early Intervention Service for Psychosis, Blackrock, Co. Dublin, Ireland. [121]Centre for Public Health, Institute of Clinical Sciences, Queen's University Belfast, Belfast BT12 6AB, UK. [122]Lawrence Berkeley National Laboratory, University of California at Berkeley, Berkeley, California 94720, USA. [123]Human Genetics and Computational Biomedicine, Pfizer Global Research and Development, Groton, Connecticut 06340, USA. [124]Institute for Molecular Medicine Finland, FIMM, University of Helsinki, P.O. BOX 20, FI-00014, Helsinki, Finland. [125]Melbourne Neuropsychiatry Centre, University of Melbourne & Melbourne Health, Melbourne VIC 3053, Australia. [126]Public Health Genomics Unit, National Institute for Health and Welfare, P.O. BOX 30, FI-00271 Helsinki, Finland. [127]PEIC. [128]Department of Psychiatry, University of North Carolina, Chapel Hill, North Carolina 27599-7160, USA. [129]Center for Biological Sequence Analysis, Department of Systems Biology, Technical University of Denmark, DK-2800, Denmark. [130]Center for

Human Genetic Research and Department of Psychiatry, Massachusetts General Hospital, Boston, Massachusetts 02114, USA. [131]Institute for Molecular Medicine Finland, FIMM, University of Helsinki, P.O. BOX 20 FI-00014, Helsinki, Finland. [132]Department of Functional Genomics, Center for Neurogenomics and Cognitive Research, Neuroscience Campus Amsterdam, VU University, Amsterdam 1081, The Netherlands. [133]Department of Epidemiology, Harvard School of Public Health, Boston, Massachusetts 02115, USA. [134]Department of Psychiatry, University of Oxford, Oxford, OX3 7JX, UK. [135]Virginia Institute for Psychiatric and Behavioral Genetics, Virginia Commonwealth University, Richmond, Virginia 23298, USA. [136]National Institute for Health and Welfare, P.O BOX 30, FI-00271 Helsinki, Finland. [137]Department of Psychiatry and Psychotherapy, University of Göttingen, 37073 Göttingen, Germany. [138]Psychiatry and Psychotherapy Clinic, University of Erlangen, 91054 Erlangen, Germany.
[139]School of Biomedical Sciences and Pharmacy, University of Newcastle, Callaghan NSW 2308, Australia. [140]Division of Cancer Epidemiology and Genetics, National Cancer Institute, Bethesda, Maryland 20892, USA. [141]Wellcome Trust Centre for Human Genetics, Oxford, OX3 7BN, UK. [142]University of Aberdeen, Institute of Medical Sciences, Aberdeen, AB25 2ZD, UK. [143]Department of Clinical Neurology, Medical University of Vienna, 1090 Wien, Austria. [144]Lieber Institute for Brain Development, Baltimore, Maryland 21205, USA. [145]Department of Medical Genetics, University Medical Centre Utrecht, Universiteitsweg 100, 3584 CG, Utrecht, The Netherlands. [146]Berkshire Healthcare NHS Foundation Trust, Bracknell RG12 1BQ, UK. [147]Department of Medical Genetics, Medical University, Sofia1431, Bulgaria. [148]Section of Psychiatry, University of Verona, 37134 Verona, Italy [149]Molecular and Cellular Therapeutics, Royal College of Surgeons in Ireland, Dublin 2, Ireland. [150]Health Research Board, Dublin 2, Ireland. [151]Mental Health Centre and Psychiatric Laboratory, West China Hospital, Sichuan University, Chendu, 610041, Sichuan, China [152]School of Psychiatry and Clinical Neurosciences, The University of Western Australia, Perth WA 6009, Australia. [153]WTCCC2. [154]Computational Sciences CoE, Pfizer Worldwide Research and Development, Cambridge, Massachusetts 02139, USA.

Sex Differences Cross Disorder Working Group of the Psychiatric Genomics Consortium

Martin Alda[1], Gabriëlla A. Blokland[2,3], Janita Bralten[4], Christie L. Burton[5], Enda Byrne[6], Caitlin E. Carey[7,8], Lea K. Davis[9], Stephen V. Faraone[10], Jill M. Goldstein[11,12], Slavina Goleva[13], Wei Guo[14], Laura M. Huckins[15], Ekaterina A. Khramtsova[16], Phil H Lee[17], Joanna Martin[18], Carol A. Mathews[19], Manuel Mattheisen[20], Benjamin M. Neale[8,7], Roseann Peterson[21], Tracey L. Petryshen[17], Jordan W. Smoller[17], Barbara E. Stranger[16], Michela Traglia[22,23,24], Lauren M. Weiss[22,23,24], Stacey Winham[25], Naomi Wray[6]

[1]Department of Psychiatry, Dalhousie University, Halifax, NS, Canada. [2]Department of Psychiatry and Neuropsychology, School for Mental Health and Neuroscience, Faculty of Health, Medicine, and Life Sciences, Maastricht University, Maastricht, the Netherlands. [3]Psychiatric and Neurodevelopmental Genetics Unit, Department of Psychiatry and Center for Genomic Medicine, Massachusetts General Hospital, Harvard Medical School, Boston, MA, USA. [4]Department of Human Genetics, Radboud University Medical Center, Nijmegen, the Netherlands. [5]Neurosciences and Mental Health, Hospital for Sick Children, Toronto, ON, Canada. [6]Institute for Molecular Bioscience, University of Queensland, Brisbane, Australia. [7]Broad Institute, Stanley Center for Psychiatric Research, Cambridge, MA, USA. [8]Analytic and Translational Genetics Unit, Massachusetts General Hospital, Harvard Medical School, Boston, MA, USA. [9]Department of Medicine; Department of Psychiatry and Behavioral Sciences, Division of Genetic Medicine, Vanderbilt University Medical Center,

Nashville, TN, USA. [10]Departments of Psychiatry and of Neuroscience and Physiology, SUNY Upstate Medical University, Syracuse, NY, USA. [11]Department of Psychiatry, Massachusetts General Hospital, Harvard Medical School, Boston, MA, USA. [12]Department of Obstetrics and Gynecology, Massachusetts General Hospital, Harvard Medical School, Boston, MA, USA. [13]Vanderbilt University, Nashville, TN, USA. [14]National Institutes of Health, Bethesda, MD, USA. [15]Pamela Sklar Division of Psychiatric Genetics, Icahn School of Medicine at Mount Sinai, New York City, NY, USA. [16]Section of Genetic Medicine, Department of Medicine and Institute for Genomics and Systems Biology, University of Chicago, Chicago, IL, USA. [17]Massachusetts General Hospital, Harvard Medical School, Boston, MA, USA. [18]MRC Centre for Neuropsychiatric Genetics and Genomics, Cardiff University, Cardiff, GB. [19]Department of Psychiatry and Genetics Institute, University of Florida, Gainesville, FL, USA. [20]Department of Psychiatry, Psychosomatics and Psychotherapy, University of Würzburg, Würzburg, Germany. [21]Virginia Commonwealth University, Richmond, VA, USA. [22]Department of Psychiatry, University of California San Francisco, San Francisco, CA, USA. [23]Institute for Human Genetics, University of California San Francisco, San Francisco, CA, USA. [24]Weill Institute for Neurosciences, University of California San Francisco, San Francisco, CA, USA. [25]Department of Health Sciences Research, Division of Biomedical Statistics and Informatics, Mayo Clinic, Rochester, MN, USA

Substance Use Disorder Working Group of the Psychiatric Genomics Consortium
Raymond K. Walters[1,2], Renato Polimanti[3], Emma C. Johnson[4], Jeanette N. McClintick[5], Mark J. Adams[6], Amy E. Adkins[7], Fazil Aliev[8], Silviu-Alin Bacanu[9], Anthony Batzler[10], Sarah Bertelsen[11], Joanna M. Biernacka[12], Tim B. Bigdeli[13], Li-Shiun Chen[4], Toni-Kim Clarke[6], Yi-Ling Chou[4], Franziska Degenhardt[14], Anna R. Docherty[15], Alexis C. Edwards[16], Jerome C. Foo[17], Louis Fox[4], Josef Frank[17], Ina Giegling[18], Scott Gordon[19], Laura M. Hack[20], Annette M. Hartmann[18], Sarah M. Hartz[4], Stefanie Heilmann- Heimbach[14], Stefan Herms[14,21], Colin Hodgkinson[22], Per Hoffmann[14,21], Jouke Jan Hottenga[23], Martin A. Kennedy[24], Mervi Alanne-Kinnunen[25], Bettina Konte[18], Jari Lahti[26,27], Marius Lahti-Pulkkinen[27], Dongbing Lai[28], Lannie Ligthart[23], Anu Loukola[25], Brion S. Maher[29], Hamdi Mbarek[23], Andrew M. McIntosh[30], Matthew B. McQueen[31], Jacquelyn L. Meyers[32], Yuri Milaneschi[33], Teemu Palviainen[25], John F. Pearson[34], Roseann E. Peterson[16], Samuli Ripatti[1,2,25,35], Euijung Ryu[36], Nancy L. Saccone[37], Jessica E. Salvatore[8,16], Sandra Sanchez- Roige[38], Melanie Schwandt[39], Richard Sherva[40], Fabian Streit[17], Jana Strohmaier[17], Nathaniel Thomas[7], Jen- Chyong Wang[11], Bradley T. Webb[9], Robbee Wedow[1,2,41,42], Leah Wetherill[28], Amanda G. Wills[43], Jason D. Boardman[44], Danfeng Chen[2], Doo-Sup Choi[45], William E. Copeland[46], Robert C. Culverhouse[47], Norbert Dahmen[48], Louisa Degenhardt[49], Benjamin W. Domingue[50], Mark A. Frye[51], Wolfgang Gäbel[52], Caroline Hayward[53], Marcus Ising[54], Margaret Keyes[55], Falk Kiefer[56], John Kramer[57], Samuel Kuperman[57], Susanne Lucae[54], Michael T. Lynskey[58], Wolfgang Maier[59], Karl Mann[56], Satu Männistö[60], Bertram Müller-Myhsok[61], Alison D. Murray[62], John I. Nurnberger[28,63], Aarno Palotie[1,2,25,64], Ulrich Preuss[18,65], Katri Räikkönen[27], Maureen D. Reynolds[66], Monika Ridinger[67], Norbert Scherbaum[68], Marc A. Schuckit[38], Michael Soyka[69,70], Jens Treutlein[17], Stephanie Witt[17], Norbert Wodarz[71], Peter Zill[70], Daniel E. Adkins[15,72], Joseph M. Boden[24], Dorret I. Boomsma[23], Laura J. Bierut[4], Sandra A. Brown[38,73], Kathleen K. Bucholz[4], Sven Cichon[21], E. Jane Costello[46], Harriet de Wit[74], Nancy Diazgranados[75], Danielle M. Dick[7,76], Johan G. Eriksson[77], Lindsay A. Farrer[40,78], Tatiana M. Foroud[28], Nathan A. Gillespie[16], Alison M. Goate[11], David Goldman[22,39], Richard A. Grucza[4], Dana B. Hancock[79], Kathleen Mullan Harris[80], Andrew C. Heath[4], Victor Hesselbrock[81], John K. Hewitt[82], Christian J. Hopfer[83], John Horwood[24], William Iacono[55], Eric O. Johnson[84], Jaakko A. Kaprio[25,35], Victor M. Karpyak[51], Kenneth S. Kendler[9], Henry R. Kranzler[85],

Kenneth Krauter[86], Paul Lichtenstein[87], Penelope A. Lind[19], Matt McGue[55], James MacKillop[88], Pamela A. F. Madden[4], Hermine H. Maes[89], Patrik Magnusson[87], Nicholas G. Martin[19], Sarah E. Medland[19], Grant W. Montgomery[90], Elliot C.Nelson[4], Markus M. Nöthen[91], Abraham A. Palmer[38,92], Nancy L. Pedersen[87], Brenda W. J. H. Penninx[33], Bernice Porjesz[32], John P. Rice[4], Marcella Rietschel[17], Brien P. Riley[9], Richard Rose[93], Dan Rujescu[18], Pei-Hong Shen[22], Judy Silberg[16], Michael C. Stallings[82], Ralph E. Tarter[66], Michael M. Vanyukov[66], Scott Vrieze[55], Tamara L. Wall[38], John B. Whitfield[19], Hongyu Zhao[94], Benjamin M. Neale[1,2], Joel Gelernter[95,*], Howard J. Edenberg[5,28,*], Arpana Agrawal[4,*]

[1]Analytic and Translational Genetics Unit, Department of Medicine, Massachusetts General Hospital and Harvard Medical School, Boston, Massachusetts, USA. [2]Stanley Center for Psychiatric Research, Broad Institute of MIT and Harvard, Cambridge, Massachusetts, USA. [3]Department of Psychiatry, Yale School of Medicine and VA CT Healthcare Center, West Haven, CT, USA. [4]Washington University School of Medicine, Department of Psychiatry, USA. [5]Department of Biochemistry and Molecular Biology, Indiana University School of Medicine, USA. [6]University of Edinburgh, Division of Psychiatry, Edinburgh, UK. [7]Department of Psychology & College Behavioral and Emotional Health Institute, Virginia Commonwealth University, USA. [8]Virginia Commonwealth University, Department of Psychology, USA. [9]Virginia Commonwealth University Alcohol Research Center; Virginia Institute for Psychiatric and Behavioral Genetics; Department of Psychiatry, Virginia Commonwealth University, USA. [10]Mayo Clinic, Psychiatric Genomics and Pharmacogenomics Program, USA. [11]Icahn School of Medicine at Mount Sinai, Department of Neuroscience, USA. [12]Mayo Clinic, Department of Health Sciences Research, and Department of Psychiatry and Psychology, USA. [13]Department of Psychiatry and Behavioral Sciences, State University of New York Downstate Medical Center, USA. [14]Institute of Human Genetics, University of Bonn; and Department of Genomics, Life & Brain Center, University of Bonn, Germany. [15]University of Utah, Department of Psychiatry, USA. [16]Virginia Commonwealth University, Virginia Institute for Psychiatric and Behavioral Genetics, Department of Psychiatry, USA. [17]Department of Genetic Epidemiology in Psychiatry, Central Institute of Mental Health, Medical Faculty Mannheim, Heidelberg University, Mannheim, Germany. [18]Martin-Luther-University Halle-Wittenberg, Department of Psychiatry, Psychotherapy and Psychosomatics, Germany. [19]QIMR Berghofer Medical Research Institute, Brisbane, Australia. [20]Department of Psychiatry and Behavioral Sciences, Emory University School of Medicine. [21]Human Genomics Research Group, Department of Biomedicine, University of Basel Institute of Medical Genetics and Pathology, University Hospital Basel, Switzerland. [22]NIH/NIAAA, Laboratory of Neurogenetics, NIAAA, NIH, USA. [23]Department of Biological Psychology, Amsterdam Public Health Research Institute, Vrije Universiteit Amsterdam, Amsterdam, the Netherlands. [24]University of Otago, Christchurch, New Zealand. [25]Institute for Molecular Medicine Finland (FIMM), University of Helsinki, Finland. [26]Helsinki Collegium for Advanced Studies, University of Helsinki, Helsinki, Finland. [27]Department of Psychology and Logopedics, University of Helsinki, Helsinki, Finland. [28]Department of Medical and Molecular Genetics, Indiana University School of Medicine, USA. [29]Johns Hopkins Bloomberg School of Public Health, USA. [30]University of Edinburgh, Division of Psychiatry; Centre for Cognitive Ageing and Cognitive Epidemiology, Edinburgh, USA. [31]Department of Integrative Physiology, University of Colorado Boulder, USA. [32]Henri Begleiter Neurodynamics Laboratory, Department of Psychiatry and Behavioral Sciences, SUNY Downstate Medical Center, USA. [33]Department of Psychiatry, Amsterdam Public Health Research Institute, VU University Medical Center/GGz inGeest, Amsterdam, the Netherlands. [34]Biostatistics and Computational Biology Unit, University of Otago, Christchurch, New Zealand. [35]Department of Public

Health, University of Helsinki, Helsinki, Finland. [36]Mayo Clinic, Department of Health Sciences Research, USA. [37]Washington University School of Medicine, Department of Genetics, USA. [38]University of California San Diego, Department of Psychiatry, San Diego, USA. [39]NIH/NIAAA, Office of the Clinical Director, USA. [40]Department of Medicine (Biomedical Genetics), Boston University School of Medicine, USA. [41]Department of Epidemiology, Harvard T.H. Chan School of Public Health, Boston, Massachusetts, USA. [42]Department of Sociology, Harvard University, Cambridge, Massachusetts, USA. [43]University of Colorado School of Medicine, Department of Pharmacology, USA. [44]Institute of Behavioral Science and Department of Sociology, University of Colorado, USA. [45]Mayo Clinic, Department of Molecular Pharmacology and Experimental Therapeutics, USA. [46]Duke University Medical Center, Department of Psychiatry and Behavioral Sciences, USA. [47]Washington University School of Medicine, Department of Medicine and Division of Biostatistics, USA. [48]Department of Psychiatry, University of Mainz, Mainz, Germany. [49]National Drug and Alcohol Research Centre, University of New South Wales, Australia. [50]Stanford University Graduate School of Education, USA. [51]Mayo Clinic, Department of Psychiatry and Psychology, USA. [52]Department of Psychiatry and Psychotherapy, University of Düsseldorf, Düsseldorf, Germany. [53]MRC Human Genetics Unit, Institute of Genetics and Molecular Medicine, University of Edinburgh, Edinburgh, UK. [54]Max-Planck-Institute of Psychiatry, Munich, Germany. [55]University of Minnesota, Department of Psychology, USA. [56]Department of Addictive Behavior and Addiction Medicine, Central Institute of Mental Health, Medical Faculty Mannheim, Heidelberg University. [57]University of Iowa Roy J and Lucille A Carver College of Medicine, Department of Psychiatry, USA. [58]Addictions Department, Institute of Psychiatry, Psychology & Neuroscience, King's College London, London, UK. [59]Department of Psychiatry, University of Bonn, Germany. [60]Institute for Health and Welfare, Finland. [61]Department of Statistical Genetics, Max-Planck-Institute of Psychiatry, Munich, Germany. [62]The Institute of Medical Sciences, Aberdeen Biomedical Imaging Centre, University of Aberdeen, UK. [63]Department of Psychiatry, Indiana University School of Medicine, USA. [64]Department of Medicine, Department of Neurology and Department of Psychiatry, Massachusetts General Hospital, Boston, MA, USA. [65]Vitos Hospital Herborn, Department of Psychiatry and Psychotherapy, Herborn, Germany. [66]University of Pittsburgh, School of Pharmacy, USA. [67]Department of Psychiatry and Psychotherapy, University of Regensburg Psychiatric Health Care Aargau, Germany. [68]LVR-Hospital Essen, Department of Psychiatry and Psychotherapy, Department of Addictive Behaviour and Addiction Medicine, Medical Faculty, University of Duisburg-Essen, Germany. [69]Medical Park Chiemseeblick in Bernau-Felden, Germany. [70]Psychiatric Hospital, Ludwig-Maximilians-University, München, Germany. [71]Department of Psychiatry and Psychotherapy, University of Regensburg, Germany. [72]University of Utah, Department of Sociology, USA. [73]University of California, San Diego School of Medicine, Department of Psychology, USA. [74]Department of Psychiatry and Behavioral Neuroscience, University of Chicago, University of Chicago Medical Centre, USA. [75]NIAAA Intramural Research Program, USA. [76]Department of Human & Molecular Genetics, Virginia Commonwealth University, USA. [77]Department of General Practice and Primary Health Care, University of Helsinki, Helsinki, Finland and National Institute for Health and Welfare, Finland. [78]Departments of Neurology, Ophthalmology, Epidemiology, and Biostatistics, Boston University Schools of Medicine and Public Health, USA. [79]Center for Omics Discovery and Epidemiology, Behavioral Health Research Division, RTI International. [80]Department of Sociology and Carolina Population Center, University of North Carolina at Chapel Hill, USA. [81]University of Connecticut School of Medicine, Department of Psychiatry, USA. [82]University of Colorado Boulder, Institute for Behavioral Genetics, USA. [83]University of Colorado Denver, School of Medicine, USA. [84]RTI International, Fellows Program.

[85]University of Pennsylvania Perelman School of Medicine, Center for Studies of Addiction, Department of Psychiatry and VISN 4 MIRECC, Crescenz VAMC. [86]University of Colorado Boulder, Department of Molecular, Cellular, and Developmental Biology, USA. [87]Department of Medical Epidemiology and Biostatistics, Karolinska Instituet, Stockholm, Sweden. [88]Peter Boris Centre for Addictions Research, McMaster University/St. Joseph's Healthcare Hamilton; Michael G. DeGroote Centre for Medicinal Cannabis Research. [89]Virginia Commonwealth University, Virginia Institute for Psychiatric and Behavioral Genetics, USA. [90]The Institute for Molecular Bioscience, University of Queensland, Australia. [91]Institute of Human Genetics, University of Bonn, School of Medicine & University Hospital Bonn, Germany. [92]University of California San Diego, Institute for Genomic Medicine, USA. [93]Department of Psychological & Brain Sciences, Indiana University, Bloomington, IN, USA. [94]Department of Biostatistics, Yale School of Public Health, Yale University, USA. [95]Departments of Psychiatry, Genetics, and Neuroscience, Yale University School of Medicine, VA Connecticut Healthcare System, USA.

German Borderline Genomics Consortium

Stephanie H. Witt[1], Fabian Streit[1], Martin Jungkunz[2,3], Josef Frank[1], Swapnil Awasthi[4], Jens Treutlein[1], Lydie Dietl[5], Cornelia E. Schwarze[6], Norbert Dahmen[8], Björn H. Schott[4,9], Markus M. Nöthen[10,11], Stephan Ripke[4,12,13], Arian Mobascher[8], Dan Rujescu[7], Klaus Lieb[8], Stephan Ripke[5], Christian Schmahl[2], Martin Bohus[3], Marcella Rietschel[1]

[1]Central Institute of Mental Health, Department of Genetic Epidemiology in Psychiatry, Medical Faculty Mannheim, Heidelberg University, Mannheim, Germany. [2]Central Institute of Mental Health, Clinic of Psychosomatic and Psychotherapeutic Medicine, Medical Faculty Mannheim, Heidelberg University, Mannheim, Germany. [3]Central Institute of Mental Health, Institute for Psychiatric and Psychosomatic Psychotherapy (IPPP) / Psychosomatic Medicine and Psychotherapy, Medical Faculty Mannheim, Heidelberg University, Mannheim, Germany. [4]Charité Universitätsmedizin Berlin, Department of Psychiatry and Psychotherapy, Campus Mitte, Berlin, Germany. [5]Charité-Universitätsmedizin Berlin, Department of Psychiatry, Campus Benjamin Franklin, Berlin, Germany. [6]University of Heidelberg, Department of Clinical Psychology and Psychotherapy, Heidelberg, Germany. [7]University of Halle, Department of Psychiatry, Halle, Germany. [8]University Medical Center, Department of Psychiatry and Psychotherapy, Mainz, Germany. [9]Leibniz Institute for Neurobiology, Magdeburg, Germany. [10]University of Bonn, Institute of Human Genetics, Bonn, Germany. [11]University of Bonn, Life&Brain Center, Department of Genomics, Bonn, Germany. [12]Broad Institute of MIT and Harvard, Stanley Center for Psychiatric Research and Medical and Population Genetics Program, Cambridge, MA, USA. [13]Massachusetts General Hospital and Department of Medicine, Harvard Medical School, Analytic and Translational Genetics Unit, Boston, MA, USA.

The International Headache Genetics Consortium.

Yuanhao Yang[1,2], Huiying Zhao[1,3], Lannie Ligthart[4], Andrea C. Belin[5], George Davey Smith[6], Tonu Esko[7,8,9], Tobias M. Freilinger[10,11], Thomas Folkmann Hansen[12], M. Arfan Ikram[13], Mikko Kallela[14], Christian Kubisch[15], Christofidou Paraskevi[16], David P. Strachan[17], Maija Wessman[18,19], Arn M. J. M. van den Maagdenberg[20,21], Gisela M. Terwindt[20], Dale R. Nyholt[1], Padhraig Gormley[7,22,23,24], Verneri Anttila[7,23,24], Bendik S. Winsvold[25,26,27], Priit Palta[18], Tune H. Pers[7,9,28,29], Kai-How Farh[7,30,31], Ester Cuenca-Leon[7,22,23,32], Mikko Muona[18,19,33,34], Nicholas A. Furlotte[35], Tobias Kurth[36,37], Andres Ingason[38], George McMahon[6], Gisela M. Terwindt[20], Mikko Kallela[39], Caroline Ran[5], Scott G. Gordon[40], Anine H. Stam[20], Stacy Steinberg[38], Guntram Borck[41], Markku Koiranen[42], Lydia Quaye[16], Hieab

H. H. Adams[13,43], Terho Lehtimäki[44], Antti-Pekka Sarin[18], Juho Wedenoja[45], David A. Hinds[35], Julie E. Buring[37,46], Markus Schürks[47], Paul M. Ridker[37,46], Maria Gudlaug Hrafnsdottir[48], Hreinn Stefansson[38], Susan M. Ring[6], Jouke-Jan Hottenga[4], Brenda W. J. H. Penninx[49], Markus Färkkilä[39], Ville Artto[39], Mari Kaunisto[18], Salli Vepsäläinen[39], Rainer Malik[11], Andrew C. Heath[50], Pamela A. F. Madden[50], Nicholas G. Martin[40], Grant W Montgomery[40], Mitja I Kurki[7,18,22,23,51], Mart Kals[8], Reedik Mägi[8], Kalle Pärn[8], Eija Hämäläinen[18], Hailiang Huang[7,23,30], Andrea E. Byrnes[7,23,30], Lude Franke[52], Jie Huang[24], Evie Stergiakouli[6], Phil H. Lee[7,22,23], Cynthia Sandor[53], Caleb Webber[53], Zameel Cader[54,55], Bertram Muller-Myhsok[21,56,85], Stefan Schreiber[57], Thomas Meitinger[58,59], Johan G. Eriksson[60,61], Veikko Salomaa[61], Kauko Heikkilä[62], Elizabeth Loehrer[13,63], Andre G. Uitterlinden[64], Albert Hofman[13], Cornelia M. van Duijn[13], Lynn Cherkas[16], Linda M. Pedersen[25], Audun Stubhaug[65,66], Christopher S. Nielsen[65,67], Minna Männikkö[42], Evelin Mihailov[8], Lili Milani[8], Hartmut Göbel[68], Ann-Louise Esserlind[69], Anne Francke Christensen[69], Thomas Folkmann Hansen[70], Thomas Werge[71,72,73], Jaakko Kaprio[18,45,74], Arpo J. Aromaa[61], Olli Raitakari[75,76], M. Arfan Ikram[13,43,77], Tim Spector[16], Marjo-Riitta Järvelin[42,78,79,80], Andres Metspalu[8], Christian Kubisch[15], David P. Strachan[17], Michel D. Ferrari[20], Andrea C. Belin[5], Martin Dichgans[11,81], Maija Wessman[18,54], John-Anker Zwart[25,26,27], Dorret I. Boomsma[4], Kari Stefansson[38,82] Nicholas Eriksson[35] Mark J. Daly[7,23,30] Benjamin M. Neale[7,23,30] Jes Olesen[69] Daniel I. Chasman[37,46] Dale R. Nyholt[83] Aarno Palotie[7,18,22,23,24,30,84]

[1]Statistical and Genomic Epidemiology Laboratory, Institute of Health and Biomedical Innovation, Queensland University of Technology, Brisbane, QLD, Australia. [2]Institute of Molecular Bioscience, The University of Queensland, Brisbane, QLD, Australia. [3]Guangdong Provincial Key Laboratory of Malignant Tumor Epigenetics and Gene Regulation, Sun Yat-sen Memorial Hospital, Sun Yat-sen University, Guangzhou, People's Republic of China. [4]Department of Biological Psychology, Vrije Universiteit, Amsterdam, The Netherlands. [5]Department of Neuroscience, Karolinska Institutet, Stockholm, Sweden. [6]Medical Research Council (MRC) Integrative Epidemiology Unit, University of Bristol, Bristol, UK. [7]Medical and Population Genetics Program, Broad Institute of MIT and Harvard, Cambridge, MA, USA. [8]Estonian Genome Center, University of Tartu, Tartu, Estonia. [9]Division of Endocrinology, Boston Children's Hospital, Boston, MA, USA. [10]Department of Neurology and Epileptology, Hertie-Institute for Clinical Brain Research, University of Tübingen, Tübingen, Germany. [11]Institute for Stroke and Dementia Research, Klinikum der Universität München, Ludwig-Maximilians-Universität München, Munich, Germany. [12]Danish Headache Center, Department of Neurology, Rigshospitalet, Glostrup Hospital, University of Copenhagen, Copenhagen, Denmark. [13]Department of Epidemiology, Erasmus University Medical Center, Rotterdam, The Netherlands. [14]Department of Neurology, Helsinki University Central Hospital, Helsinki, Finland. [15]Institute of Human Genetics, University Medical Center Hamburg-Eppendorf, Hamburg, Germany. [16]Department of Twin Research and Genetic Epidemiology, King's College London, London, UK. [17]Population Health Research Institute, St George's, University of London, London, UK. [18]Institute for Molecular Medicine Finland (FIMM), University of Helsinki, Helsinki, Finland. [19]Folkhälsan Institute of Genetics, Helsinki, Finland. [20]Department of Neurology, Leiden University Medical Center, Leiden, The Netherlands. [21]Department of Human Genetics, Leiden University Medical Center, Leiden, The Netherlands. [22]Psychiatric and Neurodevelopmental Genetics Unit, Massachusetts General Hospital and Harvard Medical School, Boston, MA, USA. [23]Stanley Center for Psychiatric Research, Broad Institute of MIT and Harvard, Cambridge, MA, USA. [24]Wellcome Trust Sanger Institute, Wellcome Trust Genome Campus, Hinxton, UK [25]FORMI, Oslo University Hospital, Oslo, Norway.

[26]Department of Neurology, Oslo University Hospital, Oslo, Norway. [27]Institute of Clinical Medicine, University of Oslo, Oslo, Norway. [28]Department of Epidemiology Research, Statens Serum Institut, Copenhagen, Denmark. [29]Novo Nordisk Foundation Center for Basic Metabolic Research, University of Copenhagen, Copenhagen, Denmark. [30]Analytic and Translational Genetics Unit, Massachusetts General Hospital and Harvard Medical School, Boston, MA, USA. [31]Illumina, San Diego, CA, USA. [32]Pediatric Neurology, Vall d'Hebron Research Institute, Barcelona, Spain. [33]Neuroscience Center, University of Helsinki, Helsinki, Finland. [34]Molecular Neurology Research Program, Research Programs Unit, University of Helsinki, Helsinki, Finland. [35]23andMe, Inc., Mountain View, CA, USA. [36]Institute of Public Health, Charité–Universitätsmedizin Berlin, Berlin, Germany. [37]Division of Preventive Medicine, Brigham and Women's Hospital, Boston, MA, USA. [38]deCODE Genetics, Reykjavik, Iceland. [39]Department of Neurology, Helsinki University Central Hospital, Helsinki, Finland. [40]Department of Genetics and Computational Biology, QIMR Berghofer Medical Research Institute, Brisbane, Queensland, Australia. [41]Institute of Human Genetics, Ulm University, Ulm, Germany. [42]Center for Life Course Epidemiology and Systems Medicine, University of Oulu, Oulu, Finland. [43]Department of Radiology, Erasmus University Medical Center, Rotterdam, The Netherlands. [44]Department of Clinical Chemistry, Fimlab Laboratories, School of Medicine, University of Tampere, Tampere, Finland. [45]Department of Public Health, University of Helsinki, Helsinki, Finland. [46]Harvard Medical School, Boston, MA, USA. [47]Department of Neurology, University Duisburg–Essen, Essen, Germany. [48]Landspitali University Hospital, Reykjavik, Iceland. [49]Department of Psychiatry, VU University Medical Center, Amsterdam, The Netherlands. [50]Department of Psychiatry, Washington University School of Medicine, St. Louis, MO, USA. [51]Department of Neurosurgery, NeuroCenter, Kuopio University Hospital, Kuopio, Finland. [52]Department of Genetics, University Medical Center Groningen, University of Groningen, Groningen, The Netherlands. [53]MRC Functional Genomics Unit, Department of Physiology, Anatomy and Genetics, Oxford University, Oxford, UK. [54]Nuffield Department of Clinical Neuroscience, University of Oxford, Oxford, UK. [55]Oxford Headache Centre, John Radcliffe Hospital, Oxford, UK. [56]Max Planck Institute of Psychiatry, Munich, Germany. [57]Institute of Clinical Molecular Biology, Christian Albrechts University, Kiel, Germany. [58]Institute of Human Genetics, Helmholtz Zentrum München, Neuherberg, Germany. [59]Institute of Human Genetics, Technische Universität München, Munich, Germany. [60]Department of General Practice and Primary Health Care, University of Helsinki and Helsinki University Hospital, Helsinki, Finland. [61]National Institute for Health and Welfare, Helsinki, Finland. [62]Institute of Clinical Medicine, University of Helsinki, Helsinki, Finland. [63]Department of Environmental Health, Harvard T.H. Chan School of Public Health, Boston, MA, USA. [64]Department of Internal Medicine, Erasmus University Medical Center, Rotterdam, The Netherlands. [65]Department of Pain Management and Research, Oslo University Hospital, Oslo, Norway.[66]Medical Faculty, University of Oslo, Oslo, Norway. [67]Department of Ageing and Health, Norwegian Institute of Public Health, Oslo, Norway. [68]Kiel Pain and Headache Center, Kiel, Germany. [69]Danish Headache Center, Department of Neurology, Rigshospitalet, Glostrup Hospital, University of Copenhagen, Copenhagen, Denmark. [70]Institute of Biological Psychiatry, Mental Health Center Sct. Hans, University of Copenhagen, Roskilde, Denmark. [71]Institute of Biological Psychiatry, MHC Sct. Hans, Mental Health Services Copenhagen, Copenhagen, Denmark. [72]Institute of Clinical Sciences, Faculty of Medicine and Health Sciences, University of Copenhagen, Copenhagen, Denmark. [73]iPSYCH—The Lundbeck Foundation Initiative for Integrative Psychiatric Research, Copenhagen, Denmark. [74]Department of Health, National Institute for Health and Welfare, Helsinki, Finland. [75]Research Center of Applied and Preventive Cardiovascular Medicine, University of Turku, Turku, Finland. [76]Department of Clinical Physiology and Nuclear Medicine, Turku

University Hospital, Turku, Finland. [77]Department of Neurology, Erasmus University Medical Center, Rotterdam, The Netherlands. [78]Department of Epidemiology and Biostatistics, MRC Health Protection Agency (HPE) Centre for Environment and Health, School of Public Health, Imperial College London, London, UK. [79]Biocenter Oulu, University of Oulu, Oulu, Finland. [80]Unit of Primary Care, Oulu University Hospital, Oulu, Finland. [81]Munich Cluster for Systems Neurology (SyNergy), Munich, Germany. [82]Faculty of Medicine, University of Iceland, Reykjavik, Iceland. [83]Statistical and Genomic Epidemiology Laboratory, Institute of Health and Biomedical Innovation, Queensland University of Technology, Kelvin Grove, Brisbane, QLD, Australia. [84]Department of Neurology, Massachusetts General Hospital, Boston, MA, USA. [85]Institute of Translational Medicine, University of Liverpool, Liverpool, UK.
