# Supplementary material for: Genetic correlations of psychiatric traits with body composition and glycemic traits are sex- and age-dependent
Source: Nat Commun. 2019 Dec 18;10:5765. doi: 10.1038/s41467-019-13544-0 (PMC6920448; doi:10.1038/s41467-019-13544-0)
Supplement: Supplementary file 11 — Reporting Summary [file 41467_2019_13544_MOESM11_ESM.pdf]

nature research

Corresponding author(s): Christopher Hübel

Last updated by author(s): Aug 31, 2019

Reporting Summary

Nature Research wishes to improve the reproducibility of the work that we publish. This form provides structure for consistency and transparency in reporting. For further information on Nature Research policies, see Authors & Referees and the Editorial Policy Checklist.

Statistics

For all statistical analyses, confirm that the following items are present in the figure legend, table legend, main text, or Methods section.

n/a	Confirmed	
☐	☒	The exact sample size (*n*) for each experimental group/condition, given as a discrete number and unit of measurement
☐	☒	A statement on whether measurements were taken from distinct samples or whether the same sample was measured repeatedly
☐	☒	The statistical test(s) used AND whether they are one- or two-sided<br>*Only common tests should be described solely by name; describe more complex techniques in the Methods section.*
☐	☒	A description of all covariates tested
☐	☒	A description of any assumptions or corrections, such as tests of normality and adjustment for multiple comparisons
☐	☒	A full description of the statistical parameters including central tendency (e.g. means) or other basic estimates (e.g. regression coefficient) AND variation (e.g. standard deviation) or associated estimates of uncertainty (e.g. confidence intervals)
☐	☒	For null hypothesis testing, the test statistic (e.g. *F*, *t*, *r*) with confidence intervals, effect sizes, degrees of freedom and *P* value noted<br>*Give P values as exact values whenever suitable.*
☒	☐	For Bayesian analysis, information on the choice of priors and Markov chain Monte Carlo settings
☒	☐	For hierarchical and complex designs, identification of the appropriate level for tests and full reporting of outcomes
☐	☒	Estimates of effect sizes (e.g. Cohen's *d*, Pearson's *r*), indicating how they were calculated

Our web collection on statistics for biologists contains articles on many of the points above.

Software and code

Policy information about availability of computer code

Data collection	UK Biobank: www.ukbiobank.ac.uk
Data analysis	Analysis code can be accessed on github.com/topherhuebel/ukbgwas. And software can be accessed for BGENIE, at https://jmarchini.org/bgenie/; for BOLT-LMM v2.3.2, at https://data.broadinstitute.org/alkesgroup/BOLT-LMM/; for LDSC, at v1 https://github.com/bulik/ldsc; for METAL, at http://csg.sph.umich.edu/abecasis/metal/; for, at R 3.4 https://www.r-project.org/, for GSMR, at https://cnsgenomics.com/software/gcta/

For manuscripts utilizing custom algorithms or software that are central to the research but not yet described in published literature, software must be made available to editors/reviewers. We strongly encourage code deposition in a community repository (e.g. GitHub). See the Nature Research guidelines for submitting code & software for further information.

Data

Policy information about availability of data

All manuscripts must include a data availability statement. This statement should provide the following information, where applicable:

- Accession codes, unique identifiers, or web links for publicly available datasets
- A list of figures that have associated raw data
- A description of any restrictions on data availability

Supplementary Data 1 contains all information on data availability, including download links for summary statistics. Summary statistics for the body composition GWAS are available at www.topherhuebel.com/GWAS and the GWAS catalog (www.ebi.ac.uk/gwas/). Most summary statistics for psychiatric disorders are available at www.med.unc.edu/pgc/results-and-downloads/ and for glycemic traits at https://www.magicinvestigators.org/. The data that support the findings of this study are available from UK Biobank (www.ukbiobank.ac.uk). Restrictions apply to the availability of these data, which were used under license for the current study (Project ID: 27546). Data are available for bona fide researchers upon application to the UK Biobank.

Field-specific reporting

Please select the one below that is the best fit for your research. If you are not sure, read the appropriate sections before making your selection.

☒ Life sciences ☐ Behavioural & social sciences ☐ Ecological, evolutionary & environmental sciences

For a reference copy of the document with all sections, see nature.com/documents/nr-reporting-summary-flat.pdf

Life sciences study design

All studies must disclose on these points even when the disclosure is negative.

Sample size	Power calculations of the genome-wide association studies<br>We conducted power calculations for the female and male GWAS using the Genetic Power Calculator (Purcell, Cherny, & Sham, 2003). Power of 80% at a genome-wide significance threshold of p≤5x10-8 and a MAF 0.20 to detect a SNP that accounts for 0.1% of trait variance requires 39,580 individuals. According to these results the female and the male GWAS were sufficiently powered to detect genome-wide significant loci with 70,700 females and 85.261 males. With this parameters, the female GWAS had a power of 99.8% and the male GWAS of 99.9%.
Data exclusions	We applied trait-specific medication and illness filtering to exclude participants with compromised hydration status and drugs or illnesses known to affect body composition (for exclusion criteria, see Supplementary Table 3).
Replication	N/A
Randomization	N/A
Blinding	N/A

Reporting for specific materials, systems and methods

We require information from authors about some types of materials, experimental systems and methods used in many studies. Here, indicate whether each material, system or method listed is relevant to your study. If you are not sure if a list item applies to your research, read the appropriate section before selecting a response.

Materials & experimental systems

n/a	Involved in the study	
☒	☐	Antibodies
☒	☐	Eukaryotic cell lines
☒	☐	Palaeontology
☒	☐	Animals and other organisms
☐	☒	Human research participants
☒	☐	Clinical data

Methods

n/a	Involved in the study	
☒	☐	ChIP-seq
☒	☐	Flow cytometry
☒	☐	MRI-based neuroimaging

Human research participants

Policy information about studies involving human research participants

Population characteristics	UK Biobank (www.ukbiobank.ac.uk) is a unique epidemiological and prospective resource to generate research findings aimed to improve prevention, diagnosis, and treatment of psychiatric and somatic illnesses. Genomewide array data for this study were available for 488,363 individuals. Due to this trait-specific medication and illness filtering, the final analysis included 155,961 (45% female) healthy and drug-free European participants which are 32% of the genotyped UK Biobank participants (n = 502,682). This study has been completed under UK Biobank approved study application 27546.
Recruitment	UK Biobank recruited participants from the general population between 2006–2010. All participants were between 40 to 69 years old, were registered with a general practitioner through the United Kingdom's National Health Service, and lived within traveling distance of one of the assessment centres.
Ethics oversight	The UK Biobank is approved by the North West Multi-centre Research Ethics Committee. All procedures performed in studies involving human participants were in accordance with the ethical standards of the North West Multi-centre Research Ethics Committee and with the 1964 Helsinki Declaration and its later amendments or comparable ethical standards. All participants provided written informed consent to participate in the study. This study has been completed under UK Biobank approved study application 27546.

Note that full information on the approval of the study protocol must also be provided in the manuscript.
