# Supplementary material for: Genetic correlations of psychiatric traits with body composition and glycemic traits are sex- and age-dependent
Source: Nat Commun. 2019 Dec 18;10:5765. doi: 10.1038/s41467-019-13544-0 (PMC6920448; doi:10.1038/s41467-019-13544-0)
Supplement: Supplementary file 12 — Description of Additional Supplementary Files [file 41467_2019_13544_MOESM12_ESM.pdf]

Title: Supplementary Data 1.
Description: Overview over genome-wide association studies included in this study. This file contains data availability and download links.

Title: Supplementary Data 2.
Description: Genetic correlations between body composition traits, physical activity, childhood overweight, and psychiatric disorders and behavioural traits.

Title: Supplementary Data 3.
Description: Differences in genetic correlations of body composition, glycemic traits, and physical activity with psychiatric and behavioural traits between females and males.

Title: Supplementary Data 4.
Description: Forward generalised summary data-based Mendelian randomisation (GSMR) of psychiatric traits as exposures and body composition traits as outcomes.

Title: Supplementary Data 5.
Description: Reverse generalised summary data-based Mendelian randomisation (GSMR) of body composition traits as exposures and psychiatric traits as outcomes.

Title: Supplementary Data 6.
Genetic correlations between glycemic traits and psychiatric disorders and behavioural traits.

Title: Supplementary Data 7.
Description: Genetic correlations that are not adjusted for smoking or alcohol consumption between body composition traits, physical activity, childhood overweight, and psychiatric disorders and behavioural traits.

Title: Supplementary Data 8.
Description: Genetic correlations between fat-free mass and body mass index in childhood and young adulthood, childhood overweight, and psychiatric disorders and behavioural traits.
